# Genes whose expressions in the primary lung squamous cell carcinoma are able to accurately predict the progression of metastasis through lymphatic system, inferred from a bioinformatics analyses

**DOI:** 10.1038/s41598-023-33897-3

**Published:** 2023-04-25

**Authors:** Khalil Khashei Varnamkhasti, Mehdi Moghanibashi, Sirous Naeimi

**Affiliations:** 1 Department of Genetics, College of Science, Islamic Azad University, Kazerun Branch, Kazerun, Iran; 2 Department of Genetics, Faculty of Medicine, Islamic Azad University, Kazerun branch, Kazerun, Iran

**Keywords:** Genetics, Molecular biology, Cancer

## Abstract

Lymph node metastasis is the most important prognostic factor in patients with lung squamous cell carcinoma. The current findings show that lymph node metastatic tumor cells can arise by programming metastasis in primary tumor cells. Thereby, the genetic alterations responsible for the metastasis could be detected in the primary tumors. This bioinformatic study aimed to determine novel potential prognostic biomarkers shared between primary lung squamous cell tumors (without lymph node metastasis) and lymphatic metastasis, using the Cancer Genome Atlas database. Differentially expressed genes were screened by limma statistical package in R environment. Gene ontology and biological pathways analyses were performed using Enrichr for up-regulated and down-regulated genes. Also, we selected lymph node metastasis related genes among DEGs using correlation analysis between DEGs and suitable references genes for metastasis. Receiver operating characteristic curves was applied using pROC and R package ggplot2 to evaluate diagnostic value of differentially expressed genes. In addition, survival and drug resistance analyses were performed for differentially expressed genes. The miRNA-mRNA interaction networks were predicted by miRwalk and TargetScan databases and expression levels analysis of the miRNAs which were mainly targeting mRNAs was performed using UALCAN database. Protein–protein interaction network analysis and hub genes identification were performed using FunRich and Cytoscape plugin cytoHubba. In this study, a total of 397 genes were differentially expressed not only with a significant difference between N + vs. normal and N0 vs. normal but also with significant difference between N + vs. N0. Identified GO terms and biological pathways were consistent with DEGs role in the lung squamous cell carcinoma and lymph node metastasis. A significant correlation between 56 genes out of 397 differentially expressed genes with reference genes prompted them being considered for identifying lymph node metastasis of lung squamous cell carcinoma. In addition, *SLC46A2*, *ZNF367*, *AC107214.1* and *NCBP1* genes were identified as survival-related genes of patients with lung squamous cell carcinoma. Moreover, *NEDD9*, *MRPL21*, *SNRPF*, and *SCLT1* genes were identified to be involved in lung squamous cell carcinoma drug sensitivity/resistance. We have identified several numbers of miRNAs and their related target genes which could emerge as potential diagnostic biomarkers. Finally, *CDK1*, *PLK1*, *PCNA*, *ZWINT* and *NDC80* identified as hub genes for underlying molecular mechanisms of lung squamous cell carcinoma and lymphatic metastasis. Our study highlights new target genes according to their relation to lymph node metastasis, whose expressions in the primary lung squamous cell carcinoma are able to accurately assess the presence of lymphatic metastasis.

## Introduction

Lung Squamous cell carcinoma (LUSC) is a common subtype of non-small cell lung cancer (NSCLC), accounting for 20% of all cases and causing more than 400,000 new cases worldwide each year^1,2^. In general, LUSC tends to be aggressive, and 60% of patients are diagnosed with local and distant invasion^3–5^. Metastasis of LUSC is a major cause of mortality, often presenting diagnostic challenges and is the main drawback of successful treatments^6,7^.

During metastasis, malignant cells may invade through the blood or lymphatic vessels, however, spread to the hilar or interlobar lymph nodes (N1), then to the ipsilateral (N2), or contralateral mediastinal lymph nodes (N3) is the primary route for LUSC and other NSCLC histologic subtypes metastasis, which facilitate tumor distant metastasis^6,8,9^. Lymphatic vessels with slower flow rate along with reduce shear stress due to impaired lymphatic vessels contraction increase the tumor cells survival more than systematic vessels. In addition, stagnation lymph flow can directly connect cancer cells to each other, as well as activate the integrin-mediated signaling pathway and increase tumor growth in the vessel^10^. Also, there are many gap junctions in the discontinuous basement membrane, which causes leakage of lymphatic vessels and metastatic spread of tumor cells^11^. Accordingly, the prognosis of LUSC greatly depends on the presence of lymph node metastasis (LNM)^6^. It has recently been suggested that although the metastasis appears in the later stages of cancer, the initiation of metastatic programming occurs much earlier in the primary tumor cells^12,13^.

Genetic alterations in some of the tumor cells in the primary tumor give them advantages that enable them to form large populations in the primary tumor mass. These beneficial traits include acquisition of mitogenic signal propagation, capability to resist growth inhibiting signals, the ability to prevent apoptosis or programmed cell death, and lymphangiogenesis growth induction. A subset of these genetic changes, acquired by cells in the early stages of tumorigenesis confer the ability to metastasis later. It seems that there is a genetic programing to enable cancer cells in the primary tumors to metastasize^14–16^. Interestingly, some of the genomic aberrations required for progression and metastatic spread identified in LUSC patients with lymph node metastasis correspond to aberrations that impair the function of growth regulatory proteins and inactive growth inhibitory pathways in primary LUSC^17^. Therefore, the identification of molecular biomarkers in the early stages of tumor, whose expressions are able to accurately predict the progression to lymph node metastasis, is strongly needed^18,19^.

Recent advances in molecular oncology have described some coding and non-coding genes including micro RNAs (miRNAs), as molecular biomarkers in the early cancer detection and prognostic markers^20^. Uncovering biomarkers whose differential expression in primary tumor is strongly associated with the potential LUSC tumor progression could improve the prognosis by developing novel and groundbreaking therapeutic approaches that target specific genetic alterations in primary tumors.

Identification of key genes involved in molecular mechanism of cancer, especially for metastasis and lymph node metastasis, has been addressed in certain genetic studies^21,22^, and in some of them it may even have been shown that the same genes are shared between the primary and metastatic lesions. However, to our knowledge, there are no studies that identify genes involved not only in growth and development of tumor but also in lymph node metastasis in LUSC. Hence, the present bioinformatic study aimed to determine new molecular pathways and potential novel prognostic biomarkers expressed differentially between N + and N0 compared to normal, which were also significantly expressed differentially in N + compared to N0 in LUSC using The Cancer Genome Atlas (TCGA) database.

## Methods

### Data collection and preparation

Approval for this bioinformatics-based study was obtained from the Islamic Azad University- Kazerun Branch Ethics Committee (IR.IAU.KAU.REC.1400.141). All methods were performed in accordance with the guidelines and regulations of the Islamic Azad University- Kazerun Branch.

**Figure 1:**
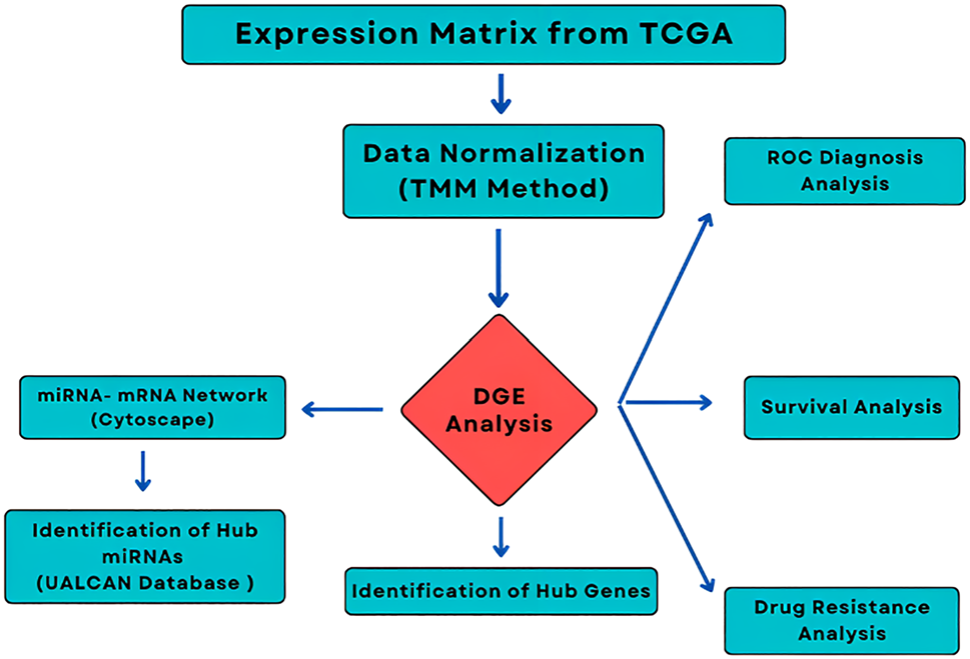
The flow chart of the present study for data collection, processing and analysis.

In this study, the Cancer Genome Atlas Lung Squamous Cell Carcinoma (TCGA-LUSC) data (Accession number: caArray_EXP-592) were downloaded from LinkedOmics (http://linkedomics.org/). All genes with insignificant and close to zero expression levels were removed from the matrix using the counts per million (CPM) method. Also, normalization was performed using the trimmed mean of the m-values (TMM) method, and all values were converted to log2 scale. The flowchart is presented in Fig. 1.

### Differential gene expression analysis

To perform differential gene expression (DGE) analysis, linear model of the limma package in the R environment was used. All samples were divided into three groups: patients with N + (including N1, N2 and N3) metastasis (n = 176), patients without lymph node metastasis or N0 condition (n = 320) and normal tissue samples (n = 49). DGE was performed between N + vs. normal, N0 vs. normal and N + vs. N0. The selection criteria were the adj. p value < 0.05 and log fold change was not considered. Genes were selected as DEGs in final that were not only significantly deregulated in N + and N0 compared to normal, but also deregulated in N + compared to N0.

### Signaling pathway and gene ontology analyses

Pathway analysis was done using Enrichr online web tool, comprising analyses of REACTOME pathways and Wikipathways (https://amp.pharm.mssm.edu/Enrichr). Furthermore, we performed Gene ontology (GO) function analysis including the cellular component (CC), molecular function (MF), and biological process (BP), and Kyoto Encyclopedia of Genes and Genomes (KEGG) pathway enrichment analysis on the differentially expressed genes.

### Genes selection for lymph node metastasis

First, we conducted a literature search to find suitable reference genes for lymph node metastasis in lung cancer by searching PubMed, Google Scholar, Embase, ScienceDirect and Cochrane Library with the keywords: “Metastasis”, “Gene Expression”, “Lung cancer”and “Lymph node metastasis”. For example, the search query in PubMed was (Metastasis [MeSH Terms]) AND (Gene Expression [MeSH Terms]) AND (Lung cancer [MeSH Terms]).Then determined the correlation coefficient (CC) between all the differentially expressed genes with the reference genes. Differentially expressed genes with a correlation coefficient range of > 0.4 or < − 0.4 with the reference genes were selected as lung cancer progression and lymph node metastasis.

### Clustering and ROC analysis

DE genes presented a positive correlation with the reference genes were clustered through the kmeans method to draw a heat map. We divided candidate genes into the number of groups equal to the number of clusters and applied Receiver Operating Characteristic** (**ROC) curve analysis using the pROC package to examine how each gene in each cluster could distinguish cancer from normal samples. In addition, the calculated sensitivities and specificities were used to draw ROC curves through the ggplot2 R package.

### Survival analysis

First, the clinical data including T-stage, N-stage, pathological-stage, gender, tobacco smoking history, age, state of living, and follow-up time were added to the expression matrix. Then, all expression values of each gene were scaled between zero and one, and these scaled values were rounded into 1–10 to have ten gene expression levels. Next, the univariate Cox regression analysis was performed on each gene to evaluate their role in the patient survival (log rank test < 0.05) also, the same analysis was performed on them for survival-related clinical data.

Selected genes from univariate Cox regression analysis of identified DE genes were used to compute a risk score model to assess the patients' survival rate with the following formula:

$$Risk \,score=\sum_{j=1}^{n}{W}_{j}\times {exp}_{ij}$$

where W_j_ is the multivariate coefficient for gene j, Exp_ij_ is the expression value of gene j in patient i, and n is the number of testing genes.

In addition, multivariate cox regression analysis was performed considering significant clinical parameters and risk scores. Patients were divided into high and low-risk groups based on the average risk score as the cut-off value and finally the survival plots were depicted based on each gene and model genes.

### Drug resistance/sensitivity analysis

Drug resistance analysis was performed using the PharmacoGX package in R which is connected to various public databases to find out which candidate genes are related to cell drug response. We used lung samples from Cancer Cell Line Encyclopedia (CCLE) to perform our analysis. The association between expression of each gene and drugs was calculated based on IC50 and other parameters such as estimate, p.value, and the number of samples was also calculated. The estimate measures the correlation of each gene to sensitivity or resistance to each drug. The number is between − 1 and 1; − 1 means that the higher gene expression causes the higher sensitivity to the specific drug, while 1 indicates the highest resistance to the drug based on the increased gene expression. Only Food and Drug Administration (FDA)-approved drugs were checked in this study (p value less than 0.05).

### Analysis of mRNA- miRNAs interaction

For all identified DE genes, possible interactions with miRNAs were checked through TargetScan (https://www.targetscan.org/) and miRWalk (http://mirwalk.umm.uni-heidelberg.de/) databases and those miRNAs that could pass double-checking process in both databases, were selected. Cytoscape v 3.7 was applied to visualize miRNA-mRNA interaction networks. In addition, we used the UALCAN database (http://ualcan.path.uab.edu)^23^ to analyze expression levels of the miRNAs which were targeting any more mRNAs in LUSC.

### Identification of hub genes

First, FunRich tool version 3.1.3^24^ was applied to illustrate the interaction of differentially expressed genes. Then, hub genes were selected using the Cytoscape plugin cytoHubba. Protein–protein interaction network of these hub genes were constructed by STRING (http://string-db.org; version 10.5) and visualized by Cytoscape.

Moreover, to identify critical upstream signals that lead to lymph node metastasis we expanded the network by adding Human Protein Reference Database (HPRD)-derived direct interacting partners.

### Soft wares and statistics

All statistical and in-silico analyses were performed in R environment version 4.0.1 and 0.05 was considered as cut-off p.value in all steps of the study. Cytoscape version 3.9.1, GraphPad Prism version 9, FunRich version 3.1.3 and cytoHubba, were used for network analysis and visualization.

### Informed consent

This work is not involving “human participants” because in this bioinformatics-based study, as a Secondary Research collected information for another primary activity has been used. Generally speaking, all samples in TCGA have been collected and utilized following strict human subjects protection guidelines, informed consent and IRB review of protocols.

## Results

### Identification of DEGs

**Figure 2:**
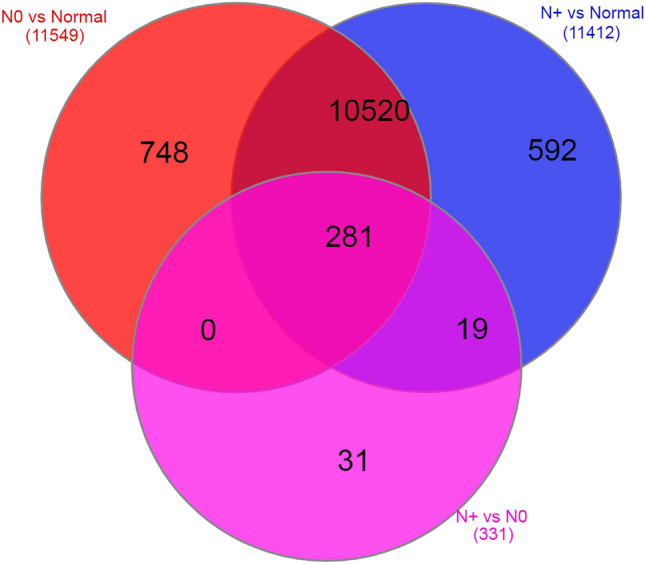
Identification of dedicated and overlapping up-regulated DEGs related to N + and N0 conditions.

**Figure 3:**
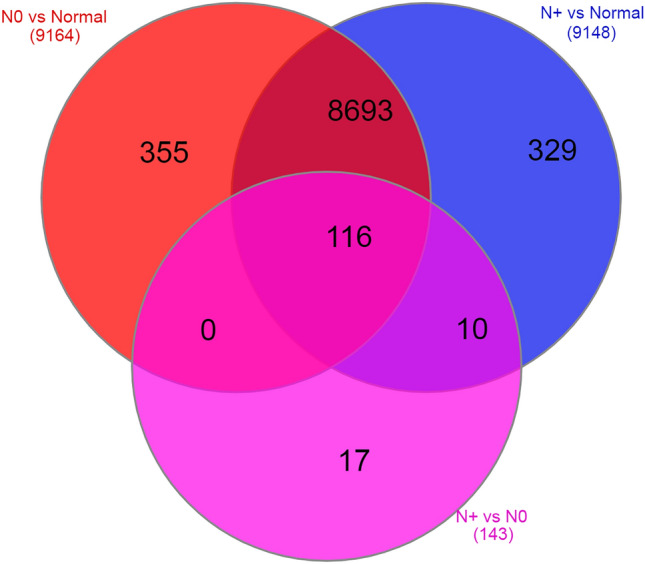
Identification of dedicated and overlapping down-regulated DEGs related to N + and N0 conditions.

A total of 397 genes were significantly showed expressed differentially between N + and N0 compared to normal, which were also significantly expressed differentially in N + compared to N0 (including 281 upregulated (Fig. 2, Supplementary File 1) and 116 genes downregulated (Fig. 3, Supplementary File 2)).

### Reactome /GO/KEGG analysis for all identified DEGs

We utilized Enrichr web tools to identifying possible signaling pathways for all 397 DE genes. In REACTOME analysis (Table 1), the most outstanding pathway for down-regulated DEGs was “surfactant metabolism pathway” which provides valuable insights into the pathways implicated in lung cancer tumor-infiltrating lymphocytes. From the results of Wikipathway analysis for down-regulated DEGs (Table 2), the most highly correlated pathway was “Lung fibrosis”. There is only one cellular component ontology of down-regulated DEGs enriched categories, which were associated with alveolar lamellar body (Table 3).

**Table 1:** Reactome pathway down-regulated DEGs enrichment analysis.

	Description	p-value	Adjusted p-value	Odds ratio	Combined score
1	Defective CSF2RA causes SMDP4 R-HSA-5688890	6.713E − 06	0.001269	130.78	1557.82
2	Diseases associated with surfactant metabolism R-HSA-5687613	0.00001597	0.001486	87.18	962.86
3	Ficolins bind to repetitive carbohydrate structures on target cell surface R-HSA-2855086	0.0003354	0.01268	115.25	921.99
4	Lectin pathway of complement activation R-HSA-166662	0.0009284	0.02925	57.61	402.26
5	Surfactant metabolism R-HSA-5683826	0.00002359	0.001486	28.12	299.58

**Table 2:** Wikipathway down-regulated DEGs enrichment analysis.

	Description	p-value	Adjusted p-value	Odds ratio	Combined score
1	Complement activation WP545	0.0002770	0.02133	27.51	225.36
2	Lung fibrosis WP3624	0.0005078	0.02133	11.89	90.22
3	TFs regulate miRNAs related to cardiac hypertrophy WP1559	0.04586	0.3898	24.48	75.44
4	Complement and coagulation cascades WP558	0.004764	0.1001	9.49	50.72
5	NAD metabolism, sirtuins and aging WP3630	0.06252	0.3898	17.13	47.50

**Table 3:** Gene ontology cellular component-based enrichment analysis of down-regulated DEGs.

	Description	p-value	Adjusted p-value	Odds ratio	Combined score
1	Alveolar lamellar body (GO:0097208)	0.0005012	0.01854	86.43	656.74
2	Lamellar body (GO:0042599)	0.00005347	0.005935	52.30	514.42
3	Multivesicular body lumen (GO:0097486)	0.0006990	0.01917	69.14	502.37
4	Late endosome lumen (GO:0031906)	0.001189	0.02200	49.38	332.56
5	Multivesicular body (GO:0005771)	0.0001634	0.009068	16.33	142.41

Gene enrichment analysis results of up-regulated DEGs using the Reactome Pathway (Table 4), Wikipathway (Table 5), KEGG Pathway (Table 6), Gene ontology Biological Process, Gene ontology Cellular Component and Gene ontology Molecular Function (Table 7), mainly concentrated in “mitotic division,” and “cell cycle”.

**Table 4:** Reactome pathway up-regulated DEGs enrichment analysis.

	Description	p-value	Adjusted p-value	Odds ratio	Combined score
1	G2/M DNA replication checkpoint R-HSA-69478	1.913e − 7	0.000002259	283.70	4388.59
2	Polo-like kinase mediated events R-HSA-156711	1.651e − 11	4.444e − 10	71.93	1785.92
3	Cell cycle R-HSA-1640170	3.080e − 50	2.073e − 47	12.71	1448.61
4	Resolution Of sister chromatid cohesion R-HSA-2500257	3.441e − 25	3.309e − 23	24.93	1404.34
5	Cell cycle, mitotic R-HSA-69278	7.137e − 45	2.402e − 42	13.16	1338.09

**Table 5:** Wikipathway up-regulated DEGs enrichment analysis.

	Description	p-value	Adjusted p-value	Odds ratio	Combined score
1	Regulation of sister chromatid separation at the metaphase-anaphase transition WP4240	6.009e − 10	3.565e − 8	62.71	1331.57
2	Retinoblastoma gene in cancer WP2446	1.265e − 12	2.252e − 10	15.33	419.96
3	DNA Replication WP466	1.102e − 7	0.000003923	16.90	270.81
4	Gastric Cancer Network 1 WP2361	0.000002694	0.00006851	18.62	238.73
5	Cell cycle WP179	1.500e − 10	1.335e − 8	10.49	237.37

**Table 6:** KEGG pathway based enrichment analysis of up-regulated DEGs.

	Description	p-value	Adjusted p-value	Odds ratio	Combined score
1	Homologous recombination	9.030e − 8	0.000002799	17.42	282.50
2	Cell cycle	2.216e − 11	2.747e − 9	10.92	267.94
3	DNA replication	6.050e − 7	0.00001250	17.28	247.44
4	Fanconi anemia pathway	5.918e − 8	0.000002446	14.41	239.86
5	Progesterone-mediated oocyte maturation	1.704e − 8	0.000001056	9.91	177.34

**Table 7:** Gene ontology biological process, molecular function, and cellular component-based enrichment analysis of up-regulated DEGs.

Biological process	Description	p-value	Adjusted p-value	Odds ratio	Combined score
1	Microtubule cytoskeleton organization involved in mitosis (GO:1902850)	1.507e − 25	2.110e − 22	21.63	1236.03
2	Kinetochore organization (GO:0051383)	1.177e − 8	9.152e − 7	61.21	1117.65
3	Sister chromatid segregation (GO:0000819)	7.733e − 13	1.547e − 10	34.76	969.33
4	Mitotic spindle organization (GO:0007052)	9.965e − 22	6.976e − 19	15.95	771.50
5	Regulation of sister chromatid cohesion (GO:0007063)	3.929e − 7	0.00001618	50.83	749.70

Molecular function		p-value	Adjusted p-value	Odds ratio	Combined score
1	RNA–DNA hybrid ribonuclease activity (GO:0004523)	0.00002716	0.0008540	106.00	1114.46
2	Histone serine kinase activity (GO:0035174)	0.00005375	0.001383	70.66	694.70
3	Histone kinase activity (GO:0035173)	0.0001474	0.002781	42.39	374.02
4	5'-flap endonuclease activity (GO:0017108)	0.0001474	0.002781	42.39	374.02
5	Damaged DNA binding (GO:0003684)	8.924e − 9	0.000002526	18.54	343.62

Cellular component		p-value	Adjusted p-value	Odds ratio	Combined score
1	Condensed chromosome, centromeric region (GO:0000779)	0.000007600	0.00007195	47.27	557.20
2	Spindle microtubule (GO:0005876)	2.370e − 12	1.122e − 10	19.80	530.12
3	Condensed chromosome (GO:0000793)	5.918e − 8	7.003e − 7	14.41	239.86
4	Mitotic spindle (GO:0072686)	1.500e − 10	3.044e − 9	10.49	237.37
5	Spindle (GO:0005819)	3.832e − 12	1.360e − 10	8.67	228.04

### Identification of lymph node metastasis diver genes among all DEGs

We retrieved 143 articles related to lung cancer lymph node metastasis following strictly filtering based on exclusion criteria (conference abstracts, letters, and animal model studies were excluded) by searching PubMed, Google Scholar, Embase, Sci-enceDirect and Cochrane Library. Eventually, a total of four genes including, CD151, MMP1, PVT1, and SKP2 genes were selected as reference genes based on their known functions in lung cancer progression and lymph node metastasis which have been pointed out in the found articles. CD151 is a member of the transmembrane 4 superfamily, also known as the tetraspanin family. It is involved in cellular processes including cell adhesion and enhances cell motility, invasion and metastasis of cancer cells. MMP1 is a member of zinc-dependent endopeptide proteases family, which is prominently associated with extracellular matrix destruction (a genetic alteration responsible for programming metastasis in primary tumors) that is critical to the development of a primary tumor and its metastatic progeny. The PVT1 gene is known as an oncogene, and its overexpression is associated with many types of cancers, including breast and ovarian cancers. The *SKP2* gene encodes a member of the F-box protein family, this gene is established as a protooncogene involved in the pathogenesis of lymphomas (Supplementary File 3).

**Figure 4:**
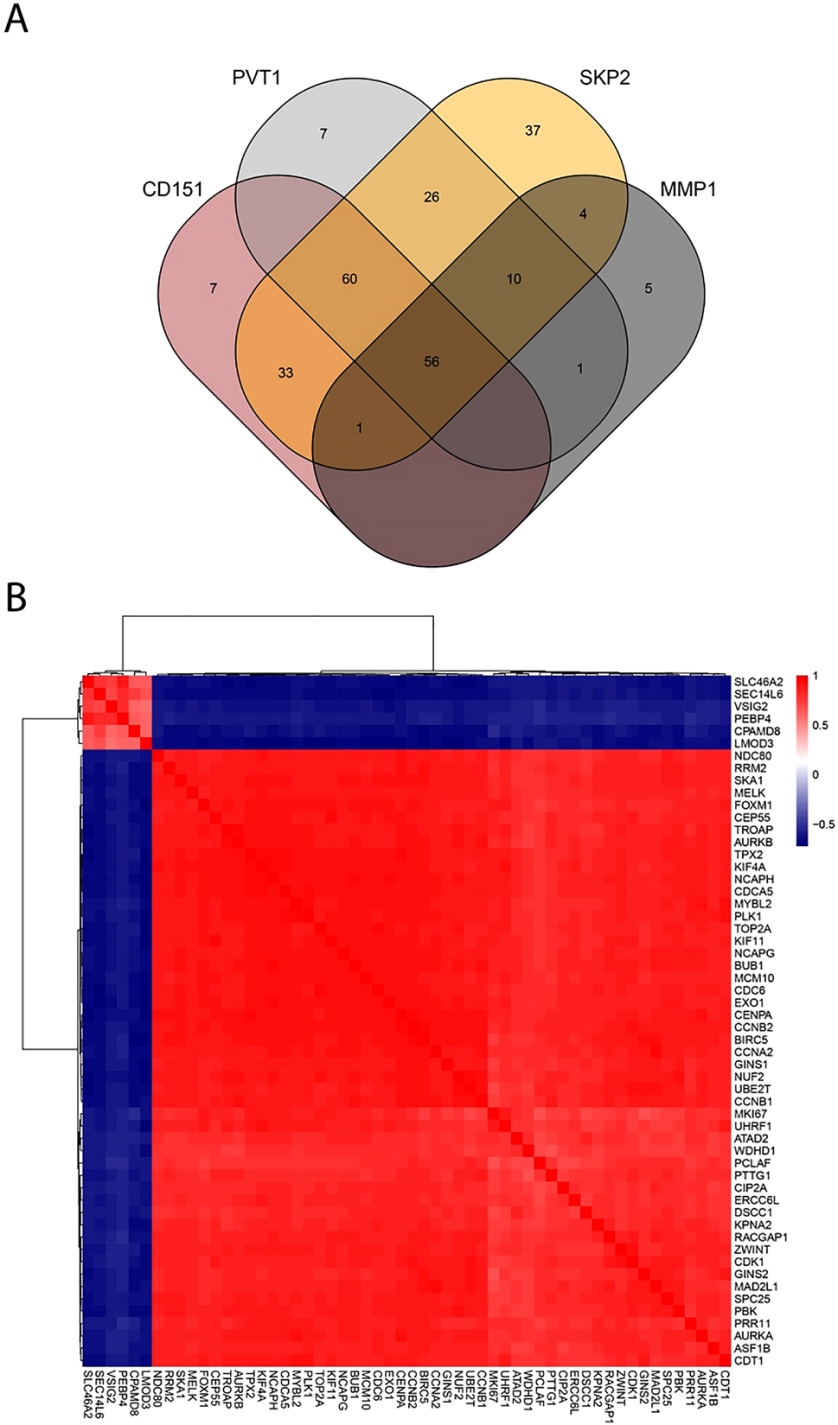
Genes that are significantly expressed differentially between N + and N0 compared to normal, which were also significantly expressed differentially in N + compared to N0. (**A**) Venn diagram of the significantly correlated DEGs with the reference genes, (**B**) 56 candidate genes are significantly correlated with *CD151*, *MMP1, PVT1, and SKP2* and divided into two clusters.

Correlation results showed that out of 397 differentially expressed genes in our study, 157 were highly correlated to CD151, 77 with MMP1, 160 with PVT1, and 227 with SKP2 (CC > 0.4 or < − 0.4). 56 of them were shared between all four reference genes (Supplementary Table 1, Fig. 4A) and selected as lymph node metastasis driver genes.

In addition, Kmeans clustering results indicated that these 56 genes could be divided into two up-regulated (with 50 genes) and down-regulated (with 6 genes) clusters, and the correlation between genes and clusters was shown by heatmap (Fig. 4B).

**Figure 5:**
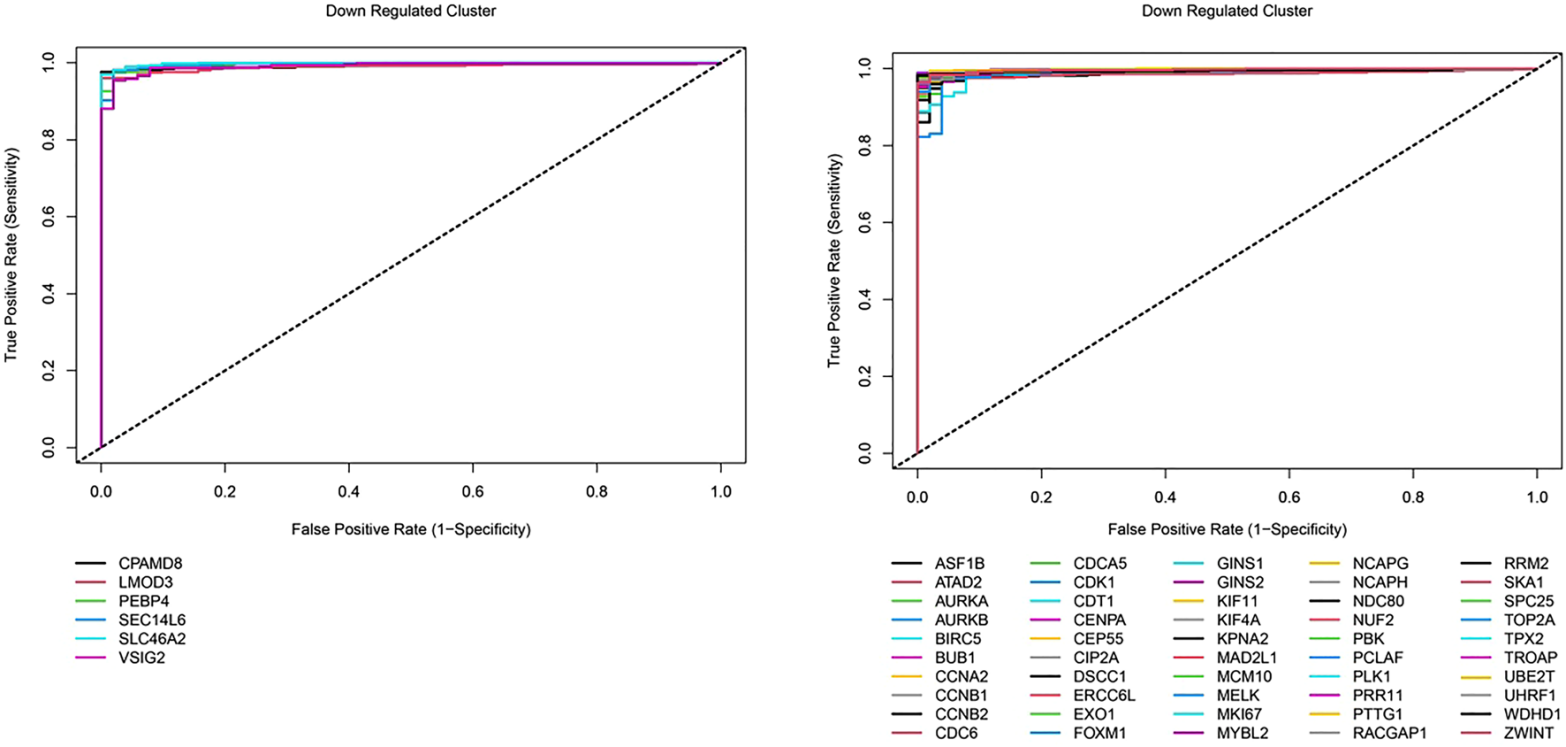
ROC curves of two clusters show high AUC the candidate genes. The ROC curves of all genes in each cluster are shown.

The ROC analysis was performed for each cluster (Fig. 5) and have shown that these genes could be used as excellent potential biomarkers (AUC > 0.9) (Supplementary Table 2).

### A four‐gene‐based prognostic model may influence patient survival

The univariate cox regression results indicated that 45 of 397 DE genes might affect patients' survival. On the other hand, only T-stage, N-stage, tobacco smoking history, and pathological-stage showed a significant association with survival among clinical parameters (Table 8). Also, multivariate CoxPH analysis was performed and results showed that *SLC46A2*, *ZNF367*, *AC107214.1* and *NCBP1* genes can significantly affect survival independent of clinical parameters (Table 9). Also, T-stage and N-stage and pTNM showed p value < 0.05 in multivariate results (Table 9).

**Table 8:** Univariate CoxPH results for clinical parameters.

	Beta	HR (95% CI for HR)	p value
Gender	0.052	1.1 (0.73–1.5)	0.78
T_stage	0.27	1.3 (1.1–1.6)	0.0078
M_stage	0.33	1.4 (0.34–5.7)	0.65
N_stage	0.23	1.3 (1–1.6)	0.04
Tobacco_smoking_history	− 0.18	0.84 (0.71–1)	0.045
Age	0.017	1 (1–1)	0.1
P_stage	0.23	1.3 (1–1.5)	0.019

**Table 9:** Multivariate Cox Regression Results.

	HR	Lower 95	Upper 95	Beta	Pr( >|z|)
*SLC46A2*	1.298838	1.015962	1.814551	0.206294	0.039603
*ZNF367*	1.57952	1.012354	1.725914	0.262508	0.042501
*AC107214.1*	1.4138555	1.007441	1.439582	0.189982	0.0352
*NCBP1*	0.8302	0.73102	0.943729	− 0.14982	0.024503
T_stage	1.375873	1.016603	2.016004	0.362092	0.047203
N_stage	1.776052	1.015049	2.867193	0.632813	0.03421
Risk score	1.613912	1.011469	2.116063	0.668662	2.073E − 05
pTNM	1.3501	1.031	1.768	0.300164	0.0291

The risk score models for these four genes were calculated with the following formula:

$${\text{Risk Score}}\,{ = }\,{0}{\text{.2463}} \times {\text{EXPSLC46A2}}\,{ + }\,{0}{\text{.2191}} \times {\text{EXPZNF367}}\,{ + }\,{0}{\text{.134}} \times {\text{EXPAC107214}}{.1} - {0}{\text{.2842}} \times {\text{EXPNCBP1}}$$

**Figure 6:**
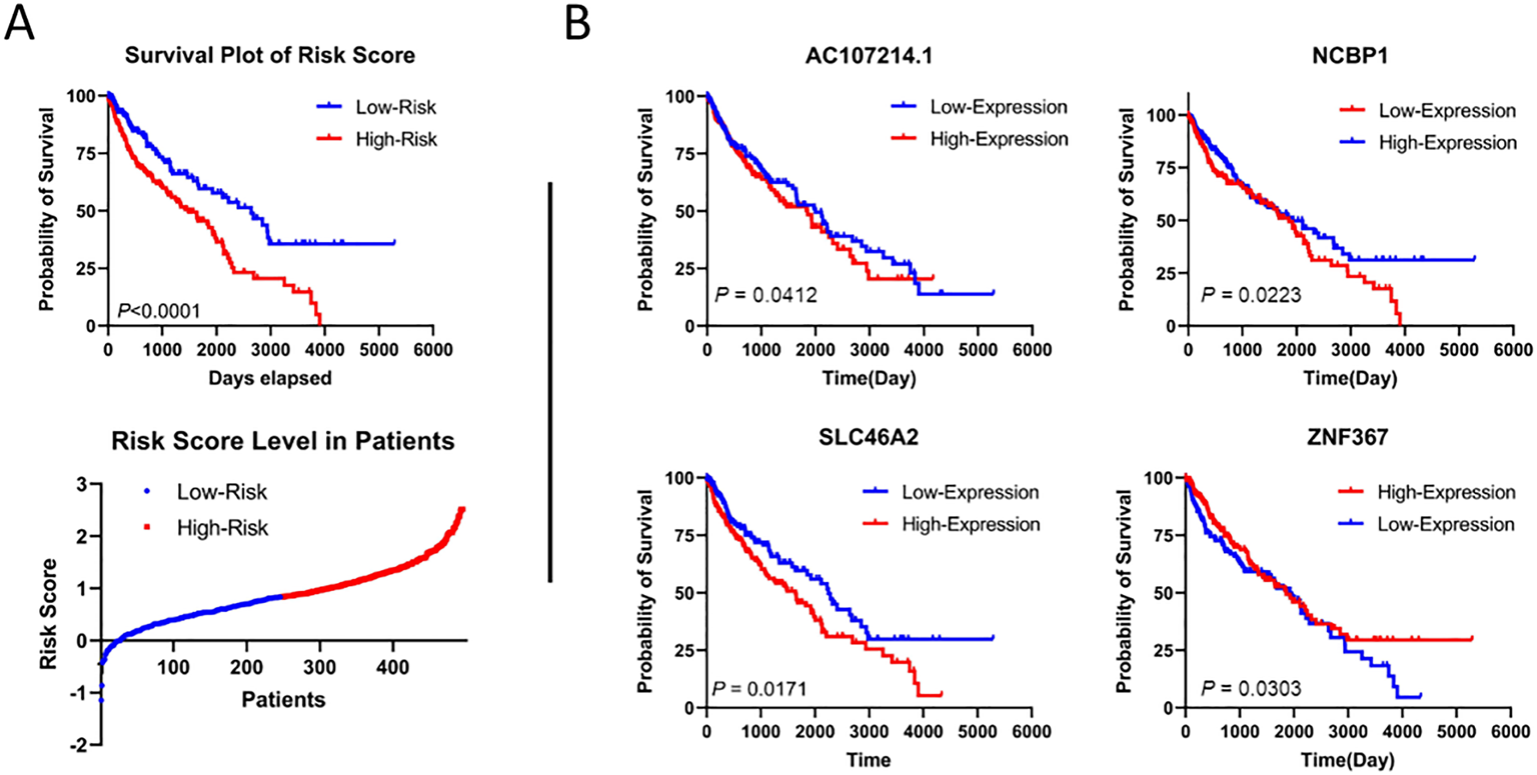
Classification of patients into two high and low-risk groups based on risk score model consisting of four genes and their survival rate. (**A**) The survival plot of high and low-risk patients based on median risk score and the risk score distribution of patients as a scatter plot. (**B**) The survival plot of each gene applied to compute the risk score based on each gene's high and low expression levels, using their median as the cut-off value.

And, was performed for each patient to obtain another multivariate Cox regression analysis to see whether it can independently impact survival. The results revealed that the risk score could significantly affect the survival of patients (p value < 0.0001). Moreover, we tested the model by dividing patients into high and low-risk groups and drawing their survival plots (Fig. 6A). Also, the survival plot for each gene in two conditions of high expression and low expression is shown in Fig. 6B.

Mainly, survival analyses showed that the same expression of SLC46A2, ZNF367, AC107214.1 and NCBP1 genes in the primary and LNM lesions can provides more potent prognostic information when no analyses of the primary tumor have been done. This prediction gene signature of prognosis in early LUSC will support treatment decision-making.

### Contribution of five crucial genes to Dovitinib and Paclitaxel resistance/sensitivity in LUSC

We used the CCLE database to assess the association of 397 DE genes with drugs used for lung cancer chemotherapy applying PharmacoGX package in the R environment. IC50 was used to compare the expression of each gene for each drug and calculate a False Discovery Rate (FDR). Our parameter called estimate shows the association of each drug and gene with a number in the range of − 1 and 1 (1 shows the highest resistance and − 1 shows the highest sensitivity). We identified abnormal expression *NEDD9* gene confer resistance to Dovitinib with an estimate value of 0.77. On the other hand, high expression of *SNRPF* and *SCLT1* genes cause sensitivity to Paclitaxel with estimate values of around − 0.7. However, *MRPL21* might cause sensitivity to both drugs, with the estimate value around − 0.7 (Table 10).

**Table 10:** Potential genes involved in LUSC drug resistance/sensitivity.

	Estimate	n	p value	fdr	Drugname	Gene symbol
*ENSG00000111859*	0.770216	17	0.000298	0.043216	Dovitinib	*NEDD9*
*ENSG00000197345*	− 0.76803	17	0.000318	0.044781	Dovitinib	*MRPL21*
*ENSG00000139343*	− 0.69455	22	0.000335	0.044422	Paclitaxel	*SNRPF*
*ENSG00000151466*	− 0.69171	22	0.000363	0.045954	Paclitaxel	*SCLT1*
*ENSG00000197345*	− 0.7169	22	0.000174	0.02772	Paclitaxel	*MRPL21*

### Construction of mRNAs-miRNAs interaction network

**Figure 7:**
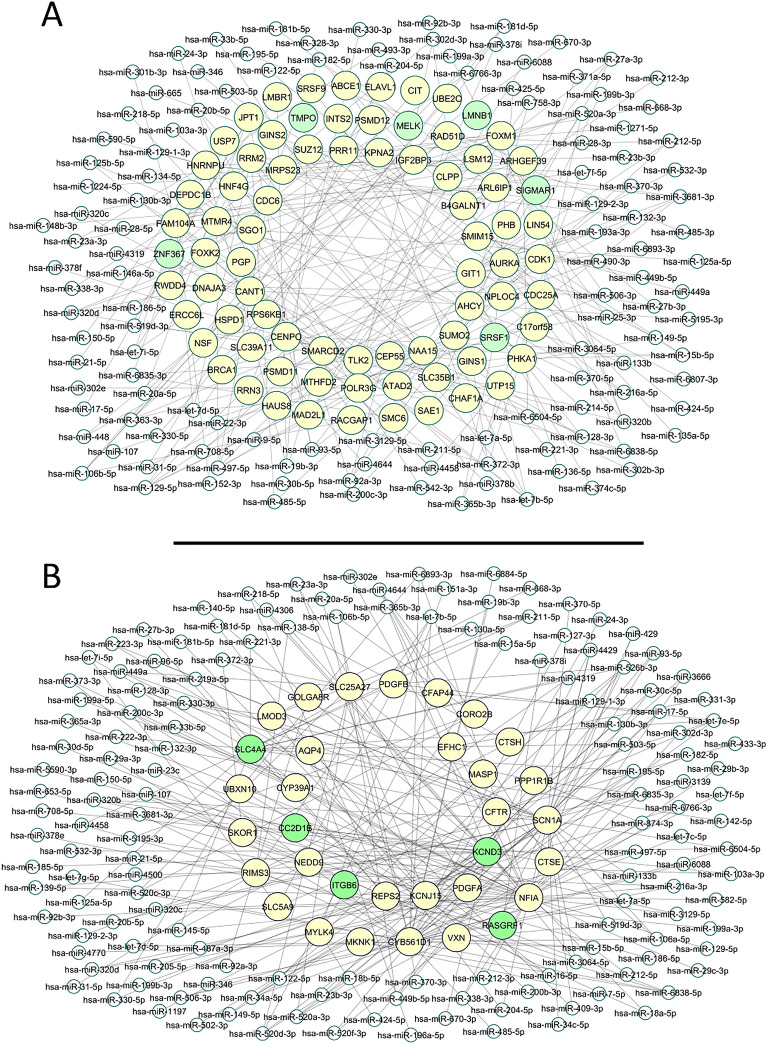
mRNA-miRNA interactions. (**A**) is the overexpressed group interaction, and (**B**) is the down-expressed group. The nodes represent genes, and miRNAs and edges represent interactions. The bigger nodes in the middle of the figure depict mRNAs, while the smaller ones are miRNAs. The light green nodes are survival-related mRNAs.

**Figure 8:**
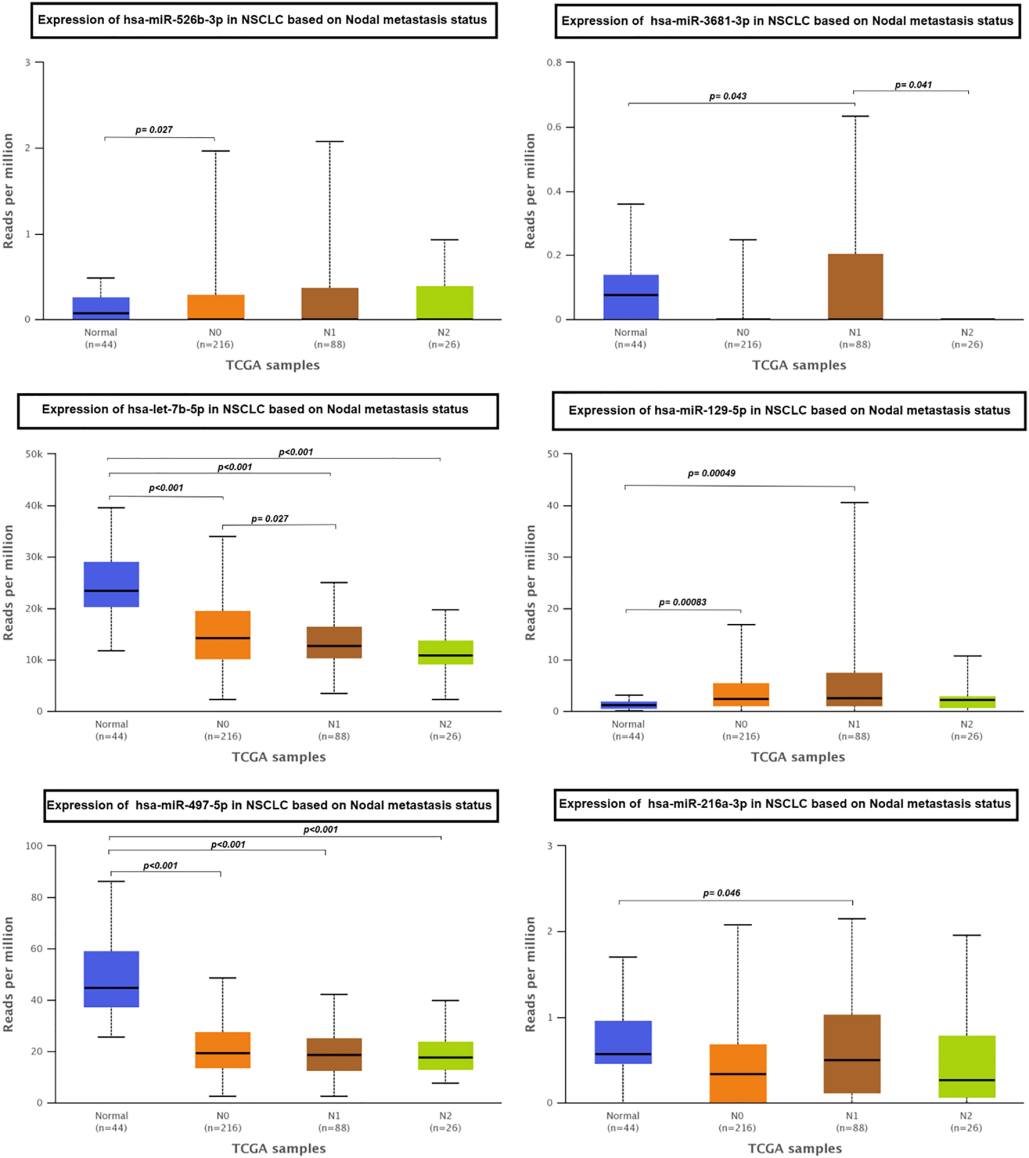
Expression levels of miRNA hub genes in normal and different stages of lymph node metastasis in LUSC (UALCAN).

Our results revealed that there are probably 526 miRNA interactions with candidate genes. Among downregulated mRNAs, *KCND3*, *SCN1A*, *NFIA*, and *CYB561D1* were targeted by the highest number of miRNAs. However, hsa-miR-526b-3p and hsa-let-7e-5p target the most genes in this group. On the other hand, among overexpressed mRNAs group, *TLK2*, *CDC25A*, *FAM104A*, and *HNRNPU* were targeted by more than 10 miRNAs. The number of miRNAs in this group is much more; among them, hsa-let-7b-5p, hsa-miR-129-5p, hsa-miR-3681-3p, hsa-miR-520d-3p, hsa-miR-497-5p, hsa-miR-216a-3p revealed the highest number of mRNAs target (Fig. 7A,B). *NEDD9* was the only gene among the drug resistance mRNAs that was targeted by 6 miRNAs. Then, expression levels of the miRNAs which were targeting more mRNAs including, hsa-miR-526b-3p, hsa-let-7e-5p, hsa-let-7b-5p, hsa-miR-129-5p, hsa-miR-3681-3p, hsa-miR-520d-3p, hsa-miR-497-5p, hsa-miR-216a-3p were analyzed by the UALCAN website. As shown in the Fig. 8, the miRNA expression of hsa-miR-526b-3p, hsa-let-7b-5p, hsa-miR-129-5p, hsa-miR-3681-3p, hsa-miR-497-5p, hsa-miR-216a-3p were differ significantly between the tumor tissues and normal (p value < 0.05). However, the expression of hsa-let-7e-5p and hsa-miR-520d-3p in tumor tissues were not significant.

### Hub genes selection

**Figure 9:**
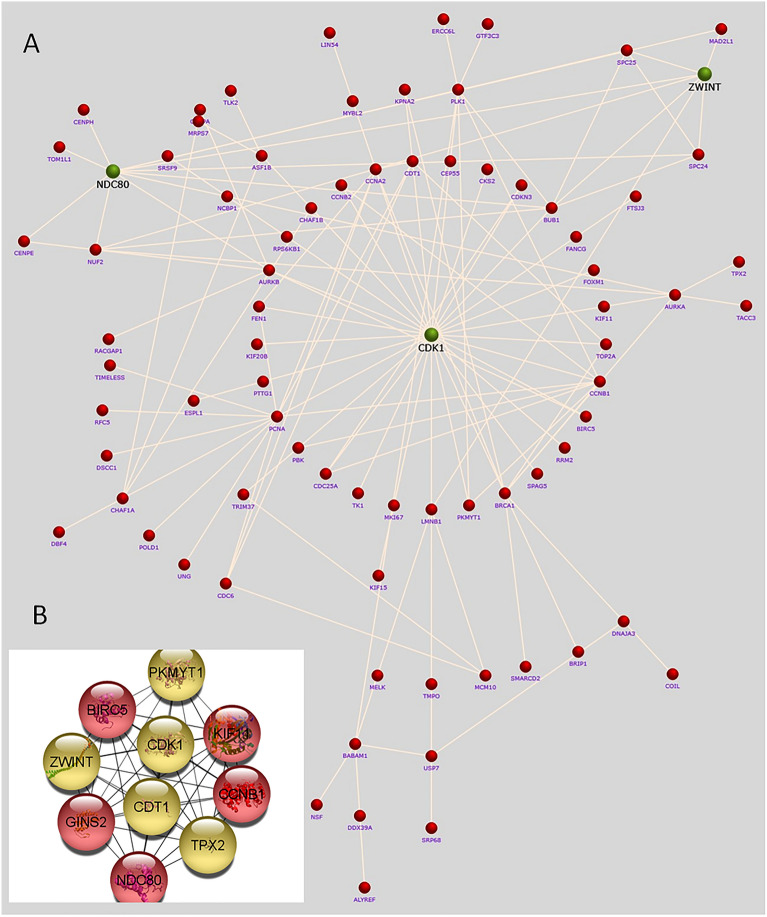
PPI network differentially expressed genes. (**A**) The interaction network of the hub genes and their related neighboring genes using the FunRich software (green nodes: CDK1, ZWINT and NDC80). (**B**) Top 10 hub genes in bottleneck method of cytoHubba.

**Figure 10:**
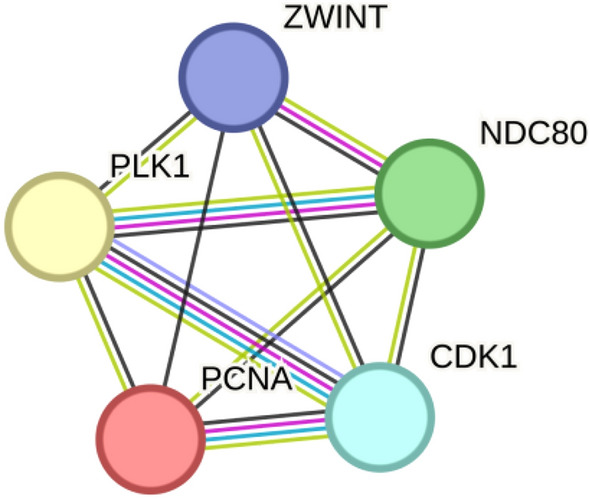
Protein–protein interaction network for hub genes.

The Protein–protein interaction (PPI) network of the 397 differentially expressed genes created by the FunRich software. *CDK1*, *PLK1*, *PCNA*, *ZWINT*, *NDC8* were the important nodes (genes) with many edges with at least 10 proteins (Fig. 9A), and all of which selected and ranked by at least one of the methods available in the cytoHubba, one of the Cytoscape plugin. CytoHubba provides a simple interface to analyze a network based on shortest paths by computing eleven topological analysis methods including Degree, Edge Percolated Component, Maximum Neighborhood Component, Density of Maximum Neighborhood Component, Maximal Clique Centrality and six centralities (Bottleneck, EcCentricity, Closeness, Radiality, Betweenness, and Stress) in one stop shopping way^25^. Among them, *ZWINT* and *NDC80* were identified in this study for the first time as our knowledge as hub genes for lymph node metastasis in LUSC. *CDK1* and *ZWINT* were as 10 top genes in more than four cytoHubba methods, but *NDC80* was only in the bottleneck cytoHubba method. Interestingly, *CDK1*, *ZWINT* and *NDC80* were commonly as 10 top genes in bottleneck method (Fig. 9B). In this work, a directed statistically significant reliable Protein–protein interaction network was constructed from the hub genes (Fig. 10). In the present study we displayed (Supplementary File 4) direct and indirect interactions to point out signaling pathways which involved in a lymph node metastasis upstream of hub genes.

## Discussion

Given the importance of lymph node metastasis in the process of metastasis in LUSC, in this study we compared the gene-expression profiles of patients with N + metastasis, without lymph node metastasis and normal tissue samples to find genes involved in lymph node metastasis encoded in the primary tumor.

Our results showed that a total of 281 and 116 genes were significantly up- and down- regulated, respectively, not only in the N0 and N + compared with normal but also in N + compared to N0. Among the 397 identified DE genes, 56 genes were significantly correlated with CD151, MMP1, PVT1, and SKP2 as reference genes for lymph node metastasis, and due to meeting best criteria in the ROC analysis were known be excellent for the distinguish lymph node metastasis cancer from primary LUSC without metastasis (N0). Takada M et al. in 2004^26^ achieved diagnostic genes which predicted lymph node metastasis in lung squamous cell carcinomas and lung adenocarcinomas patients, compared to our study, they used microarray data and lower samples. Importantly, we analyzed genes that were not only deregulated significantly in N + and N0 rather than normal, but also in N + compared to N0, which could have greater predictive value for metastatic status.

The results of functional enrichment analysis showed that the DEGs were related to pathways such as “surfactant metabolism pathway” (which has a role in the regulation of the cancer microenvironment and has been suggested as a target in cancer immunotherapy^27^),“Lung fibrosis” (Several studies have provided histopathological evidence of an increased incidence of lung cancer in pulmonary fibrosis^28^), “mitotic division,” and “cell cycle” which are consistent with DEGs role in LUSC lymph node metastasis.

In addition, in our study among the total differentially expressed genes, *SLC46A2*, *ZNF367*, *AC107214.1* and *NCBP1* were significantly associated with overall survival in multivariate Cox regression analyses. *SLC46A2* belongs to the solute carrier proteins (SLC) superfamily, which includes more than 400 transport proteins in 65 families and transport a wide variety of substances across the cell membrane and they also transport drugs across the lipid bilayer, so they play a role in carcinogenesis^29^. Consistent with our study, previously published article also described *SLC46A2* as a prognostic biomarker for lung squamous cell carcinomas and lung adenocarcinomas patients^30^. Interestingly, our results showed that the *SLC46A2* gene was significantly associated with metastasis and survival, indicating the importance of this gene in LUSC.

Zinc finger protein 367 (ZNF367) belongs to the zinc finger protein family and functions as a transcriptional factor with a unique zinc finger motif. Several studies have shown that this gene involved in overall survival and prognosis of breast cancer^31,32^. Nuclear cap-binding protein 1 (NCBP1) is require for capped RNA synthesis and is able to mediated proliferation, migration and invasion by participate in transcriptional and post-transcriptional processes and, it has been shown that *NCBP1* to be associated with survival in lung cancer patients^33,34^. According to our results and previous studies, *SLC46A2*, *ZNF367* and *NCBP* genes could be introduced as important factors for survival and clinical management in LUSC. In this study, for the first time to our knowledge, we report *ZNF367* and *AC107214.1* genes in LUSC as an important factor in survival.

In addition, we have identified *NEDD9*, *MRPL21*, *SNRPF*, and *SCLT1* genes that can predict the response of LUSC patients to chemotherapy. Previous studies have shown that *NEDD9* plays a key role in tumor initiation, progression, and metastasis^35^, and Kondo et al. found that *NEDD9* could confer resistance to the chemotherapeutic agents in lung cancer 293 T, A549, PC-9 and PC-14 cell lines^36^. The mitochondrial proteins which are important for various mitochondrial functions, such as ribosomal subunit proteins (MRPL21), may contribute to drug resistance, for example, Huang et al. in 2020 showed that increased levels of MRPL21 is responsible for treatment strategies to reduce the chemotherapeutic resistance to recurrent acute myeloid leukemia^37,38^. It has been reported that *SCLT1* by inducing apoptosis combined with chemotherapy drugs enhanced cancer cell death. SCLT1- induced apoptosis might be related to DNA damage response^39^ and Liu et al. have shown *SCLT1* to be useful biomarker to predict response to chemotherapy in patients with hepatocellular carcinoma^39^. *SNRPF* encoded spliceosome small nuclear ribonucleoproteins. *SNRPF* dysregulation has been reported in some cancers, including colorectal, laryngeal squamous cell carcinoma cells and renal cell carcinoma, but not in lung cancer^40^. Therefore, the *SNRPF*, *SCLT1* and *MRPL21* genes presented in this article are considered as novel biomarkers for predicting the response of LUSC patients to chemotherapy, as they have not been published before.

Further, thorough the analysis of mRNA-miRNA interaction networks, we found DEGs which were targeted by more miRNAs, including *KCND3*, *SCN1A*, *NFIA*, *CYB561D1, TLK2*, *CDC25A*, *FAM104A*, and *HNRNPU.* As a member of the nuclear factor I family, *NFIA* can lead to uncontrolled cell proliferation and tumor initiation and progression and has been reported by Zhao et al. In 2017 in patients with squamous cell cancer, adenocarcinoma and large cell carcinoma^41^. So far, there have been no reports on other three down regulated mRNAs (*KCND3*, *SCN1A* and *CYB561D1*) in lung cancer targeted by miRNAs. Among up regulated mRNAs found in this study, the association of mutant *TLK2* (A member of Tousled-like kinases family) has been reported in breast cancer^42^. However, there are no reports on the function of *TLK2* in the context of lung cancer. Overexpression of cell division cycle 25A (CDC25A), a member of Cdc25 family, has also been reported as a poor prognosis marker in NSCLC. Li et al. in 2020 has been reported that miR-365 target CDC25A mRNA and reduce the expression of CDC25A in lung cancer^43^. Interestingly, so far, there are no report in the literature on the *FAM104A* and *HNRNPU*.

Moreover, we found various miRNAs including hsa-miR-526b-3p and hsa-let-7e-5p, hsa-let-7b-5p, hsa-miR-129-5p, hsa-miR-3681-3p, hsa-miR-520d-3p, hsa-miR-497-5p and hsa-miR-216a-3p could target the most candidate differentially expressed genes. Interestingly, some of these miRNAs such as hsa-miR-526b-3p was also detected as hub miRNAs associated with colorectal cancer progression by Motieghader et al.^44^. Cell migration mediated by let-7e-5p has been confirmed in the colon carcinoma. Although its underlying mechanism is unclear, modulation of MYC pathways assumed in this study^45^. Gharib et al. suggested that miR-497-5p overexpression affect the development of colorectal cancer by regulating cell proliferation. Therefore, miR-497-5p upregulation could be considered as a potential therapeutic target^46^. The functional molecular of hsa-let-7b-5p, hsa-miR-129-5p, hsa-miR-3681-3p, hsa-miR-520d-3p, hsa-miR-216a-3p and their targets in lymph node metastasis have not been reported previously. Also, the miRNA expression of hsa-miR-526b-3p, hsa-let-7b-5p, hsa-miR-129-5p, hsa-miR-3681-3p, hsa-miR-497-5p, hsa-miR-216a-3p were found differ significantly between the tumor tissues and normal in further analysis.

Generally speaking, identified miRNAs and target mRNAs show various expression patterns that are proving to be clinically relevant to LUSC's lymph node metastasis. Such observations indicate that a subgroup of miRNAs play an important role in lung squamous cell carcinoma lymphatic progression.

Finally, CDK1, PLK1, PCNA, ZWINT and NDC80 identified as hub genes for underlying molecular mechanisms of lung squamous cell carcinoma lymphatic metastasis. Among them, *CDK1*, *ZWINT* and *NDC80* genes were identified for the first time to our knowledge in lymph node metastasis of LUSC. The ZW10 interacting kinetochore protein (*ZWINT*), encodes a protein involved in kinetochore function during mitotic cycle. Overexpression of *ZWINT* often resulted in abnormal mitosis in human cancers, which is a common feature of most malignancies. In addition, nuclear division cycle 80 (NDC80) is another mitotic regulator highly expressed in various human malignancies. *NDC80*, participate in regulation of mitosis by spindle assembly checkpoint^47^. NDC80 may interact with a kinase NEK2 and one of the centrosome proteins CEP250 to play a role in lymph node metastasis in cancers^48,49^.

Cyclin dependent kinase 1 (CDK1) as a serine/threonine kinase, regulate the cell cycle by promoting the G2/M as well as G1/S transitions. Alteration in CDK1 activity due to upregulation of CDK1 is closely related to cell proliferation^50^. In line with our results, CDK1 was one of the 20 key hub genes related to pancreatic cancer metastasis and prognosis. CDK1 and MYC can promote the formation of metastasis niches by regulating the activity of CD4 + T cells^51^. Also, Chen et al. found that CDK1 promotes the EMT and migration of head and neck squamous cell carcinomas (HNSCCs) cells by inhibiting ∆Np63α^52^. In addition, CDK1 can bind to FGFR1, which leads to cell proliferation, invasion and migration and affects lymph node metastasis^48,53^.

PCNA plays an important role in DNA replication, but is also associated with other functions such as chromatin remodeling, DNA repair, sister-chromatid cohesion and cell cycle control^54,55^. It has been shown taht PCNA was significantly related to lymph node metastasis in gastric cancer^56^. Interestingly, a preveoius study has shwon that PCNA as an oncogene can be involved in NSCLC progression through up-regulation of STAT3^57^.

Polo-like kinase 1 (PLK1) is a highly conserved serine/threonine kinase with important roles in mitosis and cell cycle regulation^58^. PLK1 is highly expressed in multiple tumors and promotes tumor cell proliferation and cell transformation, and is associated with clinical stages and invasion^59^. Previous studies have shown that PLK1 can play a role invasion and metastasis through beta-1 integrin by vimentin phosphorylation in breast cancer^60^ or through CD44v6, matrix metalloproteinase (MMP)-2, and MMP-9 in thyroid cancer^61^. Interestingly, consistent with our results, in NSCLC, active PLK1 has been shown to upregulate the levels of p-Smad2, a TGF-β effector, leading to the promote metastasis and invasion thorough upregulates TNFAIP6^62^. Thus, it is reasonable that deregulation of identified hub genes is associated not only with tumorigenesis but also with lymph node metastasis.

Generally speaking, in the present work ROC curve analysis was used to evaluate diagnostic ability of DEGs which could be useful at the time of diagnosis of the initial stage in determining the course of a LUSC lesion (from tumor promotion, malignant conversion to lymphatic metastasis). The prognostic importance of DEGs is also assessed that provides information on the likely patient health outcome. Since the decision about treatment choice in the early-stage of cancer may be difficult for clinicians, LUSC drug resistance/sensitivity associated DEGs were also confirmed. Simply put, we performed a series of analyses to ensure whether our new expression profile (including, differentially expressed genes with significant differences not only between N + vs. normal and N0 vs. normal but also between N + vs. N0), could be functional at different phases of patient management. Besides, five identified hub genes were herald as underpin molecular mechanisms of lung squamous cell carcinoma lymphatic metastasis. Although, in the present study lymph node metastasis has been speculated as a result of disruption of normal regulation of the cell cycle caused by deregulation of these hub genes, it needs to be credible.

## Conclusion

In summary, this study, by identifying lymph node metastasis predicting biomarkers and improving understanding of the less well-known genes of LUSC, hopes to address problems with poor prognosis of this subset of patients due to delayed diagnosis. The results of the present study should be interpreted with caution because only bioinformatics techniques were used to identify biomarkers, and the results were not confirmed through in vitro study.

## Supplementary Information

Supplementary Information 1.

Supplementary Information 2.

Supplementary Information 3.

Supplementary Information 4.

Supplementary Table 1.

Supplementary Table 2.

## Data Availability

Dataset analyzed during the current study (TCGA-LUSC dataset) were previously generated and are available from LinkedOmics (http://linkedomics.org/). Also, this study includes research data from UALCAN database (http://ualcan.path.uab.edu) available in web link.

## Abbreviations

LUSC: Lung squamous cell carcinoma
NSCLC: Non-small cell lung cancer
LNM: Lymph node metastasis
miRNA: Micro RNA
TCGA: The cancer genome atlas
CPM: Counts per million
TMM: Trimmed mean of the M-values
DGE: Differential gene expression analysis
GO: Gene ontology
CC: Cellular component
MF: Molecular function
BP: Biological process
KEGG: Kyoto encyclopedia of genes and genomes
CC: Correlation coefficients
ROC: Receiver operating characteristic
CCLE: Cancer cell line encyclopedia
FDA: Food and drug administration
HPRD: Human protein reference database
FDR: False discovery rate
AUC: Area under curve
PPI network: Protein–protein interaction network
SLC: Solute carrier proteins
ZNF367: Zinc finger protein 367
NCBP1: Nuclear cap-binding protein 1
CDC25A: Cell division cycle 25A
ZWINT: ZW10 interacting kinetochore protein
NDC80: Nuclear division cycle 80
CDK1: Cyclin dependent kinase 1
