# Supplementary material for: Genes whose expressions in the primary lung squamous cell carcinoma are able to accurately predict the progression of metastasis through lymphatic system, inferred from a bioinformatics analyses
Source: Sci Rep. 2023 Apr 25;13:6733. doi: 10.1038/s41598-023-33897-3 (PMC10130036; doi:10.1038/s41598-023-33897-3)
Supplement: Supplementary file 3 — Supplementary Information 3. [file 41598_2023_33897_MOESM3_ESM.docx]

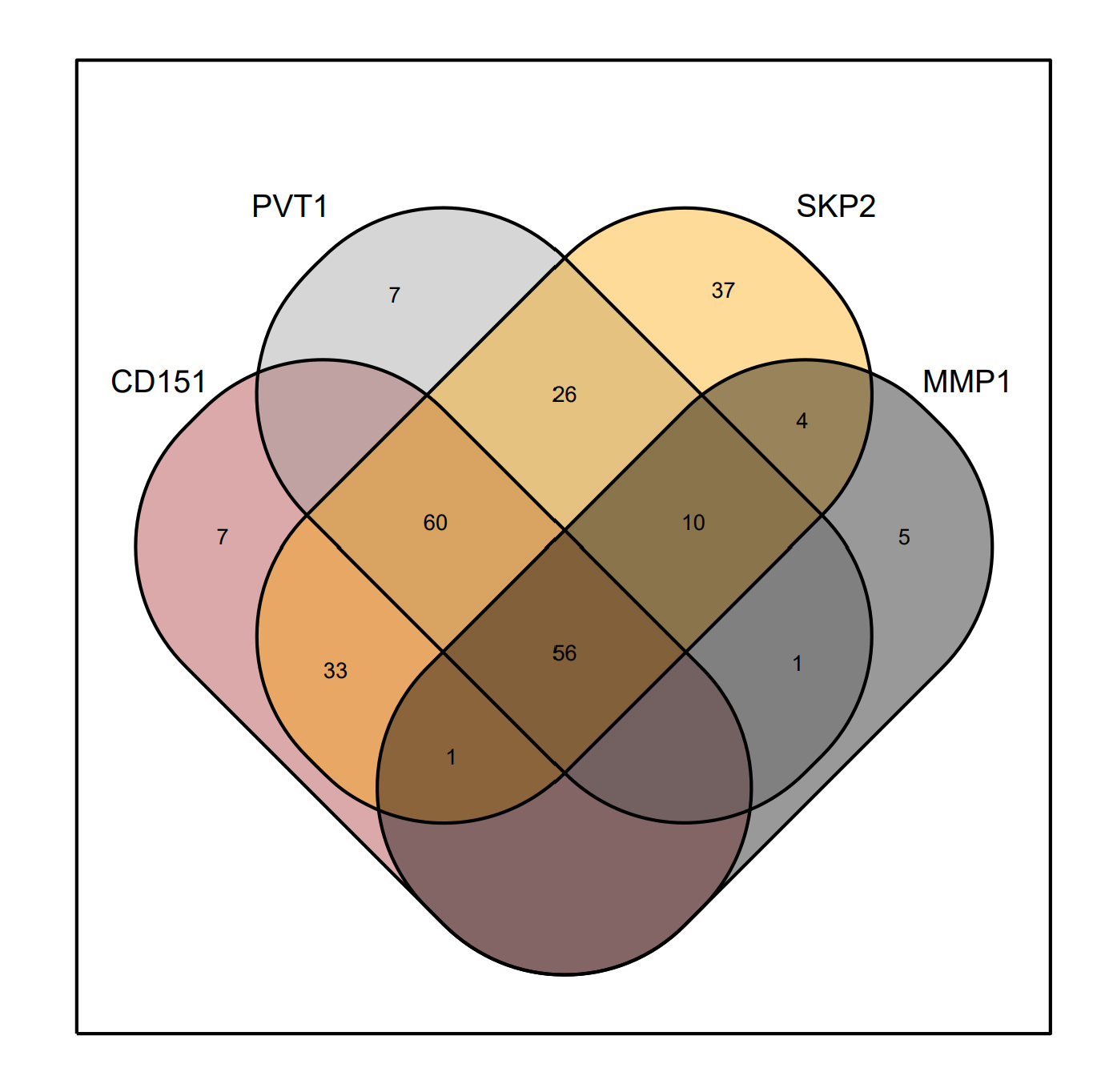


Supplementary figure legend. Venn diagram of the significant correlated DEGs with the reference genes

**References:**

**MMP1:**

| 1- | Wang Y, Ding X, Liu B, Li M, Chang Y, Shen H, Xie SM, Xing L, Li Y. ETV4 overexpression promotes progression of non-small cell lung cancer by upregulating PXN and MMP1 transcriptionally. Mol Carcinog. 2020 Jan;59(1):73-86. doi: 10.1002/mc.23130. |
| --- | --- |
| 2- | Liu Y, Lin D, Xiao T, Ma Y, Hu Z, Zheng H, Zheng S, Liu Y, Li M, Li L, Cao Y, Guo S, Han N, Di X, Zhang K, Cheng S, Gao Y. An immunohistochemical analysis-based decision tree model for estimating the risk of lymphatic metastasis in pN0 squamous cell carcinomas of the lung. Histopathology. 2011 Nov;59(5):882-91. doi: 10.1111/j.1365-2559.2011.04013.x. |
| 3- | Gouyer V, Conti M, Devos P, Zerimech F, Copin MC, Créme E, Wurtz A, Porte H, Huet G. Tissue inhibitor of metalloproteinase 1 is an independent predictor of prognosis in patients with nonsmall cell lung carcinoma who undergo resection with curative intent. Cancer. 2005 Apr 15;103(8):1676-84. doi: 10.1002/cncr.20965. |
| 4- | Fang S, Jin X, Wang R, Li Y, Guo W, Wang N, Wang Y, Wen D, Wei L, Zhang J. Polymorphisms in the MMP1 and MMP3 promoter and non-small cell lung carcinoma in North China. Carcinogenesis. 2005 Feb;26(2):481-6. doi: 10.1093/carcin/bgh327. |
| 5- | Lin TS, Chiou SH, Wang LS, Huang HH, Chiang SF, Shih AY, Chen YL, Chen CY, Hsu CP, Hsu NY, Chou MC, Kuo SJ, Chow KC. Expression spectra of matrix metalloproteinases in metastatic non-small cell lung cancer. Oncol Rep. 2004 Oct;12(4):717-23. |
| 6- | Peng ZM, Yang ZL, Liu YW. Expression of MMP1 and TIMP1 proteins in lung cancer and its biological significance. Hunan Yi Ke Da Xue Xue Bao. 2002 Apr 28;27(2):159-61. Chinese. |
| 7- | Wang L, He J, Hu H, Tu L, Sun Z, Liu Y, Luo F. Lung CSC-derived exosomal miR-210-3p contributes to a pro-metastatic phenotype in lung cancer by targeting FGFRL1. J Cell Mol Med. 2020 Jun;24(11):6324-6339. doi: 10.1111/jcmm.15274. |
| 8- | Guo J, Hu Y, Jin G, Zhao Z, Nan F, Hu X, Hu Y, Han Q. Wogonin Restrains the Malignant Progression of Lung Cancer through Modulating MMP1 and PI3K/AKT Signaling Pathway. Protein Pept Lett. 2022 Oct 27. doi: 10.2174/0929866530666221027152204. |
| 9- | Kuhn H, Frille A, Petersen MA, Oberhuber-Kurth J, Hofmann L, Gläser A, Taubenheim S, Klagges S, Kraemer S, Broschewitz J, von Laffert M, Wirtz H. IGFBP3 inhibits tumor growth and invasion of lung cancer cells and is associated with improved survival in lung cancer patients. Transl Oncol. 2022 Oct 17;27:101566. doi: 10.1016/j.tranon.2022.101566. |
| 10- | Zhang D, Zhang T, Zhang Y, Li Z, Li H, Zhang Y, Liu C, Han Z, Li J, Zhu J. Screening the components of Saussurea involucrata for novel targets for the treatment of NSCLC using network pharmacology. BMC Complement Med Ther. 2022 Feb 28;22(1):53. doi: 10.1186/s12906-021-03501-0. |
| 11- | Yao Y, Li Z, Gao W. Identification of Hub Genes in Idiopathic Pulmonary Fibrosis and NSCLC Progression:Evidence From Bioinformatics Analysis. Front Genet. 2022 Apr 11;13:855789. doi: 10.3389/fgene.2022.855789. |
| 12- | Gabasa M, Radisky ES, Ikemori R, Bertolini G, Arshakyan M, Hockla A, Duch P, Rondinone O, Llorente A, Maqueda M, Davalos A, Gavilán E, Perera A, Ramírez J, Gascón P, Reguart N, Roz L, Radisky DC, Alcaraz J. MMP1 drives tumor progression in large cell carcinoma of the lung through fibroblast senescence. Cancer Lett. 2021 Jun 1;507:1-12. doi: 10.1016/j.canlet.2021.01.028. |
| 13- | Li Y, Huang H, Ye X, Huang Z, Chen X, Wu F, Lin T. miR-202-3p negatively regulates MMP-1 to inhibit the proliferation, migration and invasion of lung adenocarcinoma cells. Cell Cycle. 2021 Feb;20(4):406-416. doi: 10.1080/15384101.2021.1876390. |
| 14- | Shen J, Han L, Xue Y, Li C, Jia H, Zhu K. Ropivacaine Inhibits Lung Cancer Cell Malignancy Through Downregulation of Cellular Signaling Including HIF-1α In Vitro. Front Pharmacol. 2022 Feb 23;12:806954. doi: 10.3389/fphar.2021.806954. |
| 15- | Jeon S, Kim HK, Kwon JY, Baek SH, Ri HS, Choi HJ, Cho HR, Lee YS, Kim JY, Kim J, Bae J, Lee HJ. Role of Sevoflurane on Natural Killer Group 2, Member D-Mediated Immune Response in Non-Small-Cell Lung Cancer: An In Vitro Study. Med Sci Monit. 2020 Nov 3;26:e926395. doi: 10.12659/MSM.926395. |
| 16- | Yang YF, Chang YC, Jan YH, Yang CJ, Huang MS, Hsiao M. Squalene synthase promotes the invasion of lung cancer cells via the osteopontin/ERK pathway. Oncogenesis. 2020 Aug 29;9(8):78. doi: 10.1038/s41389-020-00262-2. |
| 17- | Niu Y, Tang D, Fan L, Gao W, Lin H. CCL25 promotes the migration and invasion of non-small cell lung cancer cells by regulating VEGF and MMPs in a CCR9-dependent manner. Exp Ther Med. 2020 Jun;19(6):3571-3580. doi: 10.3892/etm.2020.8635. |
| 18- | Son J, Lee SY. Ursonic acid exerts inhibitory effects on matrix metalloproteinases via ERK signaling pathway. Chem Biol Interact. 2020 Jan 5;315:108910. doi: 10.1016/j.cbi.2019.108910. |
| 19- | Naghizadeh S, Mansoori B, Mohammadi A, Kafil HS, Mousavi Z, Sakhinia E, Baradaran B. Effects of HMGA2 gene downregulation by siRNA on lung carcinoma cell migration in A549 cell lines. J Cell Biochem. 2019 Apr;120(4):5024-5032. doi: 10.1002/jcb.27778. |
| 20- | Hsu CY, Chang GC, Chen YJ, Hsu YC, Hsiao YJ, Su KY, Chen HY, Lin CY, Chen JS, Chen YJ, Hong QS, Ku WH, Wu CY, Ho BC, Chiang CC, Yang PC, Yu SL. FAM198B Is Associated with Prolonged Survival and Inhibits Metastasis in Lung Adenocarcinoma via Blockage of ERK-Mediated MMP-1 Expression. Clin Cancer Res. 2018 Feb 15;24(4):916-926. doi: 10.1158/1078-0432.CCR-17-1347. |
| 21- | Huang Q, Wei H, Wu Z, Li L, Yao L, Sun Z, Li L, Lin Z, Xu W, Han S, Cao W, Xu Y, Song D, Yang X, Xiao J. Preferentially Expressed Antigen of Melanoma Prevents Lung Cancer Metastasis. PLoS One. 2016 Jul 8;11(7):e0149640. doi: 10.1371/journal.pone.0149640. |
| 22- | Liu XQ, Kiefl R, Roskopf C, Tian F, Huber RM. Interactions among Lung Cancer Cells, Fibroblasts, and Macrophages in 3D Co-Cultures and the Impact on MMP-1 and VEGF Expression. PLoS One. 2016 May 27;11(5):e0156268. doi: 10.1371/journal.pone.0156268. |
| 23- | The VEGFR2, COX-2 and MMP-2 polymorphisms are associated with clinical outcome of patients with inoperable non-small cell lung cancer |
| 24- | Bi HX, Shi HB, Zhang T, Cui G. PRDM14 promotes the migration of human non-small cell lung cancer through extracellular matrix degradation in vitro. Chin Med J (Engl). 2015 Feb 5;128(3):373-7. doi: 10.4103/0366-6999.150109. |
| 25- | Jandova J, Mason CJ, Pawar SC, Watts GS. Fn14 receptor promotes invasive potential and metastatic capacity of non-small lung adenocarcinoma cells through the up-regulation of integrin α6. Neoplasma. 2015;62(1):41-52. doi: 10.4149/neo_2015_006. |
| 26- | Yang YF, Jan YH, Liu YP, Yang CJ, Su CY, Chang YC, Lai TC, Chiou J, Tsai HY, Lu J, Shen CN, Shew JY, Lu PJ, Lin YF, Huang MS, Hsiao M. Squalene synthase induces tumor necrosis factor receptor 1 enrichment in lipid rafts to promote lung cancer metastasis. Am J Respir Crit Care Med. 2014 Sep 15;190(6):675-87. doi: 10.1164/rccm.201404-0714OC. |
| 27- | Li X, Tai HH. Thromboxane A2 receptor-mediated release of matrix metalloproteinase-1 (MMP-1) induces expression of monocyte chemoattractant protein-1 (MCP-1) by activation of protease-activated receptor 2 (PAR2) in A549 human lung adenocarcinoma cells. Mol Carcinog. 2014 Aug;53(8):659-66. doi: 10.1002/mc.22020. |
| 28- | Chiang YY, Chow KC, Lin TY, Chiang IP, Fang HY. Hepatocyte growth factor and HER2/neu downregulate expression of apoptosis-inducing factor in non-small cell lung cancer. Oncol Rep. 2014 Feb;31(2):597-604. doi: 10.3892/or.2013.2867. |
| 29- | Kim B, Sohn EJ, Jung JH, Shin EA, You OH, Im J, Kim SH. Inhibition of ZNF746 suppresses invasion and epithelial to mesenchymal transition in H460 non-small cell lung cancer cells. Oncol Rep. 2014 Jan;31(1):73-8. doi: 10.3892/or.2013.2801. |
| 30- | Masuzawa M, Mikami T, Numata Y, Tokuyama W, Masuzawa M, Murakumo Y, Okayasu I, Katsuoka K. Association of D2-40 and MMP-1 expression with cyst formation in lung metastatic lesions of cutaneous angiosarcoma on the scalp: immunohistochemical analysis of 23 autopsy cases. Hum Pathol. 2013 Dec;44(12):2751-9. doi: 10.1016/j.humpath.2013.07.022. |
| 31- | Fanjul-Fernández M, Folgueras AR, Fueyo A, Balbín M, Suárez MF, Fernández-García MS, Shapiro SD, Freije JMP, López-Otín C. Matrix metalloproteinase Mmp-1a is dispensable for normal growth and fertility in mice and promotes lung cancer progression by modulating inflammatory responses. J Biol Chem. 2013 May 17;288(20):14647-14656. doi: 10.1074/jbc.M112.439893. Epub 2013 Apr 2. Erratum in: J Biol Chem. 2018 Jul 27;293(30):11970. |
| 32- | Husmann K, Arlt MJ, Muff R, Langsam B, Bertz J, Born W, Fuchs B. Matrix Metalloproteinase 1 promotes tumor formation and lung metastasis in an intratibial injection osteosarcoma mouse model. Biochim Biophys Acta. 2013 Feb;1832(2):347-54. doi: 10.1016/j.bbadis.2012.11.006. |
| 33- | Lavergne M, Jourdan ML, Blechet C, Guyetant S, Pape AL, Heuze-Vourc'h N, Courty Y, Lerondel S, Sobilo J, Iochmann S, Reverdiau P. Beneficial role of overexpression of TFPI-2 on tumour progression in human small cell lung cancer. FEBS Open Bio. 2013 Jun 27;3:291-301. doi: 10.1016/j.fob.2013.06.004. |
| 34- | Hida Y, Hamada J. Differential expressions of matrix metalloproteinases, a disintegrin and metalloproteinases, and a disintegrin and metalloproteinases with thrombospondin motifs and their endogenous inhibitors among histologic subtypes of lung cancers. Anticancer Agents Med Chem. 2012 Sep;12(7):744-52. doi: 10.2174/187152012802650156. |
| 35- | Wei S, Hao C, Li X, Zhao H, Chen J, Zhou Q. Effects of BTG2 on proliferation inhibition and anti-invasion in human lung cancer cells. Tumour Biol. 2012 Aug;33(4):1223-30. doi: 10.1007/s13277-012-0370-y. |
| 36- | Foley CJ, Luo C, O'Callaghan K, Hinds PW, Covic L, Kuliopulos A. Matrix metalloprotease-1a promotes tumorigenesis and metastasis. J Biol Chem. 2012 Jul 13;287(29):24330-8. doi: 10.1074/jbc.M112.356303. |
| 37- | Li X, Tai HH. Increased expression of matrix metalloproteinases mediates thromboxane A2-induced invasion in lung cancer cells. Curr Cancer Drug Targets. 2012 Jul;12(6):703-15. doi: 10.2174/156800912801784884. |
| 38- | Liu L, Wu J, Wu C, Wang Y, Zhong R, Zhang X, Tan W, Nie S, Miao X, Lin D. A functional polymorphism (-1607 1G→2G) in the matrix metalloproteinase-1 promoter is associated with development and progression of lung cancer. Cancer. 2011 Nov 15;117(22):5172-81. doi: 10.1002/cncr.26154. |
| 39- | Liu Y, Lin D, Xiao T, Ma Y, Hu Z, Zheng H, Zheng S, Liu Y, Li M, Li L, Cao Y, Guo S, Han N, Di X, Zhang K, Cheng S, Gao Y. An immunohistochemical analysis-based decision tree model for estimating the risk of lymphatic metastasis in pN0 squamous cell carcinomas of the lung. Histopathology. 2011 Nov;59(5):882-91. doi: 10.1111/j.1365-2559.2011.04013.x. |
| 40- | Man S, Gao W, Zhang Y, Liu Z, Yan L, Huang L, Liu C. Formosanin C-inhibited pulmonary metastasis through repression of matrix metalloproteinases on mouse lung adenocarcinoma. Cancer Biol Ther. 2011 Mar 15;11(6):592-8. doi: 10.4161/cbt.11.6.14668. |
| 41- | Gaud G, Iochmann S, Guillon-Munos A, Brillet B, Petiot S, Seigneuret F, Touzé A, Heuzé-Vourc'h N, Courty Y, Lerondel S, Gruel Y, Reverdiau P. TFPI-2 silencing increases tumour progression and promotes metalloproteinase 1 and 3 induction through tumour-stromal cell interactions. J Cell Mol Med. 2011 Feb;15(2):196-208. doi: 10.1111/j.1582-4934.2009.00989.x. |
| 42- | Wu YH, Wu TC, Liao JW, Yeh KT, Chen CY, Lee H. p53 dysfunction by xeroderma pigmentosum group C defects enhance lung adenocarcinoma metastasis via increased MMP1 expression. Cancer Res. 2010 Dec 15;70(24):10422-32. doi: 10.1158/0008-5472.CAN-10-2615. |
| 43- | Iochmann S, Bléchet C, Chabot V, Saulnier A, Amini A, Gaud G, Gruel Y, Reverdiau P. Transient RNA silencing of tissue factor pathway inhibitor-2 modulates lung cancer cell invasion. Clin Exp Metastasis. 2009;26(5):457-67. doi: 10.1007/s10585-009-9245-z. |
| 44- | Ma W, Chen J, Xue X, Wang Z, Liu H, Wang T, Bai Y, Tang SC, Zhou Q. Alteration in gene expression profile and biological behavior in human lung cancer cell line NL9980 by nm23-H1 gene silencing. Biochem Biophys Res Commun. 2008 Jul 4;371(3):425-30. doi: 10.1016/j.bbrc.2008.04.083. |
| 45- | Sauter W, Rosenberger A, Beckmann L, Kropp S, Mittelstrass K, Timofeeva M, Wölke G, Steinwachs A, Scheiner D, Meese E, Sybrecht G, Kronenberg F, Dienemann H; LUCY-Consortium, Chang-Claude J, Illig T, Wichmann HE, Bickeböller H, Risch A. Matrix metalloproteinase 1 (MMP1) is associated with early-onset lung cancer. Cancer Epidemiol Biomarkers Prev. 2008 May;17(5):1127-35. doi: 10.1158/1055-9965.EPI-07-2840. |
| 46- | Sun T, Gao Y, Tan W, Ma S, Zhang X, Wang Y, Zhang Q, Guo Y, Zhao D, Zeng C, Lin D. Haplotypes in matrix metalloproteinase gene cluster on chromosome 11q22 contribute to the risk of lung cancer development and progression. Clin Cancer Res. 2006 Dec 1;12(23):7009-17. doi: 10.1158/1078-0432.CCR-06-0464. |
| 47- | Zhang C, Chakravarty D, Sakabe I, Mewani RR, Boudreau HE, Kumar D, Ahmad I, Kasid UN. Role of SCC-S2 in experimental metastasis and modulation of VEGFR-2, MMP-1, and MMP-9 expression. Mol Ther. 2006 May;13(5):947-55. doi: 10.1016/j.ymthe.2005.11.020. |
| 48- | Hofmann HS, Hansen G, Richter G, Taege C, Simm A, Silber RE, Burdach S. Matrix metalloproteinase-12 expression correlates with local recurrence and metastatic disease in non-small cell lung cancer patients. Clin Cancer Res. 2005 Feb 1;11(3):1086-92. |
| 49- | Wang X, Yang J, Ma Y, Zheng H, Cheng B, Zheng H, Zhu C. [Expression and significance of matrix metalloproteinases and their tissue inhibitors in the infiltration and metastasis of lung cancer]. Zhongguo Fei Ai Za Zhi. 2003 Aug 20;6(4):278-82. Chinese. doi: 10.3779/j.issn.1009-3419.2003.04.09. |
| 50- | Soon LL, Yie TA, Shvarts A, Levine AJ, Su F, Tchou-Wong KM. Overexpression of WISP-1 down-regulated motility and invasion of lung cancer cells through inhibition of Rac activation. J Biol Chem. 2003 Mar 28;278(13):11465-70. doi: 10.1074/jbc.M210945200. |
| 51- | Naglich JG, Jure-Kunkel M, Gupta E, Fargnoli J, Henderson AJ, Lewin AC, Talbott R, Baxter A, Bird J, Savopoulos R, Wills R, Kramer RA, Trail PA. Inhibition of angiogenesis and metastasis in two murine models by the matrix metalloproteinase inhibitor, BMS-275291. Cancer Res. 2001 Dec 1;61(23):8480-5. |
| 52- | Reichenberger F, Eickelberg O, Wyser C, Perruchoud AP, Roth M, Tamm M. Distinct endobronchial expression of matrix-metalloproteinases (MMP) and their endogenous inhibitors in lung cancer. Swiss Med Wkly. 2001 May 19;131(19-20):273-9. |
| 53- | Pritchard SC, Nicolson MC, Lloret C, McKay JA, Ross VG, Kerr KM, Murray GI, McLeod HL. Expression of matrix metalloproteinases 1, 2, 9 and their tissue inhibitors in stage II non-small cell lung cancer: implications for MMP inhibition therapy. Oncol Rep. 2001 Mar-Apr;8(2):421-4. |
| 54- | Thomas P, Khokha R, Shepherd FA, Feld R, Tsao MS. Differential expression of matrix metalloproteinases and their inhibitors in non-small cell lung cancer. J Pathol. 2000 Feb;190(2):150-6. doi: 10.1002/(SICI)1096-9896(200002)190:2<150::AID-PATH510>3.0.CO;2-W. |
| 55- | Merchant N, Nagaraju GP, Rajitha B, Lammata S, Jella KK, Buchwald ZS, Lakka SS, Ali AN. Matrix metalloproteinases: their functional role in lung cancer. Carcinogenesis. 2017 Aug 1;38(8):766-780. doi: 10.1093/carcin/bgx063. |
| 56- | MicroRNA-143 act as a tumor suppressor microRNA in human lung cancer cells by inhibiting cell proliferation, invasion, and migration |
| 57- | Xia Y, Zha J, Curull V, Sánchez-Font A, Guitart M, Rodríguez-Fuster A, Aguiló R, Barreiro E. Gene expression profile of epithelial-mesenchymal transition in tumours of patients with nonsmall cell lung cancer: the influence of COPD. ERJ Open Res. 2022 Jul 18;8(3):00105-2022. doi: 10.1183/23120541.00105-2022. |
| 58- | Nimma R, Kalvala AK, Patel N, Surapaneni SK, Sun L, Singh R, Nottingham E, Bagde A, Kommineni N, Arthur P, Nathani A, Meckes DG Jr, Singh M. Combined Transcriptomic and Proteomic Profiling to Unravel Osimertinib, CARP-1 Functional Mimetic (CFM 4.17) Formulation and Telmisartan Combo Treatment in NSCLC Tumor Xenografts. Pharmaceutics. 2022 May 28;14(6):1156. doi: 10.3390/pharmaceutics14061156. |
| 59- | Liu B, Li J, Li JM, Liu GY, Wang YS. HOXC-AS2 mediates the proliferation, apoptosis, and migration of non-small cell lung cancer by combining with HOXC13 gene. Cell Cycle. 2021 Jan;20(2):236-246. doi: 10.1080/15384101.2020.1868161. |
| 60- | Li X, Li S, Pang J, Huang F, Guo B, Liu H. [Alkaline processing of cantharidin can significanty improve the antitumor activity of cantharidin]. Nan Fang Yi Ke Da Xue Xue Bao. 2020 Sep 30;40(9):1332-1339. Chinese. doi: 10.12122/j.issn.1673-4254.2020.09.17. |
| 61- | Zhou H, Xiang Q, Hu C, Zhang J, Zhang Q, Zhang R. Identification of MMP1 as a potential gene conferring erlotinib resistance in non-small cell lung cancer based on bioinformatics analyses. Hereditas. 2020 Jul 23;157(1):32. doi: 10.1186/s41065-020-00145-x. |
| 62- | Huang H, Huang Q, Tang T, Zhou X, Gu L, Lu X, Liu F. Differentially Expressed Gene Screening, Biological Function Enrichment, and Correlation with Prognosis in Non-Small Cell Lung Cancer. Med Sci Monit. 2019 Jun 10;25:4333-4341. doi: 10.12659/MSM.916962. |
| 63- | Blanco-Prieto S, Barcia-Castro L, Páez de la Cadena M, Rodríguez-Berrocal FJ, Vázquez-Iglesias L, Botana-Rial MI, Fernández-Villar A, De Chiara L. Relevance of matrix metalloproteases in non-small cell lung cancer diagnosis. BMC Cancer. 2017 Dec 5;17(1):823. doi: 10.1186/s12885-017-3842-z. |
| 64- | Kong F, Zhang R, Zhao X, Zheng G, Wang Z, Wang P. Resveratrol raises in vitro anticancer effects of paclitaxel in NSCLC cell line A549 through COX-2 expression. Korean J Physiol Pharmacol. 2017 Sep;21(5):465-474. doi: 10.4196/kjpp.2017.21.5.465. |
| 65- | Xiong F, Jiang M, Chen M, Wang X, Zhang S, Zhou J, Li K, Sheng Y, Yin L, Tang Y, Ye L, Wu M, Fu H, Zhang X. Study on Inhibitory Effect of MaiMenDong Decoction and WeiJing Decoction Combination with Cisplatin on NCI-A549 Xenograft in Nude Mice and Its Mechanism. J Cancer. 2017 Jul 23;8(13):2449-2455. doi: 10.7150/jca.17720. |
| 66- | An HJ, Lee YJ, Hong SA, Kim JO, Lee KY, Kim YK, Park JK, Kang JH. The prognostic role of tissue and serum MMP-1 and TIMP-1 expression in patients with non-small cell lung cancer. Pathol Res Pract. 2016 May;212(5):357-64. doi: 10.1016/j.prp.2015.11.014. |
| 67- | Yang B, Chen D, Zhao H, Xiao C. The effects for PM2.5 exposure on non-small-cell lung cancer induced motility and proliferation. Springerplus. 2016 Dec 1;5(1):2059. doi: 10.1186/s40064-016-3734-8. |
| 68- | Schütz A, Röser K, Klitzsch J, Lieder F, Aberger F, Gruber W, Mueller KM, Pupyshev A, Moriggl R, Friedrich K. Lung Adenocarcinomas and Lung Cancer Cell Lines Show Association of MMP-1 Expression With STAT3 Activation. Transl Oncol. 2015 Apr;8(2):97-105. doi: 10.1016/j.tranon.2015.02.002. |
| 69- | Li M, Xiao T, Zhang Y, Feng L, Lin D, Liu Y, Mao Y, Guo S, Han N, Di X, Zhang K, Cheng S, Gao Y. Prognostic significance of matrix metalloproteinase-1 levels in peripheral plasma and tumour tissues of lung cancer patients. Lung Cancer. 2010 Sep;69(3):341-7. doi: 10.1016/j.lungcan.2009.12.007. |
| 70- | Shah SA, Spinale FG, Ikonomidis JS, Stroud RE, Chang EI, Reed CE. Differential matrix metalloproteinase levels in adenocarcinoma and squamous cell carcinoma of the lung. J Thorac Cardiovasc Surg. 2010 Apr;139(4):984-90; discussion 990. doi: 10.1016/j.jtcvs.2009.12.016. |
| 71- | Lim BJ, Jung SS, Choi SY, Lee CS. Expression of metastasis-associated molecules in non-small cell lung cancer and their prognostic significance. Mol Med Rep. 2010 Jan-Feb;3(1):43-9. doi: 10.3892/mmr_00000216. |
| 72- | Su L, Zhou W, Park S, Wain JC, Lynch TJ, Liu G, Christiani DC. Matrix metalloproteinase-1 promoter polymorphism and lung cancer risk. Cancer Epidemiol Biomarkers Prev. 2005 Mar;14(3):567-70. doi: 10.1158/1055-9965.EPI-04-0482. |
| 73- | Leng D, Yi J, Xiang M, Zhao H, Zhang Y. Identification of common signatures in idiopathic pulmonary fibrosis and lung cancer using gene expression modeling. BMC Cancer. 2020 Oct 12;20(1):986. doi: 10.1186/s12885-020-07494-w. |
| 74- | Li X, Liu C, Ran R, Liu G, Yang Y, Zhao W, Xie X, Li J. Matrix metalloproteinase family gene polymorphisms and lung cancer susceptibility: an updated meta-analysis. J Thorac Dis. 2020 Mar;12(3):349-362. doi: 10.21037/jtd.2020.01.25. |
| 75- | Haung HY, Wang YC, Cheng YC, Kang W, Hu SH, Liu D, Xiao C, Wang HD. A Novel Oral Astaxanthin Nanoemulsion from Haematococcus pluvialis Induces Apoptosis in Lung Metastatic Melanoma. Oxid Med Cell Longev. 2020 Aug 26;2020:2647670. doi: 10.1155/2020/2647670. |
| 76- | Sun C, Yang J, Cheng HB, Shen WX, Jiang ZQ, Wu MJ, Li L, Li WT, Chen TT, Rao XW, Zhou JR, Wu MH. 2-Hydroxy-3-methylanthraquinone inhibits lung carcinoma cells through modulation of IL-6-induced JAK2/STAT3 pathway. Phytomedicine. 2019 Aug;61:152848. doi: 10.1016/j.phymed.2019.152848. |
| 77- | Chu CN, Wu KC, Chung WS, Zheng LC, Juan TK, Hsiao YT, Peng SF, Yang JL, Ma YS, Wu RS, Chung JG. Etomidate Suppresses Invasion and Migration of Human A549 Lung Adenocarcinoma Cells. Anticancer Res. 2019 Jan;39(1):215-223. doi: 10.21873/anticanres.13100. |
| 78- | Li W, Zhang X, Li Z, Jiang F, Zhao H, Wei B. Identification of genes associated with matrix metalloproteinases in invasive lung adenocarcinoma. Oncol Lett. 2018 Jul;16(1):123-130. doi: 10.3892/ol.2018.8683. |
| 79- | He Z, Huang C, Lin G, Ye Y. siRNA-induced TRAF6 knockdown promotes the apoptosis and inhibits the invasion of human lung cancer SPC-A1 cells. Oncol Rep. 2016 Apr;35(4):1933-40. doi: 10.3892/or.2016.4602. |
| 80- | Huang HC, Tsai LL, Tsai JP, Hsieh SC, Yang SF, Hsueh JT, Hsieh YH. Licochalcone A inhibits the migration and invasion of human lung cancer cells via inactivation of the Akt signaling pathway with downregulation of MMP-1/-3 expression. Tumour Biol. 2014 Dec;35(12):12139-49. doi: 10.1007/s13277-014-2519-3. |
| 81- | Younes M, Wu Z, Dupouy S, Lupo AM, Mourra N, Takahashi T, Fléjou JF, Trédaniel J, Régnard JF, Damotte D, Alifano M, Forgez P. Neurotensin (NTS) and its receptor (NTSR1) causes EGFR, HER2 and HER3 over-expression and their autocrine/paracrine activation in lung tumors, confirming responsiveness to erlotinib. Oncotarget. 2014 Sep 30;5(18):8252-69. doi: 10.18632/oncotarget.1633. |
| 82- | Li X, Tai HH. Thromboxane A2 receptor-mediated release of matrix metalloproteinase-1 (MMP-1) induces expression of monocyte chemoattractant protein-1 (MCP-1) by activation of protease-activated receptor 2 (PAR2) in A549 human lung adenocarcinoma cells. Mol Carcinog. 2014 Aug;53(8):659-66. doi: 10.1002/mc.22020. |
| 83- | Xiao XY, Wang XD, Zang DY. MMP1-1607 1G/2G polymorphism and lung cancer risk: a meta-analysis. Tumour Biol. 2012 Dec;33(6):2385-92. doi: 10.1007/s13277-012-0502-4. |
| 84- | Ikari A, Sato T, Watanabe R, Yamazaki Y, Sugatani J. Increase in claudin-2 expression by an EGFR/MEK/ERK/c-Fos pathway in lung adenocarcinoma A549 cells. Biochim Biophys Acta. 2012 Jun;1823(6):1110-8. doi: 10.1016/j.bbamcr.2012.04.005. |
| 85- | Mishra DK, Sakamoto JH, Thrall MJ, Baird BN, Blackmon SH, Ferrari M, Kurie JM, Kim MP. Human lung cancer cells grown in an ex vivo 3D lung model produce matrix metalloproteinases not produced in 2D culture. PLoS One. 2012;7(9):e45308. doi: 10.1371/journal.pone.0045308. |
| 86- | Hart K, Landvik NE, Lind H, Skaug V, Haugen A, Zienolddiny S. A combination of functional polymorphisms in the CASP8, MMP1, IL10 and SEPS1 genes affects risk of non-small cell lung cancer. Lung Cancer. 2011 Feb;71(2):123-9. doi: 10.1016/j.lungcan.2010.04.016. |
| 87- | Klinchid J, Chewaskulyoung B, Saeteng S, Lertprasertsuke N, Kasinrerk W, Cressey R. Effect of combined genetic polymorphisms on lung cancer risk in northern Thai women. Cancer Genet Cytogenet. 2009 Dec;195(2):143-9. doi: 10.1016/j.cancergencyto.2009.08.011. |
| 88- | Armstrong DA, Phelps LN, Vincenti MP. CCAAT enhancer binding protein-beta regulates matrix metalloproteinase-1 expression in interleukin-1beta-stimulated A549 lung carcinoma cells. Mol Cancer Res. 2009 Sep;7(9):1517-24. doi: 10.1158/1541-7786.MCR-09-0082. |
| 89- | Sagindikova GE, Kogan EA, Satbaeva EB, Paramonova NB. [Matrix metalloproteinases, their inhibitors and angiogenesis in different morphological types of lung precancer in persons who have long lived in the radioactive substance-polluted area of the Semipalatinsk Region, Kazakhstan]. Arkh Patol. 2008 Mar-Apr;70(2):21-5. |
| 90- | Savaraj N, Wei Y, Unate H, Liu PM, Wu CJ, Wangpaichitr M, Xia D, Xu HJ, Hu SX, Tien Kuo M. Redox regulation of matrix metalloproteinase gene family in small cell lung cancer cells. Free Radic Res. 2005 Apr;39(4):373-81. doi: 10.1080/10715760400029694. |
| 91- | Xia SH, Wang J, Kang JX. Decreased n-6/n-3 fatty acid ratio reduces the invasive potential of human lung cancer cells by downregulation of cell adhesion/invasion-related genes. Carcinogenesis. 2005 Apr;26(4):779-84. doi: 10.1093/carcin/bgi019. |
| 92- | D'Agostino P, Camemi AR, Caruso R, Arcoleo F, Cascio A, Dolce A, Sacco E, Cangemi G, di Rosa T, Moceo P, Cillari E. Matrix metalloproteinases production in malignant pleural effusions after talc pleurodesis. Clin Exp Immunol. 2003 Oct;134(1):138-42. doi: 10.1046/j.1365-2249.2003.02262.x. |
| 93- | Ouyang QC, Hu CP, Liang QH. [Gene expression of MMP1 and TIMP1 in lung cancer detected with a cDNA microarray technique]. Hunan Yi Ke Da Xue Xue Bao. 2003 Jun;28(3):227-8. |
| 94- | Decock J, Thirkettle S, Wagstaff L, Edwards DR. Matrix metalloproteinases: protective roles in cancer. J Cell Mol Med. 2011 Jun;15(6):1254-65. doi: 10.1111/j.1582-4934.2011.01302.x. |
| 95- | Morishita A, Gerber A, Gow CH, Zelonina T, Chada K, D'Armiento J. Cell Specific Matrix Metalloproteinase-1 Regulates Lung Metastasis Synergistically with Smoke Exposure. J Cancer Res Forecast. 2018;1(2):1014. |
| 96- | Dong QZ, Zhao Y, Liu Y, Wang Y, Zhang PX, Jiang GY, Dong XJ, Cui QZ, Wang EH. Overexpression of SCC-S2 correlates with lymph node metastasis and poor prognosis in patients with non-small-cell lung cancer. Cancer Sci. 2010 Jun;101(6):1562-9. doi: 10.1111/j.1349-7006.2010.01557.x. |
| 97- | Shen TC, Chang WS, Tsai CW, Chao CY, Lin YT, Hsiao CL, Hsu CL, Chen WC, Hsia TC, Bau DT. The Contribution of Matrix Metalloproteinase-1 Promoter Genotypes in Taiwan Lung Cancer Risk. Anticancer Res. 2018 Jan;38(1):253-257. doi: 10.21873/anticanres.12215. |
| 98- | Pulukuri SM, Rao JS. Matrix metalloproteinase-1 promotes prostate tumor growth and metastasis. Int J Oncol. 2008 Apr;32(4):757-65. |
| 99- | Pereira JL, Gomes M, Teixeira AL, Coelho A, Rolfo C, Araújo A. Potential and importance of metalloproteinases and interleukins in inflammation and metastasization in non-small cell lung cancer. Transl Cancer Res 2018;7(3):782-795. doi: 10.21037/tcr.2018.05.16 |
| 100- | Wang JC, Liu XP, Nie XY, Wu GQ, Zhang WH, Shen L, Yao LB. Expression, Purification and Functional Identification of Extracellular Part of Discoidin Domain Receptor 2. Sheng Wu Hua Xue Yu Sheng Wu Wu Li Xue Bao (Shanghai). 2001;33(6):647-652. |
| 101- | Maekawa R, Maki H, Wada T, Yoshida H, Nishida-Nishimoto K, Okamoto H, Matsumoto Y, Tsuzuki H, Yoshioka T. Anti-metastatic efficacy and safety of MMI-166, a selective matrix metalloproteinase inhibitor. Clin Exp Metastasis. 2000;18(1):61-6. doi: 10.1023/a:1026553414492. |
| 102- | Michael M, Babic B, Khokha R, Tsao M, Ho J, Pintilie M, Leco K, Chamberlain D, Shepherd FA. Expression and prognostic significance of metalloproteinases and their tissue inhibitors in patients with small-cell lung cancer. J Clin Oncol. 1999 Jun;17(6):1802-8. doi: 10.1200/JCO.1999.17.6.1802. |
| 103- | Herrera I, Cisneros J, Maldonado M, Ramírez R, Ortiz-Quintero B, Anso E, Chandel NS, Selman M, Pardo A. Matrix metalloproteinase (MMP)-1 induces lung alveolar epithelial cell migration and proliferation, protects from apoptosis, and represses mitochondrial oxygen consumption. J Biol Chem. 2013 Sep 6;288(36):25964-25975. doi: 10.1074/jbc.M113.459784. |
| 104- | Moche M, Hui DS, Huse K, Chan KS, Choy DK, Scholz GH, Gosse H, Winkler J, Schauer J, Sack U, Hoheisel G. Matrix-Metalloproteinasen und deren Inhibitoren bei Lungenkarzinomen mit malignem Pleuraerguss [Matrix metalloproteinases and their inhibitors in lung cancer with malignant pleural effusion]. Pneumologie. 2005 Aug;59(8):523-8. German. doi: 10.1055/s-2005-870966. |
| 105- | Yamamoto A, Yano S, Shiraga M, Ogawa H, Goto H, Miki T, Zhang H, Sone S. A third-generation matrix metalloproteinase (MMP) inhibitor (ONO-4817) combined with docetaxel suppresses progression of lung micrometastasis of MMP-expressing tumor cells in nude mice. Int J Cancer. 2003 Mar 1;103(6):822-8. doi: 10.1002/ijc.10875. |
| 106- | Yamaura T, Murakami K, Doki Y, Sugiyama S, Misaki T, Yamada Y, Saiki I. Solitary lung tumors and their spontaneous metastasis in athymic nude mice orthotopically implanted with human non-small cell lung cancer. Neoplasia. 2000 Jul-Aug;2(4):315-24. doi: 10.1038/sj.neo.7900098. |
| 107- | Wang M, Chen X, Fu G, Ge M. Glutathione peroxidase 2 overexpression promotes malignant progression and cisplatin resistance of KRAS‑mutated lung cancer cells. Oncol Rep. 2022 Dec;48(6):207. doi: 10.3892/or.2022.8422. |
| 108- | Hamada Ji, Omatsu T, Okada F, Furuuchi K, Okubo Y, Takahashi Y, Tada M, Miyazaki YJ, Taniguchi Y, Shirato H, Miyasaka K, Moriuchi T. Overexpression of homeobox gene HOXD3 induces coordinate expression of metastasis-related genes in human lung cancer cells. Int J Cancer. 2001 Aug 15;93(4):516-25. doi: 10.1002/ijc.1357. |
| 109- | Fan S, Liao Y, Liu C, Huang Q, Liang H, Ai B, Fu S, Zhou S. Estrogen promotes tumor metastasis via estrogen receptor beta-mediated regulation of matrix-metalloproteinase-2 in non-small cell lung cancer. Oncotarget. 2017 Apr 10;8(34):56443-56459. doi: 10.18632/oncotarget.16992. |

**CD151:**

| 1-       Peng D, Li PC, Liu T, Zeng HS, Fei YJ, Liu ZX, Zuo HJ. Key Role of CD151-integrin Complex in Lung Cancer Metastasis and Mechanisms Involved. Curr Med Sci. 2020;40(6):1148-1155. doi: 10.1007/s11596-020-2297-7. |
| --- |
| 2-      Kwon MJ, Seo J, Kim YJ, Kwon MJ, Choi JY, Kim TE, Lee DH, Park S, Shin YK, Han J, Choi YL. Prognostic significance of CD151 overexpression in non-small cell lung cancer. Lung Cancer. 2013;81(1):109-16. doi: 10.1016/j.lungcan.2013.03.014. |
| 3-       Zhu J, Cai T, Zhou J, Du W, Zeng Y, Liu T, Fu Y, Li Y, Qian Q, Yang XH, Li Q, Huang JA, Liu Z. CD151 drives cancer progression depending on integrin α3β1 through EGFR signaling in non-small cell lung cancer. J Exp Clin Cancer Res. 2021;40(1):192. doi: 10.1186/s13046-021-01998-4. |
| 4-       Suzuki S, Miyazaki T, Tanaka N, Sakai M, Sano A, Inose T, Sohda M, Nakajima M, Kato H, Kuwano H. Prognostic significance of CD151 expression in esophageal squamous cell carcinoma with aggressive cell proliferation and invasiveness. Ann Surg Oncol. 2011;18(3):888-93. doi: 10.1245/s10434-010-1387-3. |
| 5-       Matsumoto N, Morine Y, Utsunomiya T, Imura S, Ikemoto T, Arakawa Y, Iwahashi S, Saito Y, Yamada S, Ishikawa D, Takasu C, Miyake H, Shimada M. Role of CD151 expression in gallbladder carcinoma. Surgery. 2014; 156(5):1212-7. doi: 10.1016/j.surg.2014.04.053. |
| 6-       Yang YM, Zhang ZW, Liu QM, Sun YF, Yu JR, Xu WX. Overexpression of CD151 predicts prognosis in patients with resected gastric cancer. PLoS One. 2013;8(3):e58990. doi: 10.1371/journal.pone.0058990. |
| 7-       Zhu GH, Huang C, Qiu ZJ, Liu J, Zhang ZH, Zhao N, Feng ZZ, Lv XH. Expression and prognostic significance of CD151, c-Met, and integrin alpha3/alpha6 in pancreatic ductal adenocarcinoma. Dig Dis Sci. 2011 Apr;56(4):1090-8. doi: 10.1007/s10620-010-1416-x. |

**PVT1:**

| 1.       Cui D, Yu CH, Liu M, Xia QQ, Zhang YF, Jiang WL. Long non-coding RNA PVT1 as a novel biomarker for diagnosis and prognosis of non-small cell lung cancer. Tumour Biol. 2016;37(3):4127-34. doi: 10.1007/s13277-015-4261-x. |
| --- |
| 2.       Yang YR, Zang SZ, Zhong CL, Li YX, Zhao SS, Feng XJ. Increased expression of the lncRNA PVT1 promotes tumorigenesis in non-small cell lung cancer. Int J Clin Exp Pathol. 2014;7(10):6929-35. |
| 3.       Huang C, Liu S, Wang H, Zhang Z, Yang Q, Gao F. LncRNA PVT1 overexpression is a poor prognostic biomarker and regulates migration and invasion in small cell lung cancer. Am J Transl Res. 2016; 8(11):5025-5034. |
| 4.       Lu D, Luo P, Wang Q, Ye Y, Wang B. lncRNA PVT1 in cancer: A review and meta-analysis. Clin Chim Acta. 2017; 474:1-7. doi: 10.1016/j.cca.2017.08.038. |
| 5.       Wan L, Sun M, Liu GJ, Wei CC, Zhang EB, Kong R, Xu TP, Huang MD, Wang ZX. Long Noncoding RNA PVT1 Promotes Non-Small Cell Lung Cancer Cell Proliferation through Epigenetically Regulating LATS2 Expression. Mol Cancer Ther. 2016;15(5):1082-94. doi: 10.1158/1535-7163.MCT-15-0707. |
| 6.       Zhu S, Shuai P, Yang C, Zhang Y, Zhong S, Liu X, Chen K, Ran Q, Yang H, Zhou Y. Prognostic value of long non-coding RNA PVT1 as a novel biomarker in various cancers: a meta-analysis. Oncotarget. 2017;8(68):113174-113184. doi: 10.18632/oncotarget.22830. |
| 7.       Liu C, Jin J, Liang D, Gao Z, Zhang Y, Guo T, He Y. Long Noncoding RNA PVT1 as a Novel Predictor of Metastasis, Clinicopathological Characteristics and Prognosis in Human Cancers: a Meta-Analysis. Pathol Oncol Res. 2019;25(3):837-847. doi: 10.1007/s12253-018-0451-3. |
| 8.       Ren X, Cao D, Yang L, Li X, Zhang W, Xiao Y, Xi Y, Li F, Li D, Pan Z. High Expression of long non-coding RNA PVT1 predicts metastasis in Han and Uygur Patients with Gastric Cancer in Xinjiang, China. Sci Rep. 2019;9(1):548. doi: 10.1038/s41598-018-36985-x. |
| 9.       Huang T, Liu HW, Chen JQ, Wang SH, Hao LQ, Liu M, Wang B. The long noncoding RNA PVT1 functions as a competing endogenous RNA by sponging miR-186 in gastric cancer. Biomed Pharmacother. 2017;88:302-308. doi: 10.1016/j.biopha.2017.01.049. |
| 10.    Ma C, Nie XG, Wang YL, Wu DP, Liang QD. Meta-analysis of the prognostic value of long non-coding RNA PVT1 for cancer patients. Medicine (Baltimore). 2018; 97(49):e13548. doi: 10.1097/MD.0000000000013548. |
| 11.    Yu C, Wang Y, Li G, She L, Zhang D, Chen X, Zhang X, Qin Z, Cao H, Liu Y. LncRNA PVT1 promotes malignant progression in squamous cell carcinoma of the head and neck. J Cancer. 2018; 9(19):3593-3602. doi: 10.7150/jca.26465. |
| 12.    Wang W. Z, Liu L, Jia S. Q, Qu H. F. Long noncoding RNA PVT-1 predicts poor patient prognosis in non-small cell lung cancer. Int J Clin Exp Pathol. 2016;9(1): 171-175.‏ |
| 13.    Cui Y, Liu L, Chen J, Li C, Cheng X, Zu, X. B. Increased expression of long non-coding RNA PVT1 correlates with clinical progression and poor prognosis in bladder cancer. Int J Clin Exp Pathol. 2017; 10(3): 3265-3271. |
| 14.    Yu X, Zhao J, He Y. Long non-coding RNA PVT1 functions as an oncogene in human colon cancer through miR-30d-5p/RUNX2 axis. J BUON. 2018;23(1):48-54. |
| 15.    Wang Z, Su M, Xiang B, Zhao K, Qin B. Circular RNA PVT1 promotes metastasis via miR-145 sponging in CRC. Biochem Biophys Res Commun. 2019; 512(4):716-722. doi: 10.1016/j.bbrc.2019.03.121. |
| 16.    Niu J, Song X, Zhang X. Regulation of lncRNA PVT1 on miR-125 in metastasis of gastric cancer cells. Oncol Lett. 2020;19(2):1261-1266. doi: 10.3892/ol.2019.11195. |
| 17.    Wang L, Xiao B, Yu T, Gong L, Wang Y, Zhang X, Zou Q, Zuo Q. lncRNA PVT1 promotes the migration of gastric cancer by functioning as ceRNA of miR-30a and regulating Snail. J Cell Physiol. 2021; 236(1):536-548. doi: 10.1002/jcp.29881. |
| 18.    Pan Y, Liu L, Cheng Y, Yu J, Feng Y. Amplified LncRNA PVT1 promotes lung cancer proliferation and metastasis by facilitating VEGFC expression. Biochem Cell Biol. 2020; 98(6):676-682. doi: 10.1139/bcb-2019-0435. |

**SKP2:**

| 1.       Ye J, Zhou G, Zhang Z, Sun L, He X, Zhou J.Effects of Skp2 expression on the recurrence and prognosis of esophageal squamous cell carcinoma after complete macroscopic resection. Int. J. Clin. Exp. Pathol. 2016; 9(6): 6469-6475.‏ |
| --- |
| 2.       Takanami I. The prognostic value of overexpression of Skp2 mRNA in non-small cell lung cancer. Oncol Rep. 2005; 13(4):727-31. |
| 3.       Yokoi S, Yasui K, Mori M, Iizasa T, Fujisawa T, Inazawa J. Amplification and overexpression of SKP2 are associated with metastasis of non-small-cell lung cancers to lymph nodes. Am J Pathol. 2004; 165(1):175-80. doi: 10.1016/S0002-9440(10)63286-5. |
| 4.       Zhong K, Yang F, Han Q, Chen J, Wang J. Skp2 expression has different clinicopathological and prognostic implications in lung adenocarcinoma and squamous cell carcinoma. Oncol Lett. 2018;16(3):2873-2880. doi: 10.3892/ol.2018.9000. |
| 5.       Hung WC, Tseng WL, Shiea J, Chang HC. Skp2 overexpression increases the expression of MMP-2 and MMP-9 and invasion of lung cancer cells. Cancer Lett. 2010; 288(2):156-61. doi: 10.1016/j.canlet.2009.06.032. |
| 6.       Gao H, Zhong L, Hu K. [Expression of Skp2 and its correlation with c-myc in non-small cell lung cancer]. Zhongguo Fei Ai Za Zhi. 2004; 7(6):493-6. Chinese. doi: 10.3779/j.issn.1009-3419.2004.06.07. |
| 7.       Zhao J, Yang CL, Zhao SY. [Expression of SKP2 protein in lung carcinoma and its implication for prognosis]. Zhonghua Zhong Liu Za Zhi. 2007 Apr;29(4):289-92. |
| 8.       Qiu L, Lv J, Chen Y, Wang J, Wu R. Expression of Skp2 and p27kip1 proteins in hypopharyngeal squamous cell carcinoma and its clinical significance. Oncol Lett. 2015; 10(6):3756-3760. doi: 10.3892/ol.2015.3799. |
| 9.    Wang XC, Wu YP, Ye B, Lin DC, Feng YB, Zhang ZQ, Xu X, Han YL, Cai Y, Dong JT, Zhan QM, Wu M, Wang MR. Suppression of anoikis by SKP2 amplification and overexpression promotes metastasis of esophageal squamous cell carcinoma. Mol Cancer Res. 2009; 7(1):12-22. doi: 10.1158/1541-7786.MCR-08-0092. |
