# Supplementary material for: Genes whose expressions in the primary lung squamous cell carcinoma are able to accurately predict the progression of metastasis through lymphatic system, inferred from a bioinformatics analyses
Source: Sci Rep. 2023 Apr 25;13:6733. doi: 10.1038/s41598-023-33897-3 (PMC10130036; doi:10.1038/s41598-023-33897-3)
Supplement: Supplementary file 4 — Supplementary Information 4. [file 41598_2023_33897_MOESM4_ESM.doc]

**NDC80**

|  | | 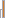 | **Protein Interactions** | 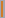 |  | | --- | --- | --- | --- |  |  | |  | | --- | | | **PROTEIN INTERACTORS** |  |  |  |  | | --- | --- | --- | --- | --- | | **Name of Interactor** |  | **Experiment Type** |  | **Type** | | [Angiomotin like 2](http://www.hprd.org/interactions?hprd_id=16485&isoform_id=16485_1&isoform_name=Isoform_1) |  | [Yeast 2 Hybrid](http://www.ncbi.nlm.nih.gov/entrez/query.fcgi?cmd=Retrieve&db=PubMed&list_uids=,16189514,&dopt=Abstract) |  | Direct | | [Cell division cycle associated 1](http://www.hprd.org/interactions?hprd_id=10817&isoform_id=10817_1&isoform_name=Isoform_1) |  | [Yeast 2 Hybrid](http://www.ncbi.nlm.nih.gov/entrez/query.fcgi?cmd=Retrieve&db=PubMed&list_uids=,14602875,&dopt=Abstract) |  | Direct | | [CGI-116 protein](http://www.hprd.org/interactions?hprd_id=13031&isoform_id=13031_1&isoform_name=Isoform_1) |  | [Yeast 2 Hybrid](http://www.ncbi.nlm.nih.gov/entrez/query.fcgi?cmd=Retrieve&db=PubMed&list_uids=,16189514,&dopt=Abstract) |  | Direct | | [Hypothetical protein KIAA1536](http://www.hprd.org/interactions?hprd_id=13868&isoform_id=13868_1&isoform_name=Isoform_1) |  | [Yeast 2 Hybrid](http://www.ncbi.nlm.nih.gov/entrez/query.fcgi?cmd=Retrieve&db=PubMed&list_uids=,16189514,&dopt=Abstract) |  | Direct | | [Kelch like 12](http://www.hprd.org/interactions?hprd_id=13925&isoform_id=13925_1&isoform_name=Isoform_1) |  | [Yeast 2 Hybrid](http://www.ncbi.nlm.nih.gov/entrez/query.fcgi?cmd=Retrieve&db=PubMed&list_uids=,16189514,&dopt=Abstract) |  | Direct | | [MAD1 mitotic arrest deficient-like 1](http://www.hprd.org/interactions?hprd_id=04065&isoform_id=04065_1&isoform_name=Isoform_1) |  | [In Vivo ; Yeast 2 Hybrid](http://www.ncbi.nlm.nih.gov/entrez/query.fcgi?cmd=Retrieve&db=PubMed&list_uids=,12351790,&dopt=Abstract) |  | Direct | | [MAD2 mitotic arrest deficient like 1](http://www.hprd.org/interactions?hprd_id=03274&isoform_id=03274_1&isoform_name=Isoform_1) |  | [In Vivo](http://www.ncbi.nlm.nih.gov/entrez/query.fcgi?cmd=Retrieve&db=PubMed&list_uids=,12351790,&dopt=Abstract) |  | Direct | | [Pallidin homolog](http://www.hprd.org/interactions?hprd_id=16055&isoform_id=16055_1&isoform_name=Isoform_1) |  | [Yeast 2 Hybrid](http://www.ncbi.nlm.nih.gov/entrez/query.fcgi?cmd=Retrieve&db=PubMed&list_uids=,16189514,&dopt=Abstract) |  | Direct | | [Proteasome 26S subunit, ATPase 2](http://www.hprd.org/interactions?hprd_id=01105&isoform_id=01105_1&isoform_name=Isoform_1) |  | [In Vitro ; Yeast 2 Hybrid](http://www.ncbi.nlm.nih.gov/entrez/query.fcgi?cmd=Retrieve&db=PubMed&list_uids=,9295362,&dopt=Abstract) |  | Direct | | [Retinoblastoma 1](http://www.hprd.org/interactions?hprd_id=01574&isoform_id=01574_1&isoform_name=Isoform_1) |  | [Yeast 2 Hybrid](http://www.ncbi.nlm.nih.gov/entrez/query.fcgi?cmd=Retrieve&db=PubMed&list_uids=,10409732,&dopt=Abstract) |  | Direct | | [SMC1](http://www.hprd.org/interactions?hprd_id=02077&isoform_id=02077_1&isoform_name=Isoform_1) |  | [In Vitro ; Yeast 2 Hybrid](http://www.ncbi.nlm.nih.gov/entrez/query.fcgi?cmd=Retrieve&db=PubMed&list_uids=,9295362,&dopt=Abstract) |  | Direct | | [Usher syndrome 1C binding protein 1](http://www.hprd.org/interactions?hprd_id=18276&isoform_id=18276_1&isoform_name=Isoform_1) |  | [Yeast 2 Hybrid](http://www.ncbi.nlm.nih.gov/entrez/query.fcgi?cmd=Retrieve&db=PubMed&list_uids=,16189514,&dopt=Abstract) |  | Direct | | [ZW10 interacting protein 1](http://www.hprd.org/interactions?hprd_id=18366&isoform_id=18366_1&isoform_name=Isoform_1) |  | [In Vitro ; Yeast 2 Hybrid](http://www.ncbi.nlm.nih.gov/entrez/query.fcgi?cmd=Retrieve&db=PubMed&list_uids=,16732327,&dopt=Abstract) |  | Direct | | [Never in mitosis gene A-related kinase 2](http://www.hprd.org/interactions?hprd_id=04953&isoform_id=04953_1&isoform_name=Isoform_1) |  | [In Vivo ; In Vitro ; Yeast 2 Hybrid](http://www.ncbi.nlm.nih.gov/entrez/query.fcgi?cmd=Retrieve&db=PubMed&list_uids=,12386167,&dopt=Abstract) |  | Direct | | [Kinetochore associated 2](http://www.hprd.org/interactions?hprd_id=06277&isoform_id=06277_1&isoform_name=Isoform_1) |  | [Yeast 2 Hybrid](http://www.ncbi.nlm.nih.gov/entrez/query.fcgi?cmd=Retrieve&db=PubMed&list_uids=,9295362,14602875,&dopt=Abstract) |  | Direct | | [MIS12](http://www.hprd.org/interactions?hprd_id=12379&isoform_id=12379_1&isoform_name=Isoform_1) |  | [In Vivo ; In Vitro](http://www.ncbi.nlm.nih.gov/entrez/query.fcgi?cmd=Retrieve&db=PubMed&list_uids=,15502821,&dopt=Abstract) |  | Direct | | [Kinetochore protein Spc25](http://www.hprd.org/interactions?hprd_id=15465&isoform_id=15465_1&isoform_name=Isoform_1) |  | [In Vivo ; In Vitro](http://www.ncbi.nlm.nih.gov/entrez/query.fcgi?cmd=Retrieve&db=PubMed&list_uids=,14699129,&dopt=Abstract) |  | Direct | | [TOM1 like 1](http://www.hprd.org/interactions?hprd_id=05266&isoform_id=05266_1&isoform_name=Isoform_1) |  | [Yeast 2 Hybrid](http://www.ncbi.nlm.nih.gov/entrez/query.fcgi?cmd=Retrieve&db=PubMed&list_uids=,16169070,&dopt=Abstract) |  | Direct | | [Serine/threonine protein kinase 6](http://www.hprd.org/interactions?hprd_id=04066&isoform_id=04066_1&isoform_name=Isoform_1) |  | [In Vitro](http://www.ncbi.nlm.nih.gov/entrez/query.fcgi?cmd=Retrieve&db=PubMed&list_uids=,14602875,&dopt=Abstract) |  | Direct | | [Aurora kinase B](http://www.hprd.org/interactions?hprd_id=05397&isoform_id=05397_1&isoform_name=Isoform_1) |  | [In Vivo ; In Vitro](http://www.ncbi.nlm.nih.gov/entrez/query.fcgi?cmd=Retrieve&db=PubMed&list_uids=,14602875,&dopt=Abstract) |  | Direct | | [CDCA1](http://www.hprd.org/interactions?hprd_id=10817&isoform_id=10817_1&isoform_name=Isoform_1) [Kinetochore protein Spc24](http://www.hprd.org/interactions?hprd_id=15464&isoform_id=15464_1&isoform_name=Isoform_1) [Kinetochore protein Spc25](http://www.hprd.org/interactions?hprd_id=15465&isoform_id=15465_1&isoform_name=Isoform_1) |  | [In Vivo](http://www.ncbi.nlm.nih.gov/entrez/query.fcgi?cmd=Retrieve&db=PubMed&list_uids=14699129&dopt=Abstract) |  | Complex | | [ZW10 interacting protein 1](http://www.hprd.org/interactions?hprd_id=18366&isoform_id=18366_1&isoform_name=Isoform_1) [C1orf48 protein](http://www.hprd.org/interactions?hprd_id=12378&isoform_id=12378_1&isoform_name=Isoform_1) [Chromosome 20 open reading frame 172 protein](http://www.hprd.org/interactions?hprd_id=16455&isoform_id=16455_1&isoform_name=Isoform_1) [MIS12](http://www.hprd.org/interactions?hprd_id=12379&isoform_id=12379_1&isoform_name=Isoform_1) [Kinetochore protein Spc24](http://www.hprd.org/interactions?hprd_id=15464&isoform_id=15464_1&isoform_name=Isoform_1) [Cancer susceptibility candidate 5](http://www.hprd.org/interactions?hprd_id=10634&isoform_id=10634_1&isoform_name=Isoform_1) [Lipoprotein, Lp like 2](http://www.hprd.org/interactions?hprd_id=17441&isoform_id=17441_1&isoform_name=Isoform_1) |  | [In Vivo](http://www.ncbi.nlm.nih.gov/entrez/query.fcgi?cmd=Retrieve&db=PubMed&list_uids=15824131&dopt=Abstract) |  | Complex | | [ZW10 interacting protein 1](http://www.hprd.org/interactions?hprd_id=18366&isoform_id=18366_1&isoform_name=Isoform_1) [ZW10](http://www.hprd.org/interactions?hprd_id=04902&isoform_id=04902_1&isoform_name=Isoform_1) |  | [In Vivo](http://www.ncbi.nlm.nih.gov/entrez/query.fcgi?cmd=Retrieve&db=PubMed&list_uids=16732327&dopt=Abstract) |  | Complex | | | | --- | --- | --- | --- | --- | --- | --- | --- | --- | --- | --- | --- | --- | --- | --- | --- | --- | --- | --- | --- | --- | --- | --- | --- | --- | --- | --- | --- | --- | --- | --- | --- | --- | --- | --- | --- | --- | --- | --- | --- | --- | --- | --- | --- | --- | --- | --- | --- | --- | --- | --- | --- | --- | --- | --- | --- | --- | --- | --- | --- | --- | --- | --- | --- | --- | --- | --- | --- | --- | --- | --- | --- | --- | --- | --- | --- | --- | --- | --- | --- | --- | --- | --- | --- | --- | --- | --- | --- | --- | --- | --- | --- | --- | --- | --- | --- | --- | --- | --- | --- | --- | --- | --- | --- | --- | --- | --- | --- | --- | --- | --- | --- | --- | --- | --- | --- | --- | --- | --- | --- | --- | --- | --- | --- | --- | --- | --- | --- | --- | |
| --- | --- | --- | --- | --- | --- | --- | --- | --- | --- | --- | --- | --- | --- | --- | --- | --- | --- | --- | --- | --- | --- | --- | --- | --- | --- | --- | --- | --- | --- | --- | --- | --- | --- | --- | --- | --- | --- | --- | --- | --- | --- | --- | --- | --- | --- | --- | --- | --- | --- | --- | --- | --- | --- | --- | --- | --- | --- | --- | --- | --- | --- | --- | --- | --- | --- | --- | --- | --- | --- | --- | --- | --- | --- | --- | --- | --- | --- | --- | --- | --- | --- | --- | --- | --- | --- | --- | --- | --- | --- | --- | --- | --- | --- | --- | --- | --- | --- | --- | --- | --- | --- | --- | --- | --- | --- | --- | --- | --- | --- | --- | --- | --- | --- | --- | --- | --- | --- | --- | --- | --- | --- | --- | --- | --- | --- | --- | --- | --- | --- | --- | --- | --- | --- | --- |

**PCNA**

|  | | 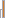 | **Protein Interactions** | 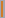 |  | | --- | --- | --- | --- |  |  | |  | | --- | | | **PROTEIN INTERACTORS** |  |  |  |  | | --- | --- | --- | --- | --- | | **Name of Interactor** |  | **Experiment Type** |  | **Type** | | [ADP ribosyl transferase](http://www.hprd.org/interactions?hprd_id=01435&isoform_id=01435_1&isoform_name=Isoform_1) |  | [In Vivo](http://www.ncbi.nlm.nih.gov/entrez/query.fcgi?cmd=Retrieve&db=PubMed&list_uids=,12930846,&dopt=Abstract) |  | Direct | | [Apex nuclease](http://www.hprd.org/interactions?hprd_id=00136&isoform_id=00136_1&isoform_name=Isoform_1) |  | [In Vivo](http://www.ncbi.nlm.nih.gov/entrez/query.fcgi?cmd=Retrieve&db=PubMed&list_uids=,11601988,10559261,&dopt=Abstract) |  | Direct | | [CDC2](http://www.hprd.org/interactions?hprd_id=00302&isoform_id=00302_1&isoform_name=Isoform_1) |  | [In Vitro](http://www.ncbi.nlm.nih.gov/entrez/query.fcgi?cmd=Retrieve&db=PubMed&list_uids=,7949095,&dopt=Abstract) |  | Direct | | [CDC25C](http://www.hprd.org/interactions?hprd_id=01146&isoform_id=01146_1&isoform_name=Isoform_1) |  | [In Vivo ; In Vitro ; Yeast 2 Hybrid](http://www.ncbi.nlm.nih.gov/entrez/query.fcgi?cmd=Retrieve&db=PubMed&list_uids=,11896603,&dopt=Abstract) |  | Direct | | [Cyclin B1](http://www.hprd.org/interactions?hprd_id=00454&isoform_id=00454_1&isoform_name=Isoform_1) |  | [In Vivo](http://www.ncbi.nlm.nih.gov/entrez/query.fcgi?cmd=Retrieve&db=PubMed&list_uids=,8101826,&dopt=Abstract) |  | Direct | | [Cyclin D1](http://www.hprd.org/interactions?hprd_id=01346&isoform_id=01346_1&isoform_name=Isoform_1) |  | [In Vitro](http://www.ncbi.nlm.nih.gov/entrez/query.fcgi?cmd=Retrieve&db=PubMed&list_uids=,7908906,&dopt=Abstract) |  | Direct | | [Cyclin D3](http://www.hprd.org/interactions?hprd_id=00452&isoform_id=00452_1&isoform_name=Isoform_1) |  | [In Vitro](http://www.ncbi.nlm.nih.gov/entrez/query.fcgi?cmd=Retrieve&db=PubMed&list_uids=,7908906,&dopt=Abstract) |  | Direct | | [Cyclin dependent kinase 2](http://www.hprd.org/interactions?hprd_id=00310&isoform_id=00310_1&isoform_name=Isoform_1) |  | [In Vitro](http://www.ncbi.nlm.nih.gov/entrez/query.fcgi?cmd=Retrieve&db=PubMed&list_uids=,10930425,&dopt=Abstract) |  | Direct | | [Cyclin dependent kinase 5](http://www.hprd.org/interactions?hprd_id=00449&isoform_id=00449_1&isoform_name=Isoform_1) |  | [In Vitro](http://www.ncbi.nlm.nih.gov/entrez/query.fcgi?cmd=Retrieve&db=PubMed&list_uids=,7949095,&dopt=Abstract) |  | Direct | | [Cyclin dependent kinase inhibitor 1A](http://www.hprd.org/interactions?hprd_id=00298&isoform_id=00298_1&isoform_name=Isoform_1) |  | [In Vivo ; In Vitro](http://www.ncbi.nlm.nih.gov/entrez/query.fcgi?cmd=Retrieve&db=PubMed&list_uids=,12964161,12930846,8861913,16616141,&dopt=Abstract) |  | Direct | | [DNA damage inducible transcript 1](http://www.hprd.org/interactions?hprd_id=00528&isoform_id=00528_1&isoform_name=Isoform_1) |  | [In Vivo ; In Vitro ; Yeast 2 Hybrid](http://www.ncbi.nlm.nih.gov/entrez/query.fcgi?cmd=Retrieve&db=PubMed&list_uids=,10828065,7973727,7478510,&dopt=Abstract) |  | Direct | | [DNA methyltransferase 1](http://www.hprd.org/interactions?hprd_id=00532&isoform_id=00532_1&isoform_name=Isoform_1) |  | [In Vitro](http://www.ncbi.nlm.nih.gov/entrez/query.fcgi?cmd=Retrieve&db=PubMed&list_uids=,9302295,12354094,&dopt=Abstract) |  | Direct | | [DNA polymerase, delta](http://www.hprd.org/interactions?hprd_id=08882&isoform_id=08882_1&isoform_name=Isoform_1) |  | [In Vivo ; In Vitro](http://www.ncbi.nlm.nih.gov/entrez/query.fcgi?cmd=Retrieve&db=PubMed&list_uids=,12171929,12403614,&dopt=Abstract) |  | Direct | | [ERCC5](http://www.hprd.org/interactions?hprd_id=00595&isoform_id=00595_1&isoform_name=Isoform_1) |  | [In Vivo ; In Vitro](http://www.ncbi.nlm.nih.gov/entrez/query.fcgi?cmd=Retrieve&db=PubMed&list_uids=,10408173,9305916,&dopt=Abstract) |  | Direct | | [Ku antigen, 70kDa](http://www.hprd.org/interactions?hprd_id=01071&isoform_id=01071_1&isoform_name=Isoform_1) |  | [In Vivo ; In Vitro](http://www.ncbi.nlm.nih.gov/entrez/query.fcgi?cmd=Retrieve&db=PubMed&list_uids=,11239001,12171929,&dopt=Abstract) |  | Direct | | [LIG1 DNA ligase I](http://www.hprd.org/interactions?hprd_id=00534&isoform_id=00534_1&isoform_name=Isoform_1) |  | [In Vitro](http://www.ncbi.nlm.nih.gov/entrez/query.fcgi?cmd=Retrieve&db=PubMed&list_uids=,12171929,10559261,&dopt=Abstract) |  | Direct | | [MSH2](http://www.hprd.org/interactions?hprd_id=00389&isoform_id=00389_1&isoform_name=Isoform_1) |  | [In Vitro](http://www.ncbi.nlm.nih.gov/entrez/query.fcgi?cmd=Retrieve&db=PubMed&list_uids=,12171929,8858149,&dopt=Abstract) |  | Direct | | [Replication factor C, subunit 3](http://www.hprd.org/interactions?hprd_id=02676&isoform_id=02676_1&isoform_name=Isoform_1) |  | [In Vivo ; In Vitro](http://www.ncbi.nlm.nih.gov/entrez/query.fcgi?cmd=Retrieve&db=PubMed&list_uids=,10051561,12171929,9092549,14657243,&dopt=Abstract) |  | Direct | | [Replication factor C1](http://www.hprd.org/interactions?hprd_id=00024&isoform_id=00024_1&isoform_name=Isoform_1) |  | [In Vivo ; In Vitro](http://www.ncbi.nlm.nih.gov/entrez/query.fcgi?cmd=Retrieve&db=PubMed&list_uids=,12045192,12171929,8861969,8999859,&dopt=Abstract) |  | Direct | | [Replication factor C4](http://www.hprd.org/interactions?hprd_id=00022&isoform_id=00022_1&isoform_name=Isoform_1) |  | [In Vivo ; In Vitro](http://www.ncbi.nlm.nih.gov/entrez/query.fcgi?cmd=Retrieve&db=PubMed&list_uids=,10051561,12171929,&dopt=Abstract) |  | Direct | | [Uracil DNA glycosylase](http://www.hprd.org/interactions?hprd_id=01881&isoform_id=01881_1&isoform_name=Isoform_1) |  | [In Vitro](http://www.ncbi.nlm.nih.gov/entrez/query.fcgi?cmd=Retrieve&db=PubMed&list_uids=,10393198,12171929,&dopt=Abstract) |  | Direct | | [YB-1](http://www.hprd.org/interactions?hprd_id=01095&isoform_id=01095_1&isoform_name=Isoform_1) |  | [In Vitro ; In Vivo](http://www.ncbi.nlm.nih.gov/entrez/query.fcgi?cmd=Retrieve&db=PubMed&list_uids=,9927044,&dopt=Abstract) |  | Direct | | [Proliferating cell nuclear antigen](http://www.hprd.org/interactions?hprd_id=01456&isoform_id=01456_1&isoform_name=Isoform_1) |  | [In Vitro](http://www.ncbi.nlm.nih.gov/entrez/query.fcgi?cmd=Retrieve&db=PubMed&list_uids=,8861913,7673244,&dopt=Abstract) |  | Direct | | [Replication factor A protein 1](http://www.hprd.org/interactions?hprd_id=01565&isoform_id=01565_1&isoform_name=Isoform_1) |  | [In Vitro](http://www.ncbi.nlm.nih.gov/entrez/query.fcgi?cmd=Retrieve&db=PubMed&list_uids=,12171929,&dopt=Abstract) |  | Direct | | [Prothymosin alpha](http://www.hprd.org/interactions?hprd_id=01778&isoform_id=01778_1&isoform_name=Isoform_1) |  | [In Vitro](http://www.ncbi.nlm.nih.gov/entrez/query.fcgi?cmd=Retrieve&db=PubMed&list_uids=,11310559,&dopt=Abstract) |  | Direct | | [XRCC1](http://www.hprd.org/interactions?hprd_id=01909&isoform_id=01909_1&isoform_name=Isoform_1) |  | [In Vivo ; In Vitro ; Yeast 2 Hybrid](http://www.ncbi.nlm.nih.gov/entrez/query.fcgi?cmd=Retrieve&db=PubMed&list_uids=,15107487,&dopt=Abstract) |  | Direct | | [Flap endonuclease 1](http://www.hprd.org/interactions?hprd_id=02670&isoform_id=02670_1&isoform_name=Isoform_1) |  | [In Vivo ; In Vitro ; Yeast 2 Hybrid](http://www.ncbi.nlm.nih.gov/entrez/query.fcgi?cmd=Retrieve&db=PubMed&list_uids=,9305916,1477073,11430825,9545252,8876181,7673186,14657243,&dopt=Abstract) |  | Direct | | [Replication factor C, subunit 2](http://www.hprd.org/interactions?hprd_id=02675&isoform_id=02675_1&isoform_name=Isoform_1) |  | [In Vitro](http://www.ncbi.nlm.nih.gov/entrez/query.fcgi?cmd=Retrieve&db=PubMed&list_uids=,8093561,&dopt=Abstract) |  | Direct | | [PC4](http://www.hprd.org/interactions?hprd_id=02737&isoform_id=02737_1&isoform_name=Isoform_1) |  | [Yeast 2 Hybrid](http://www.ncbi.nlm.nih.gov/entrez/query.fcgi?cmd=Retrieve&db=PubMed&list_uids=,11313979,&dopt=Abstract) |  | Direct | | [Cyclin dependent kinase inhibitor 1C](http://www.hprd.org/interactions?hprd_id=02913&isoform_id=02913_1&isoform_name=Isoform_1) |  | [In Vivo ; In Vitro](http://www.ncbi.nlm.nih.gov/entrez/query.fcgi?cmd=Retrieve&db=PubMed&list_uids=,9465025,&dopt=Abstract) |  | Direct | | [MutS homolog 3](http://www.hprd.org/interactions?hprd_id=02931&isoform_id=02931_1&isoform_name=Isoform_1) |  | [In Vivo ; In Vitro](http://www.ncbi.nlm.nih.gov/entrez/query.fcgi?cmd=Retrieve&db=PubMed&list_uids=,11274057,11005803,12171929,&dopt=Abstract) |  | Direct | | [DNA dependent protein kinase catalytic subunit](http://www.hprd.org/interactions?hprd_id=02941&isoform_id=02941_1&isoform_name=Isoform_1) |  | [In Vitro](http://www.ncbi.nlm.nih.gov/entrez/query.fcgi?cmd=Retrieve&db=PubMed&list_uids=,12171929,&dopt=Abstract) |  | Direct | | [Histone deacetylase 1](http://www.hprd.org/interactions?hprd_id=03143&isoform_id=03143_1&isoform_name=Isoform_1) |  | [In Vivo ; In Vitro](http://www.ncbi.nlm.nih.gov/entrez/query.fcgi?cmd=Retrieve&db=PubMed&list_uids=,11929879,&dopt=Abstract) |  | Direct | | [Chromatin assembly factor 1 subunit A](http://www.hprd.org/interactions?hprd_id=03148&isoform_id=03148_1&isoform_name=Isoform_1) |  | [In Vivo ; In Vitro ; Yeast 2 Hybrid](http://www.ncbi.nlm.nih.gov/entrez/query.fcgi?cmd=Retrieve&db=PubMed&list_uids=,10648606,16826239,&dopt=Abstract) |  | Direct | | [Inhibitor of growth 1](http://www.hprd.org/interactions?hprd_id=03337&isoform_id=03337_1&isoform_name=Isoform_1) |  | [In Vivo ; In Vitro](http://www.ncbi.nlm.nih.gov/entrez/query.fcgi?cmd=Retrieve&db=PubMed&list_uids=,11682605,12015309,&dopt=Abstract) |  | Direct | | [CDC6](http://www.hprd.org/interactions?hprd_id=04022&isoform_id=04022_1&isoform_name=Isoform_1) |  | [Yeast 2 Hybrid](http://www.ncbi.nlm.nih.gov/entrez/query.fcgi?cmd=Retrieve&db=PubMed&list_uids=,9566895,&dopt=Abstract) |  | Direct | | [E1A binding protein p300](http://www.hprd.org/interactions?hprd_id=04078&isoform_id=04078_1&isoform_name=Isoform_1) |  | [In Vivo ; In Vitro](http://www.ncbi.nlm.nih.gov/entrez/query.fcgi?cmd=Retrieve&db=PubMed&list_uids=,11268218,&dopt=Abstract) |  | Direct | | [ATP-dependent RNA helicase A](http://www.hprd.org/interactions?hprd_id=04386&isoform_id=04386_1&isoform_name=Isoform_1) |  | [In Vitro](http://www.ncbi.nlm.nih.gov/entrez/query.fcgi?cmd=Retrieve&db=PubMed&list_uids=,12171929,&dopt=Abstract) |  | Direct | | [Cyclin dependent kinase 6](http://www.hprd.org/interactions?hprd_id=04533&isoform_id=04533_1&isoform_name=Isoform_1) |  | [In Vivo](http://www.ncbi.nlm.nih.gov/entrez/query.fcgi?cmd=Retrieve&db=PubMed&list_uids=,9667749,&dopt=Abstract) |  | Direct | | [HUS1](http://www.hprd.org/interactions?hprd_id=04787&isoform_id=04787_1&isoform_name=Isoform_1) |  | [Yeast 2 Hybrid](http://www.ncbi.nlm.nih.gov/entrez/query.fcgi?cmd=Retrieve&db=PubMed&list_uids=,11077446,&dopt=Abstract) |  | Direct | | [RAD9](http://www.hprd.org/interactions?hprd_id=04788&isoform_id=04788_1&isoform_name=Isoform_1) |  | [In Vitro](http://www.ncbi.nlm.nih.gov/entrez/query.fcgi?cmd=Retrieve&db=PubMed&list_uids=,11994305,&dopt=Abstract) |  | Direct | | [DNA polymerase eta](http://www.hprd.org/interactions?hprd_id=04913&isoform_id=04913_1&isoform_name=Isoform_1) |  | [In Vitro ; Yeast 2 Hybrid](http://www.ncbi.nlm.nih.gov/entrez/query.fcgi?cmd=Retrieve&db=PubMed&list_uids=,11585903, 16763556,&dopt=Abstract) |  | Direct | | [RecQ protein like 2](http://www.hprd.org/interactions?hprd_id=05212&isoform_id=05212_1&isoform_name=Isoform_1) |  | [In Vivo ; In Vitro](http://www.ncbi.nlm.nih.gov/entrez/query.fcgi?cmd=Retrieve&db=PubMed&list_uids=,10871373,12633936,&dopt=Abstract) |  | Direct | | [P160](http://www.hprd.org/interactions?hprd_id=05349&isoform_id=05349_1&isoform_name=Isoform_1) |  | [In Vitro](http://www.ncbi.nlm.nih.gov/entrez/query.fcgi?cmd=Retrieve&db=PubMed&list_uids=,12171929,&dopt=Abstract) |  | Direct | | [MYH](http://www.hprd.org/interactions?hprd_id=05380&isoform_id=05380_1&isoform_name=Isoform_1) |  | [In Vivo ; In Vitro](http://www.ncbi.nlm.nih.gov/entrez/query.fcgi?cmd=Retrieve&db=PubMed&list_uids=,11092888,&dopt=Abstract) |  | Direct | | [GADD45 beta](http://www.hprd.org/interactions?hprd_id=05382&isoform_id=05382_1&isoform_name=Isoform_1) |  | [In Vivo ; In Vitro ; Yeast 2 Hybrid](http://www.ncbi.nlm.nih.gov/entrez/query.fcgi?cmd=Retrieve&db=PubMed&list_uids=,10828065,&dopt=Abstract) |  | Direct | | [GADD45 gamma](http://www.hprd.org/interactions?hprd_id=05383&isoform_id=05383_1&isoform_name=Isoform_1) |  | [In Vivo ; In Vitro ; Yeast 2 Hybrid](http://www.ncbi.nlm.nih.gov/entrez/query.fcgi?cmd=Retrieve&db=PubMed&list_uids=,11022036,10455148,&dopt=Abstract) |  | Direct | | [Uracil DNA glycosylase 2](http://www.hprd.org/interactions?hprd_id=06375&isoform_id=06375_1&isoform_name=Isoform_1) |  | [In Vitro ; Yeast 2 Hybrid](http://www.ncbi.nlm.nih.gov/entrez/query.fcgi?cmd=Retrieve&db=PubMed&list_uids=,10393198,12171929,&dopt=Abstract) |  | Direct | | [APEX2](http://www.hprd.org/interactions?hprd_id=06442&isoform_id=06442_1&isoform_name=Isoform_1) |  | [In Vivo ; In Vitro](http://www.ncbi.nlm.nih.gov/entrez/query.fcgi?cmd=Retrieve&db=PubMed&list_uids=,11376153,&dopt=Abstract) |  | Direct | | [DNA polymerase epsilon, catalytic subunit A](http://www.hprd.org/interactions?hprd_id=07177&isoform_id=07177_1&isoform_name=Isoform_1) |  | [In Vitro](http://www.ncbi.nlm.nih.gov/entrez/query.fcgi?cmd=Retrieve&db=PubMed&list_uids=,12171929,&dopt=Abstract) |  | Direct | | [MSH6](http://www.hprd.org/interactions?hprd_id=07202&isoform_id=07202_1&isoform_name=Isoform_1) |  | [In Vivo ; In Vitro](http://www.ncbi.nlm.nih.gov/entrez/query.fcgi?cmd=Retrieve&db=PubMed&list_uids=,11005803,12171929,11274057,&dopt=Abstract) |  | Direct | | [DNA polymerase beta](http://www.hprd.org/interactions?hprd_id=07517&isoform_id=07517_1&isoform_name=Isoform_1) |  | [In Vivo ; Yeast 2 Hybrid](http://www.ncbi.nlm.nih.gov/entrez/query.fcgi?cmd=Retrieve&db=PubMed&list_uids=,12063248,&dopt=Abstract) |  | Direct | | [Myeloid cell leukemia 1](http://www.hprd.org/interactions?hprd_id=08870&isoform_id=08870_1&isoform_name=Isoform_1) |  | [In Vivo ; In Vitro ; Yeast 2 Hybrid](http://www.ncbi.nlm.nih.gov/entrez/query.fcgi?cmd=Retrieve&db=PubMed&list_uids=,10978339,&dopt=Abstract) |  | Direct | | [DNA nucleotidylexotransferase](http://www.hprd.org/interactions?hprd_id=08925&isoform_id=08925_1&isoform_name=Isoform_1) |  | [In Vivo ; In Vitro ; Yeast 2 Hybrid](http://www.ncbi.nlm.nih.gov/entrez/query.fcgi?cmd=Retrieve&db=PubMed&list_uids=,11554927,&dopt=Abstract) |  | Direct | | [Ku antigen, 80kDa](http://www.hprd.org/interactions?hprd_id=08935&isoform_id=08935_1&isoform_name=Isoform_1) |  | [In Vitro ; In Vivo](http://www.ncbi.nlm.nih.gov/entrez/query.fcgi?cmd=Retrieve&db=PubMed&list_uids=,12171929,12393188,&dopt=Abstract) |  | Direct | | [DNA polymerase lambda](http://www.hprd.org/interactions?hprd_id=09394&isoform_id=09394_1&isoform_name=Isoform_1) |  | [In Vitro ; In Vivo](http://www.ncbi.nlm.nih.gov/entrez/query.fcgi?cmd=Retrieve&db=PubMed&list_uids=,12368291,12081642,11784855,16174846,&dopt=Abstract) |  | Direct | | [Estrogen receptor binding protein](http://www.hprd.org/interactions?hprd_id=09994&isoform_id=09994_1&isoform_name=Isoform_1) |  | [In Vitro](http://www.ncbi.nlm.nih.gov/entrez/query.fcgi?cmd=Retrieve&db=PubMed&list_uids=,12786946,&dopt=Abstract) |  | Direct | | [DNA polymerase delta subunit 3](http://www.hprd.org/interactions?hprd_id=11446&isoform_id=11446_1&isoform_name=Isoform_1) |  | [In Vitro ; Yeast 2 Hybrid](http://www.ncbi.nlm.nih.gov/entrez/query.fcgi?cmd=Retrieve&db=PubMed&list_uids=,11595739,12171929,10219083, 16763556,&dopt=Abstract) |  | Direct | | [Potassium channel tetramerisation domain containing 13](http://www.hprd.org/interactions?hprd_id=12339&isoform_id=12339_1&isoform_name=Isoform_1) |  | [In Vivo ; In Vitro](http://www.ncbi.nlm.nih.gov/entrez/query.fcgi?cmd=Retrieve&db=PubMed&list_uids=,11593007,&dopt=Abstract) |  | Direct | | [CHL12](http://www.hprd.org/interactions?hprd_id=13059&isoform_id=13059_1&isoform_name=Isoform_1) |  | [In Vitro ; In Vivo](http://www.ncbi.nlm.nih.gov/entrez/query.fcgi?cmd=Retrieve&db=PubMed&list_uids=,12171929,12766176,&dopt=Abstract) |  | Direct | | [Defective in sister chromatid cohesion homolog 1](http://www.hprd.org/interactions?hprd_id=13123&isoform_id=13123_1&isoform_name=Isoform_1) |  | [In Vivo](http://www.ncbi.nlm.nih.gov/entrez/query.fcgi?cmd=Retrieve&db=PubMed&list_uids=,12766176,&dopt=Abstract) |  | Direct | | [DNA polymerase delta subunit 2](http://www.hprd.org/interactions?hprd_id=15982&isoform_id=15982_1&isoform_name=Isoform_1) |  | [In Vivo ; In Vitro](http://www.ncbi.nlm.nih.gov/entrez/query.fcgi?cmd=Retrieve&db=PubMed&list_uids=,11986310,12171929,&dopt=Abstract) |  | Direct | | [DNA directed polymerase, mu](http://www.hprd.org/interactions?hprd_id=16208&isoform_id=16208_1&isoform_name=Isoform_1) |  | [In Vitro](http://www.ncbi.nlm.nih.gov/entrez/query.fcgi?cmd=Retrieve&db=PubMed&list_uids=,11724965,&dopt=Abstract) |  | Direct | | [DERPC](http://www.hprd.org/interactions?hprd_id=16797&isoform_id=16797_1&isoform_name=Isoform_1) |  | [In Vivo](http://www.ncbi.nlm.nih.gov/entrez/query.fcgi?cmd=Retrieve&db=PubMed&list_uids=,12766176,&dopt=Abstract) |  | Direct | | [Polymerase delta interacting protein 2](http://www.hprd.org/interactions?hprd_id=17874&isoform_id=17874_1&isoform_name=Isoform_1) |  | [In Vitro ; In Vivo ; Yeast 2 Hybrid](http://www.ncbi.nlm.nih.gov/entrez/query.fcgi?cmd=Retrieve&db=PubMed&list_uids=,12522211,&dopt=Abstract) |  | Direct | | [Replication factor C (activator 1) 5, 36.5kDa](http://www.hprd.org/interactions?hprd_id=02677&isoform_id=02677_1&isoform_name=Isoform_1) |  | [In Vitro](http://www.ncbi.nlm.nih.gov/entrez/query.fcgi?cmd=Retrieve&db=PubMed&list_uids=,8999859,&dopt=Abstract) |  | Direct | | [Bromodomain adjacent to zinc finger domain, 1B](http://www.hprd.org/interactions?hprd_id=10416&isoform_id=10416_1&isoform_name=Isoform_1) |  | [In Vitro](http://www.ncbi.nlm.nih.gov/entrez/query.fcgi?cmd=Retrieve&db=PubMed&list_uids=,15543136,&dopt=Abstract) |  | Direct | | [Treacle](http://www.hprd.org/interactions?hprd_id=06026&isoform_id=06026_1&isoform_name=Isoform_1) |  | [In Vitro](http://www.ncbi.nlm.nih.gov/entrez/query.fcgi?cmd=Retrieve&db=PubMed&list_uids=,12171929,&dopt=Abstract) |  | Direct | | [DNA polymerase delta subunit 4](http://www.hprd.org/interactions?hprd_id=17873&isoform_id=17873_1&isoform_name=Isoform_1) |  | [In Vitro ; Yeast 2 Hybrid](http://www.ncbi.nlm.nih.gov/entrez/query.fcgi?cmd=Retrieve&db=PubMed&list_uids=,16510448,&dopt=Abstract) |  | Direct | | [SEC23 interacting protein](http://www.hprd.org/interactions?hprd_id=07152&isoform_id=07152_1&isoform_name=Isoform_1) |  | [In Vitro](http://www.ncbi.nlm.nih.gov/entrez/query.fcgi?cmd=Retrieve&db=PubMed&list_uids=,16510448,&dopt=Abstract) |  | Direct | | [Ubiquitin B](http://www.hprd.org/interactions?hprd_id=06771&isoform_id=06771_1&isoform_name=Isoform_1) |  | [In Vivo](http://www.ncbi.nlm.nih.gov/entrez/query.fcgi?cmd=Retrieve&db=PubMed&list_uids=,15149598,&dopt=Abstract) |  | Direct | | [DUP](http://www.hprd.org/interactions?hprd_id=06900&isoform_id=06900_1&isoform_name=Isoform_1) |  | [In Vivo ; In Vitro](http://www.ncbi.nlm.nih.gov/entrez/query.fcgi?cmd=Retrieve&db=PubMed&list_uids=,16407252,16482215,&dopt=Abstract) |  | Direct | | [Protein phosphatase 1, catalytic subunit, alpha isoform](http://www.hprd.org/interactions?hprd_id=15942&isoform_id=15942_1&isoform_name=Isoform_1) |  | [In Vivo ; In Vitro](http://www.ncbi.nlm.nih.gov/entrez/query.fcgi?cmd=Retrieve&db=PubMed&list_uids=,17274640,&dopt=Abstract) |  | Direct | | [DNA polymerase iota](http://www.hprd.org/interactions?hprd_id=16094&isoform_id=16094_1&isoform_name=Isoform_1) |  | [In Vitro ; Yeast 2 Hybrid](http://www.ncbi.nlm.nih.gov/entrez/query.fcgi?cmd=Retrieve&db=PubMed&list_uids=,16763556,&dopt=Abstract) |  | Direct | | [CTIP](http://www.hprd.org/interactions?hprd_id=04990&isoform_id=04990_1&isoform_name=Isoform_1) |  | [Yeast 2 Hybrid ; In Vivo ; In Vitro](http://www.ncbi.nlm.nih.gov/entrez/query.fcgi?cmd=Retrieve&db=PubMed&list_uids=19342888&dopt=Abstract) |  | Direct | | [Cyclin dependent kinase inhibitor 1A](http://www.hprd.org/interactions?hprd_id=00298&isoform_id=00298_1&isoform_name=Isoform_1) [CDC2](http://www.hprd.org/interactions?hprd_id=00302&isoform_id=00302_1&isoform_name=Isoform_1) [Cyclin B1](http://www.hprd.org/interactions?hprd_id=00454&isoform_id=00454_1&isoform_name=Isoform_1) |  | [In Vitro ; In Vivo](http://www.ncbi.nlm.nih.gov/entrez/query.fcgi?cmd=Retrieve&db=PubMed&list_uids=11559705&dopt=Abstract) |  | Complex | | [Cyclin dependent kinase inhibitor 1A](http://www.hprd.org/interactions?hprd_id=00298&isoform_id=00298_1&isoform_name=Isoform_1) [Cyclin dependent kinase 2](http://www.hprd.org/interactions?hprd_id=00310&isoform_id=00310_1&isoform_name=Isoform_1) [Cyclin D1](http://www.hprd.org/interactions?hprd_id=01346&isoform_id=01346_1&isoform_name=Isoform_1) |  | [In Vivo](http://www.ncbi.nlm.nih.gov/entrez/query.fcgi?cmd=Retrieve&db=PubMed&list_uids=8101826&dopt=Abstract) |  | Complex | | [Cyclin dependent kinase inhibitor 1A](http://www.hprd.org/interactions?hprd_id=00298&isoform_id=00298_1&isoform_name=Isoform_1) [Cyclin dependent kinase 4](http://www.hprd.org/interactions?hprd_id=00447&isoform_id=00447_1&isoform_name=Isoform_1) [Cyclin dependent kinase 5](http://www.hprd.org/interactions?hprd_id=00449&isoform_id=00449_1&isoform_name=Isoform_1) [DNA topoisomerase I](http://www.hprd.org/interactions?hprd_id=00535&isoform_id=00535_1&isoform_name=Isoform_1) [Replication factor A protein 1](http://www.hprd.org/interactions?hprd_id=01565&isoform_id=01565_1&isoform_name=Isoform_1) [ATP-dependent RNA helicase A](http://www.hprd.org/interactions?hprd_id=04386&isoform_id=04386_1&isoform_name=Isoform_1) |  | [In Vivo ; In Vitro](http://www.ncbi.nlm.nih.gov/entrez/query.fcgi?cmd=Retrieve&db=PubMed&list_uids=11254741,9396813&dopt=Abstract) |  | Complex | | [RAD18](http://www.hprd.org/interactions?hprd_id=09242&isoform_id=09242_1&isoform_name=Isoform_1) [Ubiquitin conjugating enzyme E2B](http://www.hprd.org/interactions?hprd_id=01533&isoform_id=01533_1&isoform_name=Isoform_1) |  | [In Vitro](http://www.ncbi.nlm.nih.gov/entrez/query.fcgi?cmd=Retrieve&db=PubMed&list_uids=15359278&dopt=Abstract) |  | Complex | | [DNA Polymerase, alpha](http://www.hprd.org/interactions?hprd_id=02416&isoform_id=02416_1&isoform_name=Isoform_1) [DNA polymerase, delta](http://www.hprd.org/interactions?hprd_id=08882&isoform_id=08882_1&isoform_name=Isoform_1) [DNA primase large subunit](http://www.hprd.org/interactions?hprd_id=08900&isoform_id=08900_1&isoform_name=Isoform_1) [Replication factor C1](http://www.hprd.org/interactions?hprd_id=00024&isoform_id=00024_1&isoform_name=Isoform_1) |  | [In Vivo ; In Vitro](http://www.ncbi.nlm.nih.gov/entrez/query.fcgi?cmd=Retrieve&db=PubMed&list_uids=9563011,12220650&dopt=Abstract) |  | Complex | | [Replication factor C (activator 1) 5, 36.5kDa](http://www.hprd.org/interactions?hprd_id=02677&isoform_id=02677_1&isoform_name=Isoform_1) [Replication factor C4](http://www.hprd.org/interactions?hprd_id=00022&isoform_id=00022_1&isoform_name=Isoform_1) [Replication factor C, subunit 2](http://www.hprd.org/interactions?hprd_id=02675&isoform_id=02675_1&isoform_name=Isoform_1) [Replication factor C, subunit 3](http://www.hprd.org/interactions?hprd_id=02676&isoform_id=02676_1&isoform_name=Isoform_1) |  | [In Vivo ; In Vitro](http://www.ncbi.nlm.nih.gov/entrez/query.fcgi?cmd=Retrieve&db=PubMed&list_uids=9228079,12171929&dopt=Abstract) |  | Complex | | [Ubiquitin B](http://www.hprd.org/interactions?hprd_id=06771&isoform_id=06771_1&isoform_name=Isoform_1) [DNA polymerase eta](http://www.hprd.org/interactions?hprd_id=04913&isoform_id=04913_1&isoform_name=Isoform_1) DNA |  | [In Vivo](http://www.ncbi.nlm.nih.gov/entrez/query.fcgi?cmd=Retrieve&db=PubMed&list_uids=15149598&dopt=Abstract) |  | Complex | | [Ubiquitin conjugating enzyme E2B](http://www.hprd.org/interactions?hprd_id=01533&isoform_id=01533_1&isoform_name=Isoform_1) [RAD18](http://www.hprd.org/interactions?hprd_id=09242&isoform_id=09242_1&isoform_name=Isoform_1) |  | [In Vitro](http://www.ncbi.nlm.nih.gov/entrez/query.fcgi?cmd=Retrieve&db=PubMed&list_uids=17720710&dopt=Abstract) |  | Complex | | [DNA polymerase, delta](http://www.hprd.org/interactions?hprd_id=08882&isoform_id=08882_1&isoform_name=Isoform_1) [Chromatin assembly factor 1 subunit A](http://www.hprd.org/interactions?hprd_id=03148&isoform_id=03148_1&isoform_name=Isoform_1) |  | [In Vivo](http://www.ncbi.nlm.nih.gov/entrez/query.fcgi?cmd=Retrieve&db=PubMed&list_uids=15805117&dopt=Abstract) |  | Complex | | [SWI/SNF related matrix associated actin dependent regulator of chromatin subfamily a member 3](http://www.hprd.org/interactions?hprd_id=04461&isoform_id=04461_1&isoform_name=Isoform_1) [Ubiquitin conjugating enzyme E2B](http://www.hprd.org/interactions?hprd_id=01533&isoform_id=01533_1&isoform_name=Isoform_1) [RAD18](http://www.hprd.org/interactions?hprd_id=09242&isoform_id=09242_1&isoform_name=Isoform_1) [Ubiquitin conjugating enzyme E2 variant 2](http://www.hprd.org/interactions?hprd_id=04300&isoform_id=04300_1&isoform_name=Isoform_1) [Ubiquitin conjugating enzyme E2N](http://www.hprd.org/interactions?hprd_id=04725&isoform_id=04725_1&isoform_name=Isoform_1) |  | [In Vivo ; In Vitro](http://www.ncbi.nlm.nih.gov/entrez/query.fcgi?cmd=Retrieve&db=PubMed&list_uids=,18316726,&dopt=Abstract) |  | Complex | | [Claspin](http://www.hprd.org/interactions?hprd_id=09257&isoform_id=09257_1&isoform_name=Isoform_1) [TIM1](http://www.hprd.org/interactions?hprd_id=04864&isoform_id=04864_1&isoform_name=Isoform_1) |  | [In Vivo](http://www.ncbi.nlm.nih.gov/entrez/query.fcgi?cmd=Retrieve&db=PubMed&list_uids=,18451105,&dopt=Abstract) |  | Complex | | |  | |  | | --- | --- | --- | --- | --- | --- | --- | --- | --- | --- | --- | --- | --- | --- | --- | --- | --- | --- | --- | --- | --- | --- | --- | --- | --- | --- | --- | --- | --- | --- | --- | --- | --- | --- | --- | --- | --- | --- | --- | --- | --- | --- | --- | --- | --- | --- | --- | --- | --- | --- | --- | --- | --- | --- | --- | --- | --- | --- | --- | --- | --- | --- | --- | --- | --- | --- | --- | --- | --- | --- | --- | --- | --- | --- | --- | --- | --- | --- | --- | --- | --- | --- | --- | --- | --- | --- | --- | --- | --- | --- | --- | --- | --- | --- | --- | --- | --- | --- | --- | --- | --- | --- | --- | --- | --- | --- | --- | --- | --- | --- | --- | --- | --- | --- | --- | --- | --- | --- | --- | --- | --- | --- | --- | --- | --- | --- | --- | --- | --- | --- | --- | --- | --- | --- | --- | --- | --- | --- | --- | --- | --- | --- | --- | --- | --- | --- | --- | --- | --- | --- | --- | --- | --- | --- | --- | --- | --- | --- | --- | --- | --- | --- | --- | --- | --- | --- | --- | --- | --- | --- | --- | --- | --- | --- | --- | --- | --- | --- | --- | --- | --- | --- | --- | --- | --- | --- | --- | --- | --- | --- | --- | --- | --- | --- | --- | --- | --- | --- | --- | --- | --- | --- | --- | --- | --- | --- | --- | --- | --- | --- | --- | --- | --- | --- | --- | --- | --- | --- | --- | --- | --- | --- | --- | --- | --- | --- | --- | --- | --- | --- | --- | --- | --- | --- | --- | --- | --- | --- | --- | --- | --- | --- | --- | --- | --- | --- | --- | --- | --- | --- | --- | --- | --- | --- | --- | --- | --- | --- | --- | --- | --- | --- | --- | --- | --- | --- | --- | --- | --- | --- | --- | --- | --- | --- | --- | --- | --- | --- | --- | --- | --- | --- | --- | --- | --- | --- | --- | --- | --- | --- | --- | --- | --- | --- | --- | --- | --- | --- | --- | --- | --- | --- | --- | --- | --- | --- | --- | --- | --- | --- | --- | --- | --- | --- | --- | --- | --- | --- | --- | --- | --- | --- | --- | --- | --- | --- | --- | --- | --- | --- | --- | --- | --- | --- | --- | --- | --- | --- | --- | --- | --- | --- | --- | --- | --- | --- | --- | --- | --- | --- | --- | --- | --- | --- | --- | --- | --- | --- | --- | --- | --- | --- | --- | --- | --- | --- | --- | --- | --- | --- | --- | --- | --- | --- | --- | --- | --- | --- | --- | --- | --- | --- | --- | --- | --- | --- | --- | --- | --- | --- | --- | --- | --- | --- | --- | --- | --- | --- | --- | --- | --- | --- | --- | --- | --- | --- | --- | --- | --- | --- | --- | --- | --- | --- | --- | --- | --- | --- | --- | --- | --- | --- | --- | --- | --- | --- | --- | --- | --- | --- | --- | --- | --- | --- | --- | --- | --- | --- | --- | --- | --- | --- | --- | --- | --- | --- | |  |  |  | |
| --- | --- | --- | --- | --- | --- | --- | --- | --- | --- | --- | --- | --- | --- | --- | --- | --- | --- | --- | --- | --- | --- | --- | --- | --- | --- | --- | --- | --- | --- | --- | --- | --- | --- | --- | --- | --- | --- | --- | --- | --- | --- | --- | --- | --- | --- | --- | --- | --- | --- | --- | --- | --- | --- | --- | --- | --- | --- | --- | --- | --- | --- | --- | --- | --- | --- | --- | --- | --- | --- | --- | --- | --- | --- | --- | --- | --- | --- | --- | --- | --- | --- | --- | --- | --- | --- | --- | --- | --- | --- | --- | --- | --- | --- | --- | --- | --- | --- | --- | --- | --- | --- | --- | --- | --- | --- | --- | --- | --- | --- | --- | --- | --- | --- | --- | --- | --- | --- | --- | --- | --- | --- | --- | --- | --- | --- | --- | --- | --- | --- | --- | --- | --- | --- | --- | --- | --- | --- | --- | --- | --- | --- | --- | --- | --- | --- | --- | --- | --- | --- | --- | --- | --- | --- | --- | --- | --- | --- | --- | --- | --- | --- | --- | --- | --- | --- | --- | --- | --- | --- | --- | --- | --- | --- | --- | --- | --- | --- | --- | --- | --- | --- | --- | --- | --- | --- | --- | --- | --- | --- | --- | --- | --- | --- | --- | --- | --- | --- | --- | --- | --- | --- | --- | --- | --- | --- | --- | --- | --- | --- | --- | --- | --- | --- | --- | --- | --- | --- | --- | --- | --- | --- | --- | --- | --- | --- | --- | --- | --- | --- | --- | --- | --- | --- | --- | --- | --- | --- | --- | --- | --- | --- | --- | --- | --- | --- | --- | --- | --- | --- | --- | --- | --- | --- | --- | --- | --- | --- | --- | --- | --- | --- | --- | --- | --- | --- | --- | --- | --- | --- | --- | --- | --- | --- | --- | --- | --- | --- | --- | --- | --- | --- | --- | --- | --- | --- | --- | --- | --- | --- | --- | --- | --- | --- | --- | --- | --- | --- | --- | --- | --- | --- | --- | --- | --- | --- | --- | --- | --- | --- | --- | --- | --- | --- | --- | --- | --- | --- | --- | --- | --- | --- | --- | --- | --- | --- | --- | --- | --- | --- | --- | --- | --- | --- | --- | --- | --- | --- | --- | --- | --- | --- | --- | --- | --- | --- | --- | --- | --- | --- | --- | --- | --- | --- | --- | --- | --- | --- | --- | --- | --- | --- | --- | --- | --- | --- | --- | --- | --- | --- | --- | --- | --- | --- | --- | --- | --- | --- | --- | --- | --- | --- | --- | --- | --- | --- | --- | --- | --- | --- | --- | --- | --- | --- | --- | --- | --- | --- | --- | --- | --- | --- | --- | --- | --- | --- | --- | --- | --- | --- | --- | --- | --- | --- | --- | --- | --- | --- | --- | --- | --- | --- | --- | --- | --- | --- | --- | --- | --- | --- | --- | --- | --- | --- | --- | --- | --- | --- | --- | --- | --- | --- | --- | --- | --- | --- | --- | --- | --- | --- | --- | --- | --- | --- | --- |

**CDK1**

|  | | 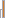 | **Protein Interactions** | 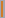 |  | | --- | --- | --- | --- |  |  | |  | | --- | | | **PROTEIN INTERACTORS** |  |  |  |  | | --- | --- | --- | --- | --- | | **Name of Interactor** |  | **Experiment Type** |  | **Type** | | [ABL](http://www.hprd.org/interactions?hprd_id=01809&isoform_id=01809_1&isoform_name=Isoform_1) |  | [In Vivo ; In Vitro](http://www.ncbi.nlm.nih.gov/entrez/query.fcgi?cmd=Retrieve&db=PubMed&list_uids=,2183353,&dopt=Abstract) |  | Direct | | [Amphiphysin](http://www.hprd.org/interactions?hprd_id=02687&isoform_id=02687_1&isoform_name=Isoform_1) |  | [In Vitro](http://www.ncbi.nlm.nih.gov/entrez/query.fcgi?cmd=Retrieve&db=PubMed&list_uids=,11113134,&dopt=Abstract) |  | Direct | | [Amyloid precursor like protein 2](http://www.hprd.org/interactions?hprd_id=00103&isoform_id=00103_1&isoform_name=Isoform_1) |  | [In Vivo ; In Vitro](http://www.ncbi.nlm.nih.gov/entrez/query.fcgi?cmd=Retrieve&db=PubMed&list_uids=,9109675,&dopt=Abstract) |  | Direct | | [Androgen receptor](http://www.hprd.org/interactions?hprd_id=02437&isoform_id=02437_1&isoform_name=Isoform_1) |  | [In Vivo ; In Vitro ; Yeast 2 Hybrid](http://www.ncbi.nlm.nih.gov/entrez/query.fcgi?cmd=Retrieve&db=PubMed&list_uids=,12569365,9725910,&dopt=Abstract) |  | Direct | | [BRCA1](http://www.hprd.org/interactions?hprd_id=00218&isoform_id=00218_1&isoform_name=Isoform_1) |  | [In Vivo ; In Vitro](http://www.ncbi.nlm.nih.gov/entrez/query.fcgi?cmd=Retrieve&db=PubMed&list_uids=,9244350,&dopt=Abstract) |  | Direct | | [BUB1](http://www.hprd.org/interactions?hprd_id=03907&isoform_id=03907_1&isoform_name=Isoform_1) |  | [In Vitro](http://www.ncbi.nlm.nih.gov/entrez/query.fcgi?cmd=Retrieve&db=PubMed&list_uids=,16760428,&dopt=Abstract) |  | Direct | | [Caldesmon 1](http://www.hprd.org/interactions?hprd_id=00251&isoform_id=00251_1&isoform_name=Isoform_1) |  | [In Vitro](http://www.ncbi.nlm.nih.gov/entrez/query.fcgi?cmd=Retrieve&db=PubMed&list_uids=,12613668,&dopt=Abstract) |  | Direct | | [cAMP response element modulator](http://www.hprd.org/interactions?hprd_id=00444&isoform_id=00444_1&isoform_name=Isoform_1) |  | [In Vivo ; In Vitro](http://www.ncbi.nlm.nih.gov/entrez/query.fcgi?cmd=Retrieve&db=PubMed&list_uids=,8404858,&dopt=Abstract) |  | Direct | | [Casein kinase II, alpha 1](http://www.hprd.org/interactions?hprd_id=00277&isoform_id=00277_1&isoform_name=Isoform_1) |  | [In Vitro ; In Vivo](http://www.ncbi.nlm.nih.gov/entrez/query.fcgi?cmd=Retrieve&db=PubMed&list_uids=,7592773,1400350,&dopt=Abstract) |  | Direct | | [Casein kinase II, beta](http://www.hprd.org/interactions?hprd_id=00278&isoform_id=00278_1&isoform_name=Isoform_1) |  | [In Vivo ; In Vitro](http://www.ncbi.nlm.nih.gov/entrez/query.fcgi?cmd=Retrieve&db=PubMed&list_uids=,7578274,&dopt=Abstract) |  | Direct | | [CDC 25A](http://www.hprd.org/interactions?hprd_id=00305&isoform_id=00305_1&isoform_name=Isoform_1) |  | [In Vivo ; In Vitro](http://www.ncbi.nlm.nih.gov/entrez/query.fcgi?cmd=Retrieve&db=PubMed&list_uids=,12411508,&dopt=Abstract) |  | Direct | | [Cdc2 associated protein CKS2](http://www.hprd.org/interactions?hprd_id=00300&isoform_id=00300_1&isoform_name=Isoform_1) |  | [In Vitro](http://www.ncbi.nlm.nih.gov/entrez/query.fcgi?cmd=Retrieve&db=PubMed&list_uids=,2227411,&dopt=Abstract) |  | Direct | | [Chromosome condensation 1](http://www.hprd.org/interactions?hprd_id=01559&isoform_id=01559_1&isoform_name=Isoform_1) |  | [In Vitro](http://www.ncbi.nlm.nih.gov/entrez/query.fcgi?cmd=Retrieve&db=PubMed&list_uids=,15014043,&dopt=Abstract) |  | Direct | | [Cut like 1](http://www.hprd.org/interactions?hprd_id=00295&isoform_id=00295_1&isoform_name=Isoform_1) |  | [In Vitro](http://www.ncbi.nlm.nih.gov/entrez/query.fcgi?cmd=Retrieve&db=PubMed&list_uids=,11584018,&dopt=Abstract) |  | Direct | | [Cyclin dependent kinase inhibitor 1A](http://www.hprd.org/interactions?hprd_id=00298&isoform_id=00298_1&isoform_name=Isoform_1) |  | [In Vivo](http://www.ncbi.nlm.nih.gov/entrez/query.fcgi?cmd=Retrieve&db=PubMed&list_uids=,9467962,&dopt=Abstract) |  | Direct | | [DNA replication licensing factor MCM4](http://www.hprd.org/interactions?hprd_id=09094&isoform_id=09094_1&isoform_name=Isoform_1) |  | [In Vivo](http://www.ncbi.nlm.nih.gov/entrez/query.fcgi?cmd=Retrieve&db=PubMed&list_uids=,16519687,&dopt=Abstract) |  | Direct | | [DNA topoisomerase II alpha](http://www.hprd.org/interactions?hprd_id=00536&isoform_id=00536_1&isoform_name=Isoform_1) |  | [In Vivo ; In Vitro](http://www.ncbi.nlm.nih.gov/entrez/query.fcgi?cmd=Retrieve&db=PubMed&list_uids=,7635160,12569090,&dopt=Abstract) |  | Direct | | [dUTP pyrophosphatase](http://www.hprd.org/interactions?hprd_id=03165&isoform_id=03165_1&isoform_name=Isoform_1) |  | [In Vivo ; In Vitro](http://www.ncbi.nlm.nih.gov/entrez/query.fcgi?cmd=Retrieve&db=PubMed&list_uids=,8631817,&dopt=Abstract) |  | Direct | | [E2F transcription factor 1](http://www.hprd.org/interactions?hprd_id=01806&isoform_id=01806_1&isoform_name=Isoform_1) |  | [In Vivo ; In Vitro](http://www.ncbi.nlm.nih.gov/entrez/query.fcgi?cmd=Retrieve&db=PubMed&list_uids=,8087847,&dopt=Abstract) |  | Direct | | [Epithelial cell transforming sequence 2 oncogene](http://www.hprd.org/interactions?hprd_id=11860&isoform_id=11860_1&isoform_name=Isoform_1) |  | [In Vitro](http://www.ncbi.nlm.nih.gov/entrez/query.fcgi?cmd=Retrieve&db=PubMed&list_uids=,16247472,&dopt=Abstract) |  | Direct | | [Epsin 1](http://www.hprd.org/interactions?hprd_id=06270&isoform_id=06270_1&isoform_name=Isoform_1) |  | [In Vitro](http://www.ncbi.nlm.nih.gov/entrez/query.fcgi?cmd=Retrieve&db=PubMed&list_uids=,10764745,&dopt=Abstract) |  | Direct | | [Eukaryotic translation elongation factor 1,delta](http://www.hprd.org/interactions?hprd_id=00560&isoform_id=00560_1&isoform_name=Isoform_1) |  | [In Vitro](http://www.ncbi.nlm.nih.gov/entrez/query.fcgi?cmd=Retrieve&db=PubMed&list_uids=,8051108,12551973,&dopt=Abstract) |  | Direct | | [Fanconi anemia group G protein](http://www.hprd.org/interactions?hprd_id=04262&isoform_id=04262_1&isoform_name=Isoform_1) |  | [In Vitro](http://www.ncbi.nlm.nih.gov/entrez/query.fcgi?cmd=Retrieve&db=PubMed&list_uids=,15367677,&dopt=Abstract) |  | Direct | | [Flap endonuclease 1](http://www.hprd.org/interactions?hprd_id=02670&isoform_id=02670_1&isoform_name=Isoform_1) |  | [In Vitro](http://www.ncbi.nlm.nih.gov/entrez/query.fcgi?cmd=Retrieve&db=PubMed&list_uids=,12853968,&dopt=Abstract) |  | Direct | | [Forkhead box protein M1](http://www.hprd.org/interactions?hprd_id=03823&isoform_id=03823_1&isoform_name=Isoform_1) |  | [In Vitro](http://www.ncbi.nlm.nih.gov/entrez/query.fcgi?cmd=Retrieve&db=PubMed&list_uids=,15024056,&dopt=Abstract) |  | Direct | | [Glial fibrillary acidic protein](http://www.hprd.org/interactions?hprd_id=00675&isoform_id=00675_1&isoform_name=Isoform_1) |  | [In Vivo ; In Vitro](http://www.ncbi.nlm.nih.gov/entrez/query.fcgi?cmd=Retrieve&db=PubMed&list_uids=,7822264,12177195,&dopt=Abstract) |  | Direct | | [Golgin 95](http://www.hprd.org/interactions?hprd_id=03989&isoform_id=03989_1&isoform_name=Isoform_1) |  | [In Vivo ; In Vitro](http://www.ncbi.nlm.nih.gov/entrez/query.fcgi?cmd=Retrieve&db=PubMed&list_uids=,10769027,9753325,&dopt=Abstract) |  | Direct | | [High mobility group AT hook 1](http://www.hprd.org/interactions?hprd_id=02829&isoform_id=02829_1&isoform_name=Isoform_1) |  | [In Vivo ; In Vitro](http://www.ncbi.nlm.nih.gov/entrez/query.fcgi?cmd=Retrieve&db=PubMed&list_uids=,1939057,&dopt=Abstract) |  | Direct | | [High mobility group AT hook 2](http://www.hprd.org/interactions?hprd_id=02827&isoform_id=02827_1&isoform_name=Isoform_1) |  | [In Vitro](http://www.ncbi.nlm.nih.gov/entrez/query.fcgi?cmd=Retrieve&db=PubMed&list_uids=,10636877,&dopt=Abstract) |  | Direct | | [Interleukin 16](http://www.hprd.org/interactions?hprd_id=04329&isoform_id=04329_1&isoform_name=Isoform_1) |  | [In Vivo ; In Vitro](http://www.ncbi.nlm.nih.gov/entrez/query.fcgi?cmd=Retrieve&db=PubMed&list_uids=,12450396,&dopt=Abstract) |  | Direct | | [Keratin 18](http://www.hprd.org/interactions?hprd_id=01020&isoform_id=01020_1&isoform_name=Isoform_1) |  | [In Vivo](http://www.ncbi.nlm.nih.gov/entrez/query.fcgi?cmd=Retrieve&db=PubMed&list_uids=,9524113,15368451,&dopt=Abstract) |  | Direct | | [Kinesin like protein 1](http://www.hprd.org/interactions?hprd_id=01023&isoform_id=01023_1&isoform_name=Isoform_1) |  | [In Vivo ; In Vitro ; Yeast 2 Hybrid](http://www.ncbi.nlm.nih.gov/entrez/query.fcgi?cmd=Retrieve&db=PubMed&list_uids=,9235942,8548803,&dopt=Abstract) |  | Direct | | [Lamin A/C](http://www.hprd.org/interactions?hprd_id=01035&isoform_id=01035_1&isoform_name=Isoform_1) |  | [In Vivo ; In Vitro](http://www.ncbi.nlm.nih.gov/entrez/query.fcgi?cmd=Retrieve&db=PubMed&list_uids=,7925482,2344612,&dopt=Abstract) |  | Direct | | [Lamin B1](http://www.hprd.org/interactions?hprd_id=01036&isoform_id=01036_1&isoform_name=Isoform_1) |  | [In Vivo ; In Vitro](http://www.ncbi.nlm.nih.gov/entrez/query.fcgi?cmd=Retrieve&db=PubMed&list_uids=,11901153,8034666,&dopt=Abstract) |  | Direct | | [LATS1](http://www.hprd.org/interactions?hprd_id=09147&isoform_id=09147_1&isoform_name=Isoform_1) |  | [In Vivo ; Yeast 2 Hybrid ; In Vitro](http://www.ncbi.nlm.nih.gov/entrez/query.fcgi?cmd=Retrieve&db=PubMed&list_uids=,9988268,12372621,&dopt=Abstract) |  | Direct | | [M phase phosphoprotein 1](http://www.hprd.org/interactions?hprd_id=09268&isoform_id=09268_1&isoform_name=Isoform_1) |  | [In Vitro ; In Vivo](http://www.ncbi.nlm.nih.gov/entrez/query.fcgi?cmd=Retrieve&db=PubMed&list_uids=,11470801,&dopt=Abstract) |  | Direct | | [MDM4](http://www.hprd.org/interactions?hprd_id=04082&isoform_id=04082_1&isoform_name=Isoform_1) |  | [In Vitro](http://www.ncbi.nlm.nih.gov/entrez/query.fcgi?cmd=Retrieve&db=PubMed&list_uids=,15735705,&dopt=Abstract) |  | Direct | | [Microtubule associated protein 4](http://www.hprd.org/interactions?hprd_id=01141&isoform_id=01141_1&isoform_name=Isoform_1) |  | [In Vivo ; In Vitro](http://www.ncbi.nlm.nih.gov/entrez/query.fcgi?cmd=Retrieve&db=PubMed&list_uids=,9398320,11683421,&dopt=Abstract) |  | Direct | | [Microtubule associated protein tau](http://www.hprd.org/interactions?hprd_id=01142&isoform_id=01142_1&isoform_name=Isoform_1) |  | [In Vitro](http://www.ncbi.nlm.nih.gov/entrez/query.fcgi?cmd=Retrieve&db=PubMed&list_uids=,9614189,&dopt=Abstract) |  | Direct | | [Myocyte specific enhancer factor 2C](http://www.hprd.org/interactions?hprd_id=02809&isoform_id=02809_1&isoform_name=Isoform_1) |  | [In Vitro](http://www.ncbi.nlm.nih.gov/entrez/query.fcgi?cmd=Retrieve&db=PubMed&list_uids=,16478538,&dopt=Abstract) |  | Direct | | [Nestin](http://www.hprd.org/interactions?hprd_id=09020&isoform_id=09020_1&isoform_name=Isoform_1) |  | [In Vivo ; In Vitro](http://www.ncbi.nlm.nih.gov/entrez/query.fcgi?cmd=Retrieve&db=PubMed&list_uids=,12832492,11278541,&dopt=Abstract) |  | Direct | | [NIR2](http://www.hprd.org/interactions?hprd_id=07497&isoform_id=07497_1&isoform_name=Isoform_1) |  | [In Vivo ; In Vitro](http://www.ncbi.nlm.nih.gov/entrez/query.fcgi?cmd=Retrieve&db=PubMed&list_uids=,15125835,&dopt=Abstract) |  | Direct | | [NSFL1C](http://www.hprd.org/interactions?hprd_id=09425&isoform_id=09425_1&isoform_name=Isoform_1) |  | [In Vivo ; In Vitro](http://www.ncbi.nlm.nih.gov/entrez/query.fcgi?cmd=Retrieve&db=PubMed&list_uids=,12810701,&dopt=Abstract) |  | Direct | | [Nucleophosmin 1](http://www.hprd.org/interactions?hprd_id=01246&isoform_id=01246_1&isoform_name=Isoform_1) |  | [In Vivo ; In Vitro](http://www.ncbi.nlm.nih.gov/entrez/query.fcgi?cmd=Retrieve&db=PubMed&list_uids=,12058066,11278991,&dopt=Abstract) |  | Direct | | [NUDE1](http://www.hprd.org/interactions?hprd_id=14815&isoform_id=14815_1&isoform_name=Isoform_1) |  | [In Vitro](http://www.ncbi.nlm.nih.gov/entrez/query.fcgi?cmd=Retrieve&db=PubMed&list_uids=,12556484,&dopt=Abstract) |  | Direct | | [NUP210](http://www.hprd.org/interactions?hprd_id=06368&isoform_id=06368_1&isoform_name=Isoform_1) |  | [In Vitro](http://www.ncbi.nlm.nih.gov/entrez/query.fcgi?cmd=Retrieve&db=PubMed&list_uids=,8672508,&dopt=Abstract) |  | Direct | | [p73](http://www.hprd.org/interactions?hprd_id=03587&isoform_id=03587_1&isoform_name=Isoform_1) |  | [In Vivo ; In Vitro](http://www.ncbi.nlm.nih.gov/entrez/query.fcgi?cmd=Retrieve&db=PubMed&list_uids=,12676926,&dopt=Abstract) |  | Direct | | [Patched protein homolog 1](http://www.hprd.org/interactions?hprd_id=03200&isoform_id=03200_1&isoform_name=Isoform_1) |  | [In Vivo ; In Vitro](http://www.ncbi.nlm.nih.gov/entrez/query.fcgi?cmd=Retrieve&db=PubMed&list_uids=,11331587,&dopt=Abstract) |  | Direct | | [PDZ binding kinase](http://www.hprd.org/interactions?hprd_id=17822&isoform_id=17822_1&isoform_name=Isoform_1) |  | [In Vitro](http://www.ncbi.nlm.nih.gov/entrez/query.fcgi?cmd=Retrieve&db=PubMed&list_uids=,15541388,&dopt=Abstract) |  | Direct | | [Pituitary tumor-transforming protein 1](http://www.hprd.org/interactions?hprd_id=04998&isoform_id=04998_1&isoform_name=Isoform_1) |  | [In Vivo ; In Vitro](http://www.ncbi.nlm.nih.gov/entrez/query.fcgi?cmd=Retrieve&db=PubMed&list_uids=,10656688,&dopt=Abstract) |  | Direct | | [Plectin 1](http://www.hprd.org/interactions?hprd_id=03180&isoform_id=03180_1&isoform_name=Isoform_1) |  | [In Vivo ; In Vitro](http://www.ncbi.nlm.nih.gov/entrez/query.fcgi?cmd=Retrieve&db=PubMed&list_uids=,8626512,&dopt=Abstract) |  | Direct | | [Protein tyrosine phosphatase, non receptor type 2](http://www.hprd.org/interactions?hprd_id=06768&isoform_id=06768_1&isoform_name=Isoform_1) |  | [In Vivo ; In Vitro](http://www.ncbi.nlm.nih.gov/entrez/query.fcgi?cmd=Retrieve&db=PubMed&list_uids=,15030318,&dopt=Abstract) |  | Direct | | [Protein tyrosine phosphatase, non-receptor type 1](http://www.hprd.org/interactions?hprd_id=01477&isoform_id=01477_1&isoform_name=Isoform_1) |  | [In Vitro](http://www.ncbi.nlm.nih.gov/entrez/query.fcgi?cmd=Retrieve&db=PubMed&list_uids=,8491187,9600099,&dopt=Abstract) |  | Direct | | [RAP1 GTPase activating protein 1](http://www.hprd.org/interactions?hprd_id=02609&isoform_id=02609_1&isoform_name=Isoform_1) |  | [In Vitro](http://www.ncbi.nlm.nih.gov/entrez/query.fcgi?cmd=Retrieve&db=PubMed&list_uids=,1406653,&dopt=Abstract) |  | Direct | | [Ras associated protein Rab4](http://www.hprd.org/interactions?hprd_id=01541&isoform_id=01541_1&isoform_name=Isoform_1) |  | [In Vitro](http://www.ncbi.nlm.nih.gov/entrez/query.fcgi?cmd=Retrieve&db=PubMed&list_uids=,1425574,&dopt=Abstract) |  | Direct | | [Ras associated protein Rab5B](http://www.hprd.org/interactions?hprd_id=01544&isoform_id=01544_1&isoform_name=Isoform_1) |  | [In Vitro](http://www.ncbi.nlm.nih.gov/entrez/query.fcgi?cmd=Retrieve&db=PubMed&list_uids=,10403367,&dopt=Abstract) |  | Direct | | [Replication protein A2, 32 kDa](http://www.hprd.org/interactions?hprd_id=01566&isoform_id=01566_1&isoform_name=Isoform_1) |  | [In Vivo ; In Vitro](http://www.ncbi.nlm.nih.gov/entrez/query.fcgi?cmd=Retrieve&db=PubMed&list_uids=,1318195,9295339,&dopt=Abstract) |  | Direct | | [Response gene to complement 32](http://www.hprd.org/interactions?hprd_id=17970&isoform_id=17970_1&isoform_name=Isoform_1) |  | [In Vivo ; In Vitro](http://www.ncbi.nlm.nih.gov/entrez/query.fcgi?cmd=Retrieve&db=PubMed&list_uids=,11687586,&dopt=Abstract) |  | Direct | | [Retinoblastoma 1](http://www.hprd.org/interactions?hprd_id=01574&isoform_id=01574_1&isoform_name=Isoform_1) |  | [In Vivo](http://www.ncbi.nlm.nih.gov/entrez/query.fcgi?cmd=Retrieve&db=PubMed&list_uids=,1756735,&dopt=Abstract) |  | Direct | | [Ribonucleotide reductase M2 subunit](http://www.hprd.org/interactions?hprd_id=01587&isoform_id=01587_1&isoform_name=Isoform_1) |  | [In Vitro](http://www.ncbi.nlm.nih.gov/entrez/query.fcgi?cmd=Retrieve&db=PubMed&list_uids=,9990288,&dopt=Abstract) |  | Direct | | [Ribosomal protein S6 kinase, 70kDa, polypeptide 1](http://www.hprd.org/interactions?hprd_id=10202&isoform_id=10202_1&isoform_name=Isoform_1) |  | [In Vivo ; In Vitro](http://www.ncbi.nlm.nih.gov/entrez/query.fcgi?cmd=Retrieve&db=PubMed&list_uids=,12586835,9271440,&dopt=Abstract) |  | Direct | | [Runt related transcription factor 2](http://www.hprd.org/interactions?hprd_id=02566&isoform_id=02566_1&isoform_name=Isoform_1) |  | [In Vitro](http://www.ncbi.nlm.nih.gov/entrez/query.fcgi?cmd=Retrieve&db=PubMed&list_uids=,16407259,&dopt=Abstract) |  | Direct | | [Sam68](http://www.hprd.org/interactions?hprd_id=03926&isoform_id=03926_1&isoform_name=Isoform_1) |  | [In Vivo ; In Vitro](http://www.ncbi.nlm.nih.gov/entrez/query.fcgi?cmd=Retrieve&db=PubMed&list_uids=,9315091,&dopt=Abstract) |  | Direct | | [Stathmin 1](http://www.hprd.org/interactions?hprd_id=01047&isoform_id=01047_1&isoform_name=Isoform_1) |  | [In Vivo](http://www.ncbi.nlm.nih.gov/entrez/query.fcgi?cmd=Retrieve&db=PubMed&list_uids=,8376365,11135364,8325880,&dopt=Abstract) |  | Direct | | [Stathmin like 2](http://www.hprd.org/interactions?hprd_id=09003&isoform_id=09003_1&isoform_name=Isoform_1) |  | [In Vitro](http://www.ncbi.nlm.nih.gov/entrez/query.fcgi?cmd=Retrieve&db=PubMed&list_uids=,9525956,9126608,&dopt=Abstract) |  | Direct | | [Survivin](http://www.hprd.org/interactions?hprd_id=04520&isoform_id=04520_1&isoform_name=Isoform_1) |  | [In Vivo ; In Vitro](http://www.ncbi.nlm.nih.gov/entrez/query.fcgi?cmd=Retrieve&db=PubMed&list_uids=,11069302,11861764,&dopt=Abstract) |  | Direct | | [Thymidine kinase soluble](http://www.hprd.org/interactions?hprd_id=01771&isoform_id=01771_1&isoform_name=Isoform_1) |  | [In Vitro](http://www.ncbi.nlm.nih.gov/entrez/query.fcgi?cmd=Retrieve&db=PubMed&list_uids=,9575153,14697231,&dopt=Abstract) |  | Direct | | [Transforming growth factor, beta receptor II (70/80kDa)](http://www.hprd.org/interactions?hprd_id=01823&isoform_id=01823_1&isoform_name=Isoform_1) |  | [In Vivo ; In Vitro](http://www.ncbi.nlm.nih.gov/entrez/query.fcgi?cmd=Retrieve&db=PubMed&list_uids=,9926943,&dopt=Abstract) |  | Direct | | [Tuberous sclerosis 1](http://www.hprd.org/interactions?hprd_id=05594&isoform_id=05594_1&isoform_name=Isoform_1) |  | [In Vivo ; In Vitro](http://www.ncbi.nlm.nih.gov/entrez/query.fcgi?cmd=Retrieve&db=PubMed&list_uids=,11444800,14551205,&dopt=Abstract) |  | Direct | | [Type 1 inositol 1,4,5 trisphosphate receptor](http://www.hprd.org/interactions?hprd_id=00925&isoform_id=00925_1&isoform_name=Isoform_1) |  | [In Vivo ; In Vitro](http://www.ncbi.nlm.nih.gov/entrez/query.fcgi?cmd=Retrieve&db=PubMed&list_uids=,14635192,16237118,&dopt=Abstract) |  | Direct | | [Ubiquitin activating enzyme 1](http://www.hprd.org/interactions?hprd_id=02440&isoform_id=02440_1&isoform_name=Isoform_1) |  | [In Vivo ; In Vitro](http://www.ncbi.nlm.nih.gov/entrez/query.fcgi?cmd=Retrieve&db=PubMed&list_uids=,7724583,7673335,&dopt=Abstract) |  | Direct | | [Ubiquitin conjugating enzyme E2A](http://www.hprd.org/interactions?hprd_id=02422&isoform_id=02422_1&isoform_name=Isoform_1) |  | [In Vivo](http://www.ncbi.nlm.nih.gov/entrez/query.fcgi?cmd=Retrieve&db=PubMed&list_uids=,11953320,&dopt=Abstract) |  | Direct | | [Vimentin](http://www.hprd.org/interactions?hprd_id=01899&isoform_id=01899_1&isoform_name=Isoform_1) |  | [In Vitro](http://www.ncbi.nlm.nih.gov/entrez/query.fcgi?cmd=Retrieve&db=PubMed&list_uids=,7983050,15345747,&dopt=Abstract) |  | Direct | | [CDC 25B](http://www.hprd.org/interactions?hprd_id=00307&isoform_id=00307_1&isoform_name=Isoform_1) |  | [In Vitro](http://www.ncbi.nlm.nih.gov/entrez/query.fcgi?cmd=Retrieve&db=PubMed&list_uids=,9585407,9733650,9268380,9141461,8440392,12107172,&dopt=Abstract) |  | Direct | | [Cyclin dependent kinase inhibitor 3](http://www.hprd.org/interactions?hprd_id=00450&isoform_id=00450_1&isoform_name=Isoform_1) |  | [In Vitro ; In Vivo ; Yeast 2 Hybrid](http://www.ncbi.nlm.nih.gov/entrez/query.fcgi?cmd=Retrieve&db=PubMed&list_uids=,8127873,&dopt=Abstract) |  | Direct | | [Cyclin A2](http://www.hprd.org/interactions?hprd_id=00453&isoform_id=00453_1&isoform_name=Isoform_1) |  | [In Vivo](http://www.ncbi.nlm.nih.gov/entrez/query.fcgi?cmd=Retrieve&db=PubMed&list_uids=,10924145,&dopt=Abstract) |  | Direct | | [Cyclin B1](http://www.hprd.org/interactions?hprd_id=00454&isoform_id=00454_1&isoform_name=Isoform_1) |  | [In Vivo ; In Vitro](http://www.ncbi.nlm.nih.gov/entrez/query.fcgi?cmd=Retrieve&db=PubMed&list_uids=,2570636,10362260,&dopt=Abstract) |  | Direct | | [Cyclin E1](http://www.hprd.org/interactions?hprd_id=00455&isoform_id=00455_1&isoform_name=Isoform_1) |  | [In Vitro](http://www.ncbi.nlm.nih.gov/entrez/query.fcgi?cmd=Retrieve&db=PubMed&list_uids=,1388288,&dopt=Abstract) |  | Direct | | [DNA damage inducible transcript 1](http://www.hprd.org/interactions?hprd_id=00528&isoform_id=00528_1&isoform_name=Isoform_1) |  | [In Vivo](http://www.ncbi.nlm.nih.gov/entrez/query.fcgi?cmd=Retrieve&db=PubMed&list_uids=,10973963,10362260,&dopt=Abstract) |  | Direct | | [ERCC2](http://www.hprd.org/interactions?hprd_id=00530&isoform_id=00530_1&isoform_name=Isoform_1) |  | [In Vivo](http://www.ncbi.nlm.nih.gov/entrez/query.fcgi?cmd=Retrieve&db=PubMed&list_uids=,8652557,&dopt=Abstract) |  | Direct | | [Fyn](http://www.hprd.org/interactions?hprd_id=00655&isoform_id=00655_1&isoform_name=Isoform_1) |  | [In Vivo](http://www.ncbi.nlm.nih.gov/entrez/query.fcgi?cmd=Retrieve&db=PubMed&list_uids=,8910336,&dopt=Abstract) |  | Direct | | [CDC25C](http://www.hprd.org/interactions?hprd_id=01146&isoform_id=01146_1&isoform_name=Isoform_1) |  | [In Vivo ; In Vitro](http://www.ncbi.nlm.nih.gov/entrez/query.fcgi?cmd=Retrieve&db=PubMed&list_uids=,11836499,9585407,9733650,9268380,9141461,8440392,8119945,17349584,&dopt=Abstract) |  | Direct | | [Lyn](http://www.hprd.org/interactions?hprd_id=01301&isoform_id=01301_1&isoform_name=Isoform_1) |  | [In Vivo ; In Vitro](http://www.ncbi.nlm.nih.gov/entrez/query.fcgi?cmd=Retrieve&db=PubMed&list_uids=,8910336,8051175,8084605,10564259,&dopt=Abstract) |  | Direct | | [Proliferating cell nuclear antigen](http://www.hprd.org/interactions?hprd_id=01456&isoform_id=01456_1&isoform_name=Isoform_1) |  | [In Vitro](http://www.ncbi.nlm.nih.gov/entrez/query.fcgi?cmd=Retrieve&db=PubMed&list_uids=,7949095,&dopt=Abstract) |  | Direct | | [Prothymosin alpha](http://www.hprd.org/interactions?hprd_id=01778&isoform_id=01778_1&isoform_name=Isoform_1) |  | [In Vitro](http://www.ncbi.nlm.nih.gov/entrez/query.fcgi?cmd=Retrieve&db=PubMed&list_uids=,11310559,&dopt=Abstract) |  | Direct | | [p53](http://www.hprd.org/interactions?hprd_id=01859&isoform_id=01859_1&isoform_name=Isoform_1) |  | [In Vivo ; In Vitro](http://www.ncbi.nlm.nih.gov/entrez/query.fcgi?cmd=Retrieve&db=PubMed&list_uids=,11327730,&dopt=Abstract) |  | Direct | | [WEE 1 tyrosine kinase](http://www.hprd.org/interactions?hprd_id=01907&isoform_id=01907_1&isoform_name=Isoform_1) |  | [In Vivo ; In Vitro](http://www.ncbi.nlm.nih.gov/entrez/query.fcgi?cmd=Retrieve&db=PubMed&list_uids=,10564259,9268380,12186947,8428596,&dopt=Abstract) |  | Direct | | [Fanconi anemia, complementation group C](http://www.hprd.org/interactions?hprd_id=01967&isoform_id=01967_1&isoform_name=Isoform_1) |  | [In Vivo](http://www.ncbi.nlm.nih.gov/entrez/query.fcgi?cmd=Retrieve&db=PubMed&list_uids=,9242535,&dopt=Abstract) |  | Direct | | [DNA Polymerase, alpha](http://www.hprd.org/interactions?hprd_id=02416&isoform_id=02416_1&isoform_name=Isoform_1) |  | [In Vitro ; In Vivo](http://www.ncbi.nlm.nih.gov/entrez/query.fcgi?cmd=Retrieve&db=PubMed&list_uids=,11259605,&dopt=Abstract) |  | Direct | | [TLE1](http://www.hprd.org/interactions?hprd_id=02557&isoform_id=02557_1&isoform_name=Isoform_1) |  | [In Vitro](http://www.ncbi.nlm.nih.gov/entrez/query.fcgi?cmd=Retrieve&db=PubMed&list_uids=,12397081,&dopt=Abstract) |  | Direct | | [Myelin transcription factor 1](http://www.hprd.org/interactions?hprd_id=02659&isoform_id=02659_1&isoform_name=Isoform_1) |  | [In Vivo ; In Vitro](http://www.ncbi.nlm.nih.gov/entrez/query.fcgi?cmd=Retrieve&db=PubMed&list_uids=,9001210,9268380,&dopt=Abstract) |  | Direct | | [Pin1](http://www.hprd.org/interactions?hprd_id=03031&isoform_id=03031_1&isoform_name=Isoform_1) |  | [In Vivo](http://www.ncbi.nlm.nih.gov/entrez/query.fcgi?cmd=Retrieve&db=PubMed&list_uids=,11774038,&dopt=Abstract) |  | Direct | | [DAB2](http://www.hprd.org/interactions?hprd_id=03139&isoform_id=03139_1&isoform_name=Isoform_1) |  | [In Vitro](http://www.ncbi.nlm.nih.gov/entrez/query.fcgi?cmd=Retrieve&db=PubMed&list_uids=,12881709,&dopt=Abstract) |  | Direct | | [Chromatin assembly factor 1 subunit B](http://www.hprd.org/interactions?hprd_id=03147&isoform_id=03147_1&isoform_name=Isoform_1) |  | [In Vitro](http://www.ncbi.nlm.nih.gov/entrez/query.fcgi?cmd=Retrieve&db=PubMed&list_uids=,10938080,&dopt=Abstract) |  | Direct | | [14-3-3 sigma](http://www.hprd.org/interactions?hprd_id=03185&isoform_id=03185_1&isoform_name=Isoform_1) |  | [In Vivo](http://www.ncbi.nlm.nih.gov/entrez/query.fcgi?cmd=Retrieve&db=PubMed&list_uids=,10524633,&dopt=Abstract) |  | Direct | | [Dynamin 2](http://www.hprd.org/interactions?hprd_id=03852&isoform_id=03852_1&isoform_name=Isoform_1) |  | [In Vivo ; In Vitro](http://www.ncbi.nlm.nih.gov/entrez/query.fcgi?cmd=Retrieve&db=PubMed&list_uids=,7590285,&dopt=Abstract) |  | Direct | | [Protein kinase Myt1](http://www.hprd.org/interactions?hprd_id=03920&isoform_id=03920_1&isoform_name=Isoform_1) |  | [In Vitro ; In Vivo](http://www.ncbi.nlm.nih.gov/entrez/query.fcgi?cmd=Retrieve&db=PubMed&list_uids=,10373560,10504341,9001210,9268380,12912980,&dopt=Abstract) |  | Direct | | [Cyclin B2](http://www.hprd.org/interactions?hprd_id=04131&isoform_id=04131_1&isoform_name=Isoform_1) |  | [In Vivo ; In Vitro](http://www.ncbi.nlm.nih.gov/entrez/query.fcgi?cmd=Retrieve&db=PubMed&list_uids=,9926943,&dopt=Abstract) |  | Direct | | [Cyclin A1](http://www.hprd.org/interactions?hprd_id=04946&isoform_id=04946_1&isoform_name=Isoform_1) |  | [In Vivo](http://www.ncbi.nlm.nih.gov/entrez/query.fcgi?cmd=Retrieve&db=PubMed&list_uids=,8565853,&dopt=Abstract) |  | Direct | | [NOT7](http://www.hprd.org/interactions?hprd_id=05370&isoform_id=05370_1&isoform_name=Isoform_1) |  | [In Vivo](http://www.ncbi.nlm.nih.gov/entrez/query.fcgi?cmd=Retrieve&db=PubMed&list_uids=,10602502,&dopt=Abstract) |  | Direct | | [GADD45 beta](http://www.hprd.org/interactions?hprd_id=05382&isoform_id=05382_1&isoform_name=Isoform_1) |  | [In Vivo](http://www.ncbi.nlm.nih.gov/entrez/query.fcgi?cmd=Retrieve&db=PubMed&list_uids=,10973963,&dopt=Abstract) |  | Direct | | [GADD45 gamma](http://www.hprd.org/interactions?hprd_id=05383&isoform_id=05383_1&isoform_name=Isoform_1) |  | [In Vivo](http://www.ncbi.nlm.nih.gov/entrez/query.fcgi?cmd=Retrieve&db=PubMed&list_uids=,10973963,&dopt=Abstract) |  | Direct | | [Leucine zipper, putative tumor suppressor 1](http://www.hprd.org/interactions?hprd_id=05945&isoform_id=05945_1&isoform_name=Isoform_1) |  | [In Vivo](http://www.ncbi.nlm.nih.gov/entrez/query.fcgi?cmd=Retrieve&db=PubMed&list_uids=,11504921,&dopt=Abstract) |  | Direct | | [Golgi reassembly stacking protein 1](http://www.hprd.org/interactions?hprd_id=06038&isoform_id=06038_1&isoform_name=Isoform_1) |  | [In Vitro](http://www.ncbi.nlm.nih.gov/entrez/query.fcgi?cmd=Retrieve&db=PubMed&list_uids=,12839990,&dopt=Abstract) |  | Direct | | [Ubiquitin specifc protease 16](http://www.hprd.org/interactions?hprd_id=06881&isoform_id=06881_1&isoform_name=Isoform_1) |  | [In Vivo ; In Vitro](http://www.ncbi.nlm.nih.gov/entrez/query.fcgi?cmd=Retrieve&db=PubMed&list_uids=,10077596,&dopt=Abstract) |  | Direct | | [DUP](http://www.hprd.org/interactions?hprd_id=06900&isoform_id=06900_1&isoform_name=Isoform_1) |  | [In Vivo ; In Vitro](http://www.ncbi.nlm.nih.gov/entrez/query.fcgi?cmd=Retrieve&db=PubMed&list_uids=,14993212,&dopt=Abstract) |  | Direct | | [Heat shock 70kDa protein 2](http://www.hprd.org/interactions?hprd_id=07174&isoform_id=07174_1&isoform_name=Isoform_1) |  | [In Vitro](http://www.ncbi.nlm.nih.gov/entrez/query.fcgi?cmd=Retrieve&db=PubMed&list_uids=,9247342,&dopt=Abstract) |  | Direct | | [Sperm associated antigen 5](http://www.hprd.org/interactions?hprd_id=10246&isoform_id=10246_1&isoform_name=Isoform_1) |  | [In Vitro](http://www.ncbi.nlm.nih.gov/entrez/query.fcgi?cmd=Retrieve&db=PubMed&list_uids=,11549262,&dopt=Abstract) |  | Direct | | [Zinc finger protein 145](http://www.hprd.org/interactions?hprd_id=11762&isoform_id=11762_1&isoform_name=Isoform_1) |  | [In Vivo ; In Vitro](http://www.ncbi.nlm.nih.gov/entrez/query.fcgi?cmd=Retrieve&db=PubMed&list_uids=,10497277,&dopt=Abstract) |  | Direct | | [Cyclin dependent kinase 7](http://www.hprd.org/interactions?hprd_id=15993&isoform_id=15993_1&isoform_name=Isoform_1) |  | [In Vitro](http://www.ncbi.nlm.nih.gov/entrez/query.fcgi?cmd=Retrieve&db=PubMed&list_uids=,7944411,11113184,&dopt=Abstract) |  | Direct | | [C10orf3 protein](http://www.hprd.org/interactions?hprd_id=12569&isoform_id=12569_1&isoform_name=Isoform_1) |  | [In Vitro](http://www.ncbi.nlm.nih.gov/entrez/query.fcgi?cmd=Retrieve&db=PubMed&list_uids=,16198290,&dopt=Abstract) |  | Direct | | [c-Myc](http://www.hprd.org/interactions?hprd_id=01818&isoform_id=01818_1&isoform_name=Isoform_1) |  | [In Vivo](http://www.ncbi.nlm.nih.gov/entrez/query.fcgi?cmd=Retrieve&db=PubMed&list_uids=,1748630,&dopt=Abstract) |  | Direct | | [HIV-1 Tat interacting protein, 60kDa](http://www.hprd.org/interactions?hprd_id=03245&isoform_id=03245_1&isoform_name=Isoform_1) |  | [In Vivo ; In Vitro](http://www.ncbi.nlm.nih.gov/entrez/query.fcgi?cmd=Retrieve&db=PubMed&list_uids=,12468530,&dopt=Abstract) |  | Direct | | [Myeloid cell nuclear differentiation antigen](http://www.hprd.org/interactions?hprd_id=15932&isoform_id=15932_1&isoform_name=Isoform_1) |  | [In Vitro](http://www.ncbi.nlm.nih.gov/entrez/query.fcgi?cmd=Retrieve&db=PubMed&list_uids=,16458891,&dopt=Abstract) |  | Direct | | [BRCA2](http://www.hprd.org/interactions?hprd_id=02554&isoform_id=02554_1&isoform_name=Isoform_1) |  | [In Vitro](http://www.ncbi.nlm.nih.gov/entrez/query.fcgi?cmd=Retrieve&db=PubMed&list_uids=,15800651,&dopt=Abstract) |  | Direct | | [PP2A, subunit A, R1 beta](http://www.hprd.org/interactions?hprd_id=04384&isoform_id=04384_1&isoform_name=Isoform_1) |  | [In Vivo](http://www.ncbi.nlm.nih.gov/entrez/query.fcgi?cmd=Retrieve&db=PubMed&list_uids=,17540176,&dopt=Abstract) |  | Direct | | [Protein phosphatase 2, regulatory subunit A , alpha isoform](http://www.hprd.org/interactions?hprd_id=16184&isoform_id=16184_1&isoform_name=Isoform_1) |  | [In Vivo](http://www.ncbi.nlm.nih.gov/entrez/query.fcgi?cmd=Retrieve&db=PubMed&list_uids=,17540176,&dopt=Abstract) |  | Direct | | [Fanconi anemia, complementation group A](http://www.hprd.org/interactions?hprd_id=06186&isoform_id=06186_1&isoform_name=Isoform_1) |  | [In Vitro](http://www.ncbi.nlm.nih.gov/entrez/query.fcgi?cmd=Retrieve&db=PubMed&list_uids=,15367677,&dopt=Abstract) |  | Direct | | [CDC2](http://www.hprd.org/interactions?hprd_id=00302&isoform_id=00302_1&isoform_name=Isoform_1) |  | [In Vivo](http://www.ncbi.nlm.nih.gov/entrez/query.fcgi?cmd=Retrieve&db=PubMed&list_uids=17192257&dopt=Abstract) |  | Direct | | [Baculoviral IAP repeat containing protein 6](http://www.hprd.org/interactions?hprd_id=05731&isoform_id=05731_1&isoform_name=Isoform_1) |  | [In Vivo](http://www.ncbi.nlm.nih.gov/entrez/query.fcgi?cmd=Retrieve&db=PubMed&list_uids=,18329369,&dopt=Abstract) |  | Direct | | [Cyclin dependent kinase inhibitor 1A](http://www.hprd.org/interactions?hprd_id=00298&isoform_id=00298_1&isoform_name=Isoform_1) [Cyclin B1](http://www.hprd.org/interactions?hprd_id=00454&isoform_id=00454_1&isoform_name=Isoform_1) [Proliferating cell nuclear antigen](http://www.hprd.org/interactions?hprd_id=01456&isoform_id=01456_1&isoform_name=Isoform_1) |  | [In Vitro ; In Vivo](http://www.ncbi.nlm.nih.gov/entrez/query.fcgi?cmd=Retrieve&db=PubMed&list_uids=11559705&dopt=Abstract) |  | Complex | | [Cyclin B1](http://www.hprd.org/interactions?hprd_id=00454&isoform_id=00454_1&isoform_name=Isoform_1) [MYT1](http://www.hprd.org/interactions?hprd_id=03920&isoform_id=03920_1&isoform_name=Isoform_1) |  | [In Vitro](http://www.ncbi.nlm.nih.gov/entrez/query.fcgi?cmd=Retrieve&db=PubMed&list_uids=10373560&dopt=Abstract) |  | Complex | | [Cyclin B1](http://www.hprd.org/interactions?hprd_id=00454&isoform_id=00454_1&isoform_name=Isoform_1) [Golgi reassembly stacking protein 1](http://www.hprd.org/interactions?hprd_id=06038&isoform_id=06038_1&isoform_name=Isoform_1) |  | [In Vivo ; In Vitro](http://www.ncbi.nlm.nih.gov/entrez/query.fcgi?cmd=Retrieve&db=PubMed&list_uids=12839990&dopt=Abstract) |  | Complex | | [Cyclin B1](http://www.hprd.org/interactions?hprd_id=00454&isoform_id=00454_1&isoform_name=Isoform_1) [Peptidyl prolyl isomerase G](http://www.hprd.org/interactions?hprd_id=12083&isoform_id=12083_1&isoform_name=Isoform_1) |  | [In Vitro](http://www.ncbi.nlm.nih.gov/entrez/query.fcgi?cmd=Retrieve&db=PubMed&list_uids=15016823&dopt=Abstract) |  | Complex | | [TFIIH 62 kDa subunit](http://www.hprd.org/interactions?hprd_id=01807&isoform_id=01807_1&isoform_name=Isoform_1) [GTF2H4](http://www.hprd.org/interactions?hprd_id=03456&isoform_id=03456_1&isoform_name=Isoform_1) [MAT1](http://www.hprd.org/interactions?hprd_id=04042&isoform_id=04042_1&isoform_name=Isoform_1) [Cyclin H](http://www.hprd.org/interactions?hprd_id=09059&isoform_id=09059_1&isoform_name=Isoform_1) |  | [In Vivo ; In Vitro](http://www.ncbi.nlm.nih.gov/entrez/query.fcgi?cmd=Retrieve&db=PubMed&list_uids=9130708&dopt=Abstract) |  | Complex | | [TGF beta receptor, type II](http://www.hprd.org/interactions?hprd_id=01823&isoform_id=01823_1&isoform_name=Isoform_1) [Cyclin B2](http://www.hprd.org/interactions?hprd_id=04131&isoform_id=04131_1&isoform_name=Isoform_1) |  | [In Vivo](http://www.ncbi.nlm.nih.gov/entrez/query.fcgi?cmd=Retrieve&db=PubMed&list_uids=9926943&dopt=Abstract) |  | Complex | | [Cyclin F](http://www.hprd.org/interactions?hprd_id=02574&isoform_id=02574_1&isoform_name=Isoform_1) [Cyclin B1](http://www.hprd.org/interactions?hprd_id=00454&isoform_id=00454_1&isoform_name=Isoform_1) |  | [In Vivo](http://www.ncbi.nlm.nih.gov/entrez/query.fcgi?cmd=Retrieve&db=PubMed&list_uids=10716937&dopt=Abstract) |  | Complex | | [Cyclin B1](http://www.hprd.org/interactions?hprd_id=00454&isoform_id=00454_1&isoform_name=Isoform_1) [TLE1](http://www.hprd.org/interactions?hprd_id=02557&isoform_id=02557_1&isoform_name=Isoform_1) [Transducin like enhancer of split 3](http://www.hprd.org/interactions?hprd_id=02558&isoform_id=02558_1&isoform_name=Isoform_1) |  | [In Vitro](http://www.ncbi.nlm.nih.gov/entrez/query.fcgi?cmd=Retrieve&db=PubMed&list_uids=12397081&dopt=Abstract) |  | Complex | | [RalBP1 interacting protein 2](http://www.hprd.org/interactions?hprd_id=02262&isoform_id=02262_1&isoform_name=Isoform_1) [Cyclin B1](http://www.hprd.org/interactions?hprd_id=00454&isoform_id=00454_1&isoform_name=Isoform_1) [Adaptor related protein complex 2 alpha 1 subunit](http://www.hprd.org/interactions?hprd_id=03016&isoform_id=03016_1&isoform_name=Isoform_1) [Numb homolog](http://www.hprd.org/interactions?hprd_id=04767&isoform_id=04767_1&isoform_name=Isoform_1) [Epsin 1](http://www.hprd.org/interactions?hprd_id=06270&isoform_id=06270_1&isoform_name=Isoform_1) [RalA binding protein 1](http://www.hprd.org/interactions?hprd_id=09013&isoform_id=09013_1&isoform_name=Isoform_1) |  | [In Vivo ; In Vitro](http://www.ncbi.nlm.nih.gov/entrez/query.fcgi?cmd=Retrieve&db=PubMed&list_uids=12775724&dopt=Abstract) |  | Complex | | [Fanconi anemia group G protein](http://www.hprd.org/interactions?hprd_id=04262&isoform_id=04262_1&isoform_name=Isoform_1) [Tumor rejection antigen 1](http://www.hprd.org/interactions?hprd_id=01860&isoform_id=01860_1&isoform_name=Isoform_1) [Fanconi anemia, complementation group A](http://www.hprd.org/interactions?hprd_id=06186&isoform_id=06186_1&isoform_name=Isoform_1) [Fanconi anemia, complementation group C](http://www.hprd.org/interactions?hprd_id=01967&isoform_id=01967_1&isoform_name=Isoform_1) [Fanconi anemia protein E](http://www.hprd.org/interactions?hprd_id=02943&isoform_id=02943_1&isoform_name=Isoform_1) [Fanconi anemia, complementation group F](http://www.hprd.org/interactions?hprd_id=04589&isoform_id=04589_1&isoform_name=Isoform_1) |  | [In Vivo](http://www.ncbi.nlm.nih.gov/entrez/query.fcgi?cmd=Retrieve&db=PubMed&list_uids=15082718&dopt=Abstract) |  | Complex | | | | --- | --- | --- | --- | --- | --- | --- | --- | --- | --- | --- | --- | --- | --- | --- | --- | --- | --- | --- | --- | --- | --- | --- | --- | --- | --- | --- | --- | --- | --- | --- | --- | --- | --- | --- | --- | --- | --- | --- | --- | --- | --- | --- | --- | --- | --- | --- | --- | --- | --- | --- | --- | --- | --- | --- | --- | --- | --- | --- | --- | --- | --- | --- | --- | --- | --- | --- | --- | --- | --- | --- | --- | --- | --- | --- | --- | --- | --- | --- | --- | --- | --- | --- | --- | --- | --- | --- | --- | --- | --- | --- | --- | --- | --- | --- | --- | --- | --- | --- | --- | --- | --- | --- | --- | --- | --- | --- | --- | --- | --- | --- | --- | --- | --- | --- | --- | --- | --- | --- | --- | --- | --- | --- | --- | --- | --- | --- | --- | --- | --- | --- | --- | --- | --- | --- | --- | --- | --- | --- | --- | --- | --- | --- | --- | --- | --- | --- | --- | --- | --- | --- | --- | --- | --- | --- | --- | --- | --- | --- | --- | --- | --- | --- | --- | --- | --- | --- | --- | --- | --- | --- | --- | --- | --- | --- | --- | --- | --- | --- | --- | --- | --- | --- | --- | --- | --- | --- | --- | --- | --- | --- | --- | --- | --- | --- | --- | --- | --- | --- | --- | --- | --- | --- | --- | --- | --- | --- | --- | --- | --- | --- | --- | --- | --- | --- | --- | --- | --- | --- | --- | --- | --- | --- | --- | --- | --- | --- | --- | --- | --- | --- | --- | --- | --- | --- | --- | --- | --- | --- | --- | --- | --- | --- | --- | --- | --- | --- | --- | --- | --- | --- | --- | --- | --- | --- | --- | --- | --- | --- | --- | --- | --- | --- | --- | --- | --- | --- | --- | --- | --- | --- | --- | --- | --- | --- | --- | --- | --- | --- | --- | --- | --- | --- | --- | --- | --- | --- | --- | --- | --- | --- | --- | --- | --- | --- | --- | --- | --- | --- | --- | --- | --- | --- | --- | --- | --- | --- | --- | --- | --- | --- | --- | --- | --- | --- | --- | --- | --- | --- | --- | --- | --- | --- | --- | --- | --- | --- | --- | --- | --- | --- | --- | --- | --- | --- | --- | --- | --- | --- | --- | --- | --- | --- | --- | --- | --- | --- | --- | --- | --- | --- | --- | --- | --- | --- | --- | --- | --- | --- | --- | --- | --- | --- | --- | --- | --- | --- | --- | --- | --- | --- | --- | --- | --- | --- | --- | --- | --- | --- | --- | --- | --- | --- | --- | --- | --- | --- | --- | --- | --- | --- | --- | --- | --- | --- | --- | --- | --- | --- | --- | --- | --- | --- | --- | --- | --- | --- | --- | --- | --- | --- | --- | --- | --- | --- | --- | --- | --- | --- | --- | --- | --- | --- | --- | --- | --- | --- | --- | --- | --- | --- | --- | --- | --- | --- | --- | --- | --- | --- | --- | --- | --- | --- | --- | --- | --- | --- | --- | --- | --- | --- | --- | --- | --- | --- | --- | --- | --- | --- | --- | --- | --- | --- | --- | --- | --- | --- | --- | --- | --- | --- | --- | --- | --- | --- | --- | --- | --- | --- | --- | --- | --- | --- | --- | --- | --- | --- | --- | --- | --- | --- | --- | --- | --- | --- | --- | --- | --- | --- | --- | --- | --- | --- | --- | --- | --- | --- | --- | --- | --- | --- | --- | --- | --- | --- | --- | --- | --- | --- | --- | --- | --- | --- | --- | --- | --- | --- | --- | --- | --- | --- | --- | --- | --- | --- | --- | --- | --- | --- | --- | --- | --- | --- | --- | --- | --- | --- | --- | --- | --- | --- | --- | --- | --- | --- | --- | --- | --- | --- | --- | --- | --- | --- | --- | --- | --- | --- | --- | --- | --- | --- | --- | --- | --- | --- | --- | --- | --- | --- | --- | --- | --- | --- | --- | --- | --- | --- | --- | --- | --- | --- | --- | --- | --- | --- | --- | --- | --- | --- | --- | --- | --- | --- | --- | --- | --- | --- | --- | --- | --- | --- | --- | --- | --- | --- | --- | --- | --- | --- | --- | --- | --- | --- | --- | --- | --- | --- | --- | --- | --- | --- | --- | --- | --- | --- | --- | --- | --- | --- | --- | --- | --- | --- | --- | --- | --- | --- | --- | --- | --- | --- | --- | --- | --- | --- | --- | --- | --- | --- | --- | --- | --- | --- | --- | |
| --- | --- | --- | --- | --- | --- | --- | --- | --- | --- | --- | --- | --- | --- | --- | --- | --- | --- | --- | --- | --- | --- | --- | --- | --- | --- | --- | --- | --- | --- | --- | --- | --- | --- | --- | --- | --- | --- | --- | --- | --- | --- | --- | --- | --- | --- | --- | --- | --- | --- | --- | --- | --- | --- | --- | --- | --- | --- | --- | --- | --- | --- | --- | --- | --- | --- | --- | --- | --- | --- | --- | --- | --- | --- | --- | --- | --- | --- | --- | --- | --- | --- | --- | --- | --- | --- | --- | --- | --- | --- | --- | --- | --- | --- | --- | --- | --- | --- | --- | --- | --- | --- | --- | --- | --- | --- | --- | --- | --- | --- | --- | --- | --- | --- | --- | --- | --- | --- | --- | --- | --- | --- | --- | --- | --- | --- | --- | --- | --- | --- | --- | --- | --- | --- | --- | --- | --- | --- | --- | --- | --- | --- | --- | --- | --- | --- | --- | --- | --- | --- | --- | --- | --- | --- | --- | --- | --- | --- | --- | --- | --- | --- | --- | --- | --- | --- | --- | --- | --- | --- | --- | --- | --- | --- | --- | --- | --- | --- | --- | --- | --- | --- | --- | --- | --- | --- | --- | --- | --- | --- | --- | --- | --- | --- | --- | --- | --- | --- | --- | --- | --- | --- | --- | --- | --- | --- | --- | --- | --- | --- | --- | --- | --- | --- | --- | --- | --- | --- | --- | --- | --- | --- | --- | --- | --- | --- | --- | --- | --- | --- | --- | --- | --- | --- | --- | --- | --- | --- | --- | --- | --- | --- | --- | --- | --- | --- | --- | --- | --- | --- | --- | --- | --- | --- | --- | --- | --- | --- | --- | --- | --- | --- | --- | --- | --- | --- | --- | --- | --- | --- | --- | --- | --- | --- | --- | --- | --- | --- | --- | --- | --- | --- | --- | --- | --- | --- | --- | --- | --- | --- | --- | --- | --- | --- | --- | --- | --- | --- | --- | --- | --- | --- | --- | --- | --- | --- | --- | --- | --- | --- | --- | --- | --- | --- | --- | --- | --- | --- | --- | --- | --- | --- | --- | --- | --- | --- | --- | --- | --- | --- | --- | --- | --- | --- | --- | --- | --- | --- | --- | --- | --- | --- | --- | --- | --- | --- | --- | --- | --- | --- | --- | --- | --- | --- | --- | --- | --- | --- | --- | --- | --- | --- | --- | --- | --- | --- | --- | --- | --- | --- | --- | --- | --- | --- | --- | --- | --- | --- | --- | --- | --- | --- | --- | --- | --- | --- | --- | --- | --- | --- | --- | --- | --- | --- | --- | --- | --- | --- | --- | --- | --- | --- | --- | --- | --- | --- | --- | --- | --- | --- | --- | --- | --- | --- | --- | --- | --- | --- | --- | --- | --- | --- | --- | --- | --- | --- | --- | --- | --- | --- | --- | --- | --- | --- | --- | --- | --- | --- | --- | --- | --- | --- | --- | --- | --- | --- | --- | --- | --- | --- | --- | --- | --- | --- | --- | --- | --- | --- | --- | --- | --- | --- | --- | --- | --- | --- | --- | --- | --- | --- | --- | --- | --- | --- | --- | --- | --- | --- | --- | --- | --- | --- | --- | --- | --- | --- | --- | --- | --- | --- | --- | --- | --- | --- | --- | --- | --- | --- | --- | --- | --- | --- | --- | --- | --- | --- | --- | --- | --- | --- | --- | --- | --- | --- | --- | --- | --- | --- | --- | --- | --- | --- | --- | --- | --- | --- | --- | --- | --- | --- | --- | --- | --- | --- | --- | --- | --- | --- | --- | --- | --- | --- | --- | --- | --- | --- | --- | --- | --- | --- | --- | --- | --- | --- | --- | --- | --- | --- | --- | --- | --- | --- | --- | --- | --- | --- | --- | --- | --- | --- | --- | --- | --- | --- | --- | --- | --- | --- | --- | --- | --- | --- | --- | --- | --- | --- | --- | --- | --- | --- | --- | --- | --- | --- | --- | --- | --- | --- | --- | --- | --- | --- | --- | --- | --- | --- | --- | --- | --- | --- | --- | --- | --- | --- | --- | --- | --- | --- | --- | --- | --- | --- | --- | --- | --- | --- | --- | --- | --- | --- | --- | --- | --- | --- | --- | --- | --- | --- | --- | --- | --- | --- | --- | --- | --- | --- | --- | --- | --- | --- | --- | --- | --- | --- | --- | --- | --- | --- | --- | --- | --- | --- | --- | --- | --- | --- | --- | --- | --- | --- |

**PLK1**

|  | | 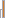 | **Protein Interactions** | 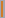 |  | | --- | --- | --- | --- |  |  | |  | | --- | | | **PROTEIN INTERACTORS** |  |  |  |  | | --- | --- | --- | --- | --- | | **Name of Interactor** |  | **Experiment Type** |  | **Type** | | [BRCA2](http://www.hprd.org/interactions?hprd_id=02554&isoform_id=02554_1&isoform_name=Isoform_1) |  | [In Vivo ; In Vitro](http://www.ncbi.nlm.nih.gov/entrez/query.fcgi?cmd=Retrieve&db=PubMed&list_uids=,12815053,&dopt=Abstract) |  | Direct | | [BUB1](http://www.hprd.org/interactions?hprd_id=03907&isoform_id=03907_1&isoform_name=Isoform_1) |  | [In Vivo ; In Vitro](http://www.ncbi.nlm.nih.gov/entrez/query.fcgi?cmd=Retrieve&db=PubMed&list_uids=,16760428,&dopt=Abstract) |  | Direct | | [CDC25C](http://www.hprd.org/interactions?hprd_id=01146&isoform_id=01146_1&isoform_name=Isoform_1) |  | [In Vivo ; In Vitro](http://www.ncbi.nlm.nih.gov/entrez/query.fcgi?cmd=Retrieve&db=PubMed&list_uids=,11202906,12595692,11897663,16753148,14532005 ,&dopt=Abstract) |  | Direct | | [CHK2 checkpoint homolog (S. pombe)](http://www.hprd.org/interactions?hprd_id=05084&isoform_id=05084_1&isoform_name=Isoform_1) |  | [In Vivo ; In Vitro](http://www.ncbi.nlm.nih.gov/entrez/query.fcgi?cmd=Retrieve&db=PubMed&list_uids=,12493754,11901158,12242661,10973490,12024051,&dopt=Abstract) |  | Direct | | [Chromosome 7 open reading frame 20](http://www.hprd.org/interactions?hprd_id=12909&isoform_id=12909_1&isoform_name=Isoform_1) |  | [Yeast 2 Hybrid](http://www.ncbi.nlm.nih.gov/entrez/query.fcgi?cmd=Retrieve&db=PubMed&list_uids=,16169070,&dopt=Abstract) |  | Direct | | [Cyclin B1](http://www.hprd.org/interactions?hprd_id=00454&isoform_id=00454_1&isoform_name=Isoform_1) |  | [In Vivo](http://www.ncbi.nlm.nih.gov/entrez/query.fcgi?cmd=Retrieve&db=PubMed&list_uids=,11242082,&dopt=Abstract) |  | Direct | | [Epithelial cell transforming sequence 2 oncogene](http://www.hprd.org/interactions?hprd_id=11860&isoform_id=11860_1&isoform_name=Isoform_1) |  | [In Vitro](http://www.ncbi.nlm.nih.gov/entrez/query.fcgi?cmd=Retrieve&db=PubMed&list_uids=,16247472,&dopt=Abstract) |  | Direct | | [FLJ20105 protein](http://www.hprd.org/interactions?hprd_id=10974&isoform_id=10974_1&isoform_name=Isoform_1) |  | [In Vivo](http://www.ncbi.nlm.nih.gov/entrez/query.fcgi?cmd=Retrieve&db=PubMed&list_uids=20360068&dopt=Abstract) |  | Direct | | [Hypothetical protein KIAA0980](http://www.hprd.org/interactions?hprd_id=11120&isoform_id=11120_1&isoform_name=Isoform_1) |  | [In Vitro](http://www.ncbi.nlm.nih.gov/entrez/query.fcgi?cmd=Retrieve&db=PubMed&list_uids=,12852856,&dopt=Abstract) |  | Direct | | [MCM7](http://www.hprd.org/interactions?hprd_id=01154&isoform_id=01154_1&isoform_name=Isoform_1) |  | [In Vivo ; In Vitro](http://www.ncbi.nlm.nih.gov/entrez/query.fcgi?cmd=Retrieve&db=PubMed&list_uids=,15654075,&dopt=Abstract) |  | Direct | | [MDG1](http://www.hprd.org/interactions?hprd_id=07047&isoform_id=07047_1&isoform_name=Isoform_1) |  | [Yeast 2 Hybrid](http://www.ncbi.nlm.nih.gov/entrez/query.fcgi?cmd=Retrieve&db=PubMed&list_uids=,16169070,&dopt=Abstract) |  | Direct | | [Melanoma antigen family D1](http://www.hprd.org/interactions?hprd_id=02202&isoform_id=02202_1&isoform_name=Isoform_1) |  | [Yeast 2 Hybrid](http://www.ncbi.nlm.nih.gov/entrez/query.fcgi?cmd=Retrieve&db=PubMed&list_uids=,16169070,&dopt=Abstract) |  | Direct | | [Minichromosome maintenance protein 2](http://www.hprd.org/interactions?hprd_id=00303&isoform_id=00303_1&isoform_name=Isoform_1) |  | [In Vivo](http://www.ncbi.nlm.nih.gov/entrez/query.fcgi?cmd=Retrieve&db=PubMed&list_uids=,15654075,&dopt=Abstract) |  | Direct | | [Myelin transcription factor 1](http://www.hprd.org/interactions?hprd_id=02659&isoform_id=02659_1&isoform_name=Isoform_1) |  | [In Vitro](http://www.ncbi.nlm.nih.gov/entrez/query.fcgi?cmd=Retrieve&db=PubMed&list_uids=,12738781,&dopt=Abstract) |  | Direct | | [Nucleophosmin 1](http://www.hprd.org/interactions?hprd_id=01246&isoform_id=01246_1&isoform_name=Isoform_1) |  | [In Vitro ; In Vivo](http://www.ncbi.nlm.nih.gov/entrez/query.fcgi?cmd=Retrieve&db=PubMed&list_uids=,15190079,&dopt=Abstract) |  | Direct | | [NUDC](http://www.hprd.org/interactions?hprd_id=10122&isoform_id=10122_1&isoform_name=Isoform_1) |  | [In Vivo ; In Vitro](http://www.ncbi.nlm.nih.gov/entrez/query.fcgi?cmd=Retrieve&db=PubMed&list_uids=,12852857,&dopt=Abstract) |  | Direct | | [Pin1](http://www.hprd.org/interactions?hprd_id=03031&isoform_id=03031_1&isoform_name=Isoform_1) |  | [In Vitro](http://www.ncbi.nlm.nih.gov/entrez/query.fcgi?cmd=Retrieve&db=PubMed&list_uids=,9499405,16118204,&dopt=Abstract) |  | Direct | | [Proteasome subunit alpha, type 5](http://www.hprd.org/interactions?hprd_id=01464&isoform_id=01464_1&isoform_name=Isoform_1) |  | [In Vivo](http://www.ncbi.nlm.nih.gov/entrez/query.fcgi?cmd=Retrieve&db=PubMed&list_uids=,11205743,&dopt=Abstract) |  | Direct | | [Proteasome subunit beta type 6](http://www.hprd.org/interactions?hprd_id=02630&isoform_id=02630_1&isoform_name=Isoform_1) |  | [In Vivo](http://www.ncbi.nlm.nih.gov/entrez/query.fcgi?cmd=Retrieve&db=PubMed&list_uids=,11205743,&dopt=Abstract) |  | Direct | | [Proteasome subunit beta, type 1](http://www.hprd.org/interactions?hprd_id=03603&isoform_id=03603_1&isoform_name=Isoform_1) |  | [In Vivo](http://www.ncbi.nlm.nih.gov/entrez/query.fcgi?cmd=Retrieve&db=PubMed&list_uids=,11205743,&dopt=Abstract) |  | Direct | | [Proteasome subunit beta, type 5](http://www.hprd.org/interactions?hprd_id=02629&isoform_id=02629_1&isoform_name=Isoform_1) |  | [In Vivo](http://www.ncbi.nlm.nih.gov/entrez/query.fcgi?cmd=Retrieve&db=PubMed&list_uids=,11205743,&dopt=Abstract) |  | Direct | | [Proteasome subunit, alpha type 3](http://www.hprd.org/interactions?hprd_id=01463&isoform_id=01463_1&isoform_name=Isoform_1) |  | [In Vivo](http://www.ncbi.nlm.nih.gov/entrez/query.fcgi?cmd=Retrieve&db=PubMed&list_uids=,11205743,&dopt=Abstract) |  | Direct | | [Protein kinase Myt1](http://www.hprd.org/interactions?hprd_id=03920&isoform_id=03920_1&isoform_name=Isoform_1) |  | [In Vitro](http://www.ncbi.nlm.nih.gov/entrez/query.fcgi?cmd=Retrieve&db=PubMed&list_uids=,12738781,&dopt=Abstract) |  | Direct | | [Protein regulator of cytokinesis 1](http://www.hprd.org/interactions?hprd_id=17899&isoform_id=17899_1&isoform_name=Isoform_1) |  | [In Vivo](http://www.ncbi.nlm.nih.gov/entrez/query.fcgi?cmd=Retrieve&db=PubMed&list_uids=,17351640,&dopt=Abstract) |  | Direct | | [RecQ protein 5](http://www.hprd.org/interactions?hprd_id=04806&isoform_id=04806_1&isoform_name=Isoform_1) |  | [Yeast 2 Hybrid](http://www.ncbi.nlm.nih.gov/entrez/query.fcgi?cmd=Retrieve&db=PubMed&list_uids=,16169070,&dopt=Abstract) |  | Direct | | [Tuberous sclerosis 1](http://www.hprd.org/interactions?hprd_id=05594&isoform_id=05594_1&isoform_name=Isoform_1) |  | [In Vivo](http://www.ncbi.nlm.nih.gov/entrez/query.fcgi?cmd=Retrieve&db=PubMed&list_uids=,16339216,&dopt=Abstract) |  | Direct | | [Tubulin alpha 1](http://www.hprd.org/interactions?hprd_id=01851&isoform_id=01851_1&isoform_name=Isoform_1) |  | [In Vivo ; In Vitro](http://www.ncbi.nlm.nih.gov/entrez/query.fcgi?cmd=Retrieve&db=PubMed&list_uids=,10191277,&dopt=Abstract) |  | Direct | | [Tubulin gamma 1](http://www.hprd.org/interactions?hprd_id=01853&isoform_id=01853_1&isoform_name=Isoform_1) |  | [In Vivo ; In Vitro](http://www.ncbi.nlm.nih.gov/entrez/query.fcgi?cmd=Retrieve&db=PubMed&list_uids=,10191277,&dopt=Abstract) |  | Direct | | [Tubulin, beta](http://www.hprd.org/interactions?hprd_id=01852&isoform_id=01852_1&isoform_name=Isoform_1) |  | [In Vivo ; In Vitro](http://www.ncbi.nlm.nih.gov/entrez/query.fcgi?cmd=Retrieve&db=PubMed&list_uids=,10191277,&dopt=Abstract) |  | Direct | | [Tumor protein, translationally controlled 1](http://www.hprd.org/interactions?hprd_id=15979&isoform_id=15979_1&isoform_name=Isoform_1) |  | [In Vivo ; In Vitro](http://www.ncbi.nlm.nih.gov/entrez/query.fcgi?cmd=Retrieve&db=PubMed&list_uids=,12167714,&dopt=Abstract) |  | Direct | | [WEE 1 tyrosine kinase](http://www.hprd.org/interactions?hprd_id=01907&isoform_id=01907_1&isoform_name=Isoform_1) |  | [In Vitro](http://www.ncbi.nlm.nih.gov/entrez/query.fcgi?cmd=Retrieve&db=PubMed&list_uids=,15070733,&dopt=Abstract) |  | Direct | | [C10orf3 protein](http://www.hprd.org/interactions?hprd_id=12569&isoform_id=12569_1&isoform_name=Isoform_1) |  | [In Vivo](http://www.ncbi.nlm.nih.gov/entrez/query.fcgi?cmd=Retrieve&db=PubMed&list_uids=,16198290,&dopt=Abstract) |  | Direct | | [Proteasome subunit beta, type 2](http://www.hprd.org/interactions?hprd_id=03708&isoform_id=03708_1&isoform_name=Isoform_1) |  | [In Vivo](http://www.ncbi.nlm.nih.gov/entrez/query.fcgi?cmd=Retrieve&db=PubMed&list_uids=,11205743,&dopt=Abstract) |  | Direct | | [Proteasome subunit beta, type 3](http://www.hprd.org/interactions?hprd_id=03709&isoform_id=03709_1&isoform_name=Isoform_1) |  | [In Vivo](http://www.ncbi.nlm.nih.gov/entrez/query.fcgi?cmd=Retrieve&db=PubMed&list_uids=,11205743,&dopt=Abstract) |  | Direct | | [Proteasome subunit, beta type, 4](http://www.hprd.org/interactions?hprd_id=03710&isoform_id=03710_1&isoform_name=Isoform_1) |  | [In Vivo](http://www.ncbi.nlm.nih.gov/entrez/query.fcgi?cmd=Retrieve&db=PubMed&list_uids=,11205743,&dopt=Abstract) |  | Direct | | [MCM3](http://www.hprd.org/interactions?hprd_id=04072&isoform_id=04072_1&isoform_name=Isoform_1) |  | [In Vivo](http://www.ncbi.nlm.nih.gov/entrez/query.fcgi?cmd=Retrieve&db=PubMed&list_uids=,15654075,&dopt=Abstract) |  | Direct | | [Proteosome subunit alpha type 1](http://www.hprd.org/interactions?hprd_id=04170&isoform_id=04170_1&isoform_name=Isoform_1) |  | [In Vivo](http://www.ncbi.nlm.nih.gov/entrez/query.fcgi?cmd=Retrieve&db=PubMed&list_uids=,11205743,&dopt=Abstract) |  | Direct | | [Proteosome subunit alpha type 6](http://www.hprd.org/interactions?hprd_id=04171&isoform_id=04171_1&isoform_name=Isoform_1) |  | [In Vivo](http://www.ncbi.nlm.nih.gov/entrez/query.fcgi?cmd=Retrieve&db=PubMed&list_uids=,11205743,&dopt=Abstract) |  | Direct | | [Proteasome subunit beta type 7](http://www.hprd.org/interactions?hprd_id=04940&isoform_id=04940_1&isoform_name=Isoform_1) |  | [In Vivo](http://www.ncbi.nlm.nih.gov/entrez/query.fcgi?cmd=Retrieve&db=PubMed&list_uids=,11205743,&dopt=Abstract) |  | Direct | | [Kinesin family member 23](http://www.hprd.org/interactions?hprd_id=05455&isoform_id=05455_1&isoform_name=Isoform_1) |  | [In Vivo](http://www.ncbi.nlm.nih.gov/entrez/query.fcgi?cmd=Retrieve&db=PubMed&list_uids=,8524282,&dopt=Abstract) |  | Direct | | [Proteasome subunit, alpha type, 7](http://www.hprd.org/interactions?hprd_id=05967&isoform_id=05967_1&isoform_name=Isoform_1) |  | [In Vivo](http://www.ncbi.nlm.nih.gov/entrez/query.fcgi?cmd=Retrieve&db=PubMed&list_uids=,11205743,&dopt=Abstract) |  | Direct | | [Golgi reassembly stacking protein 1](http://www.hprd.org/interactions?hprd_id=06038&isoform_id=06038_1&isoform_name=Isoform_1) |  | [In Vitro ; In Vivo ; Yeast 2 Hybrid](http://www.ncbi.nlm.nih.gov/entrez/query.fcgi?cmd=Retrieve&db=PubMed&list_uids=,11447294,&dopt=Abstract) |  | Direct | | [ASPM](http://www.hprd.org/interactions?hprd_id=08384&isoform_id=08384_1&isoform_name=Isoform_1) |  | [In Vitro](http://www.ncbi.nlm.nih.gov/entrez/query.fcgi?cmd=Retrieve&db=PubMed&list_uids=,11283617,&dopt=Abstract) |  | Direct | | [Proteasome subunit,alpha type,4](http://www.hprd.org/interactions?hprd_id=10168&isoform_id=10168_1&isoform_name=Isoform_1) |  | [In Vivo](http://www.ncbi.nlm.nih.gov/entrez/query.fcgi?cmd=Retrieve&db=PubMed&list_uids=,11205743,&dopt=Abstract) |  | Direct | | [NIR2](http://www.hprd.org/interactions?hprd_id=07497&isoform_id=07497_1&isoform_name=Isoform_1) |  | [In Vivo ; In Vitro](http://www.ncbi.nlm.nih.gov/entrez/query.fcgi?cmd=Retrieve&db=PubMed&list_uids=,15125835,&dopt=Abstract) |  | Direct | | [p53](http://www.hprd.org/interactions?hprd_id=01859&isoform_id=01859_1&isoform_name=Isoform_1) |  | [In Vivo ; Yeast 2 Hybrid](http://www.ncbi.nlm.nih.gov/entrez/query.fcgi?cmd=Retrieve&db=PubMed&list_uids=,16753148,&dopt=Abstract) |  | Direct | | [Catenin beta](http://www.hprd.org/interactions?hprd_id=00286&isoform_id=00286_1&isoform_name=Isoform_1) |  | [In Vitro](http://www.ncbi.nlm.nih.gov/entrez/query.fcgi?cmd=Retrieve&db=PubMed&list_uids=19001871&dopt=Abstract) |  | Direct | | [Baculoviral IAP repeat containing protein 6](http://www.hprd.org/interactions?hprd_id=05731&isoform_id=05731_1&isoform_name=Isoform_1) |  | [In Vivo](http://www.ncbi.nlm.nih.gov/entrez/query.fcgi?cmd=Retrieve&db=PubMed&list_uids=,18329369,&dopt=Abstract) |  | Direct | | [Tuberous sclerosis 1](http://www.hprd.org/interactions?hprd_id=05594&isoform_id=05594_1&isoform_name=Isoform_1) [Tuberin](http://www.hprd.org/interactions?hprd_id=01850&isoform_id=01850_1&isoform_name=Isoform_1) |  | [In Vivo](http://www.ncbi.nlm.nih.gov/entrez/query.fcgi?cmd=Retrieve&db=PubMed&list_uids=16339216&dopt=Abstract) |  | Complex | | | | --- | --- | --- | --- | --- | --- | --- | --- | --- | --- | --- | --- | --- | --- | --- | --- | --- | --- | --- | --- | --- | --- | --- | --- | --- | --- | --- | --- | --- | --- | --- | --- | --- | --- | --- | --- | --- | --- | --- | --- | --- | --- | --- | --- | --- | --- | --- | --- | --- | --- | --- | --- | --- | --- | --- | --- | --- | --- | --- | --- | --- | --- | --- | --- | --- | --- | --- | --- | --- | --- | --- | --- | --- | --- | --- | --- | --- | --- | --- | --- | --- | --- | --- | --- | --- | --- | --- | --- | --- | --- | --- | --- | --- | --- | --- | --- | --- | --- | --- | --- | --- | --- | --- | --- | --- | --- | --- | --- | --- | --- | --- | --- | --- | --- | --- | --- | --- | --- | --- | --- | --- | --- | --- | --- | --- | --- | --- | --- | --- | --- | --- | --- | --- | --- | --- | --- | --- | --- | --- | --- | --- | --- | --- | --- | --- | --- | --- | --- | --- | --- | --- | --- | --- | --- | --- | --- | --- | --- | --- | --- | --- | --- | --- | --- | --- | --- | --- | --- | --- | --- | --- | --- | --- | --- | --- | --- | --- | --- | --- | --- | --- | --- | --- | --- | --- | --- | --- | --- | --- | --- | --- | --- | --- | --- | --- | --- | --- | --- | --- | --- | --- | --- | --- | --- | --- | --- | --- | --- | --- | --- | --- | --- | --- | --- | --- | --- | --- | --- | --- | --- | --- | --- | --- | --- | --- | --- | --- | --- | --- | --- | --- | --- | --- | --- | --- | --- | --- | --- | --- | --- | --- | --- | --- | --- | --- | --- | --- | --- | --- | --- | --- | --- | --- | --- | --- | --- | --- | --- | --- | |
| --- | --- | --- | --- | --- | --- | --- | --- | --- | --- | --- | --- | --- | --- | --- | --- | --- | --- | --- | --- | --- | --- | --- | --- | --- | --- | --- | --- | --- | --- | --- | --- | --- | --- | --- | --- | --- | --- | --- | --- | --- | --- | --- | --- | --- | --- | --- | --- | --- | --- | --- | --- | --- | --- | --- | --- | --- | --- | --- | --- | --- | --- | --- | --- | --- | --- | --- | --- | --- | --- | --- | --- | --- | --- | --- | --- | --- | --- | --- | --- | --- | --- | --- | --- | --- | --- | --- | --- | --- | --- | --- | --- | --- | --- | --- | --- | --- | --- | --- | --- | --- | --- | --- | --- | --- | --- | --- | --- | --- | --- | --- | --- | --- | --- | --- | --- | --- | --- | --- | --- | --- | --- | --- | --- | --- | --- | --- | --- | --- | --- | --- | --- | --- | --- | --- | --- | --- | --- | --- | --- | --- | --- | --- | --- | --- | --- | --- | --- | --- | --- | --- | --- | --- | --- | --- | --- | --- | --- | --- | --- | --- | --- | --- | --- | --- | --- | --- | --- | --- | --- | --- | --- | --- | --- | --- | --- | --- | --- | --- | --- | --- | --- | --- | --- | --- | --- | --- | --- | --- | --- | --- | --- | --- | --- | --- | --- | --- | --- | --- | --- | --- | --- | --- | --- | --- | --- | --- | --- | --- | --- | --- | --- | --- | --- | --- | --- | --- | --- | --- | --- | --- | --- | --- | --- | --- | --- | --- | --- | --- | --- | --- | --- | --- | --- | --- | --- | --- | --- | --- | --- | --- | --- | --- | --- | --- | --- | --- | --- | --- | --- | --- | --- | --- | --- | --- | --- | --- | --- | --- | --- | --- | --- | --- | --- | --- |

**ZWINT**

|  | | 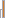 | **Protein Interactions** | 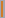 |  | | --- | --- | --- | --- |  |  | |  | | --- | | | **PROTEIN INTERACTORS** |  |  |  |  | | --- | --- | --- | --- | --- | | **Name of Interactor** |  | **Experiment Type** |  | **Type** | | [MIS12](http://www.hprd.org/interactions?hprd_id=12379&isoform_id=12379_1&isoform_name=Isoform_1) |  | [In Vivo ; In Vitro](http://www.ncbi.nlm.nih.gov/entrez/query.fcgi?cmd=Retrieve&db=PubMed&list_uids=,15502821,&dopt=Abstract) |  | Direct | | [ZW10](http://www.hprd.org/interactions?hprd_id=04902&isoform_id=04902_1&isoform_name=Isoform_1) |  | [In Vivo ; In Vitro](http://www.ncbi.nlm.nih.gov/entrez/query.fcgi?cmd=Retrieve&db=PubMed&list_uids=,15485811,10806105,15824131,16732327,&dopt=Abstract) |  | Direct | | [Chromosome 20 open reading frame 172 protein](http://www.hprd.org/interactions?hprd_id=16455&isoform_id=16455_1&isoform_name=Isoform_1) |  | [In Vivo](http://www.ncbi.nlm.nih.gov/entrez/query.fcgi?cmd=Retrieve&db=PubMed&list_uids=,15502821,&dopt=Abstract) |  | Direct | | [Kinetochore associated 2](http://www.hprd.org/interactions?hprd_id=06277&isoform_id=06277_1&isoform_name=Isoform_1) |  | [In Vitro ; Yeast 2 Hybrid](http://www.ncbi.nlm.nih.gov/entrez/query.fcgi?cmd=Retrieve&db=PubMed&list_uids=,16732327,&dopt=Abstract) |  | Direct | | [C1orf48 protein](http://www.hprd.org/interactions?hprd_id=12378&isoform_id=12378_1&isoform_name=Isoform_1) [Chromosome 20 open reading frame 172 protein](http://www.hprd.org/interactions?hprd_id=16455&isoform_id=16455_1&isoform_name=Isoform_1) [MIS12](http://www.hprd.org/interactions?hprd_id=12379&isoform_id=12379_1&isoform_name=Isoform_1) [Kinetochore associated 2](http://www.hprd.org/interactions?hprd_id=06277&isoform_id=06277_1&isoform_name=Isoform_1) [Kinetochore protein Spc24](http://www.hprd.org/interactions?hprd_id=15464&isoform_id=15464_1&isoform_name=Isoform_1) [Cancer susceptibility candidate 5](http://www.hprd.org/interactions?hprd_id=10634&isoform_id=10634_1&isoform_name=Isoform_1) [Lipoprotein, Lp like 2](http://www.hprd.org/interactions?hprd_id=17441&isoform_id=17441_1&isoform_name=Isoform_1) |  | [In Vivo](http://www.ncbi.nlm.nih.gov/entrez/query.fcgi?cmd=Retrieve&db=PubMed&list_uids=15824131&dopt=Abstract) |  | Complex | | [Kinetochore associated 2](http://www.hprd.org/interactions?hprd_id=06277&isoform_id=06277_1&isoform_name=Isoform_1) [ZW10](http://www.hprd.org/interactions?hprd_id=04902&isoform_id=04902_1&isoform_name=Isoform_1) |  | [In Vivo](http://www.ncbi.nlm.nih.gov/entrez/query.fcgi?cmd=Retrieve&db=PubMed&list_uids=16732327&dopt=Abstract) |  | Complex | | | | --- | --- | --- | --- | --- | --- | --- | --- | --- | --- | --- | --- | --- | --- | --- | --- | --- | --- | --- | --- | --- | --- | --- | --- | --- | --- | --- | --- | --- | --- | --- | --- | --- | --- | --- | --- | --- | --- | --- | --- | --- | --- | --- | --- | |
| --- | --- | --- | --- | --- | --- | --- | --- | --- | --- | --- | --- | --- | --- | --- | --- | --- | --- | --- | --- | --- | --- | --- | --- | --- | --- | --- | --- | --- | --- | --- | --- | --- | --- | --- | --- | --- | --- | --- | --- | --- | --- | --- | --- | --- | --- | --- | --- | --- | --- |
